# Supplementary material for: Professionals’ perspectives on interventions to reduce problematic alcohol use in older adults: a realist evaluation of working elements
Source: BMJ Open. 2024 Apr 15;14(4):e077851. doi: 10.1136/bmjopen-2023-077851 (PMC11029254; doi:10.1136/bmjopen-2023-077851)
Supplement: Supplementary data [file bmjopen-2023-077851supp001.pdf]

Supplementary Material 1

The COREQ Checklist

**Domain 1: Research team and reflexivity**		
Personal characteristics		
Interviewer/facilitator	Fieke van den Bulck	n/a
Credentials	MSc	Not reported in manuscript
Occupation	PhD	Not reported in manuscript
Gender	Woman	Not reported in manuscript
Experience and training	Conducted interviews as a part of her Ph.D.	Not reported in manuscript
Relationship with participants		
Relationship established	yes	Not reported in manuscript
Participant knowledge of the interviewer	Created relationship during preparation for the research study .	Not reported in manuscript
Interviewer characteristics		Not reported
**Domain 2: Study design**		
Theoretical framework		
Methodological orientation	Interviews with a realist evaluation approach	p. 5, 8
Participant selection		
Sampling	Convenience sampling	p. 6
Method of approach	Email or telephone	p. 4
Sample size	20 interviews with health care worker, prevention worker, coach, psychiatric mental health nurse, psychologist, peer support worker, volunteer, founder of intervention, advisor, coordinator, manager	p. 6, 7
Non-participation	5 did not participate	p. 6
Setting		
Setting of data collection	The interviews professionals were conducted via Microsoft Teams, Skype or telephone, between June and December 2022	p. 7
Presence of non-participants	No	Not reported
Description of sample		p. 6, 7
Data collection		
Interview guide		p. 7
Repeat interviews	No	Not reported in manuscript
Audio/visual recordings	Audio for Skype and telephone, visual recordings for Microsoft Teams interviews	Not reported in manuscript
Field notes	Yes	Not reported

Duration	The interviews ranged from 40.4 to 85.4 minutes	p. 7
Data saturation	Interviews were conducted until data saturation occured	p. 7
Transcripts returned	N/a	Not reported
**Domain 3: analysis and findings**		
Data analysis		
Number of data coders	2	p. 9
Description of the coding tree		p. 9
Derivation of themes	Themes were derived from data	p. 9
Software	Atlas.TI	p. 9
Participant checking	No	Not reported
Reporting		
Quotations presented	Yes, and each quotation is identified	p. 9-12
Data and findings consistent	Yes	
Clarity of major themes	Yes	p. 9-12, 14-17
Clarity of minor themes	Yes	p. 12-14
