# Supplementary material for: Professionals’ perspectives on interventions to reduce problematic alcohol use in older adults: a realist evaluation of working elements
Source: BMJ Open. 2024 Apr 15;14(4):e077851. doi: 10.1136/bmjopen-2023-077851 (PMC11029254; doi:10.1136/bmjopen-2023-077851)
Supplement: Supplementary data [file bmjopen-2023-077851supp003.pdf]

Supplementary Table 1. Comprehensive program theory

Context (C)	Element of intervention (E)	Mechanism (M)	Outcome (O)
**A. Practitioner – in-person – individual**	(No element, context only)	No misery others (II)<br>Practitioner functions as a big stick (II)<br>Dare to discuss things face to face better than online (IV)	
	1. Paying attention to drinking behavior		
	1.1 Paying attention to drinking behavior		
	1.1.1. Paying attention to drinking behavior	Thinking and awareness	Less or no alcohol use (X)
	1.1.2. Conversation with practitioner about alcohol use (X)		
	1.2 Tracking alcohol use and reflecting on use		
	1.2.1. Reflecting on alcohol use (II, X)	Insight to use (II)<br>Confrontation and reflection (II)	
	1.2.2. Tracking alcohol use (II)	Insight to use (II, III, IV)<br>Confrontation (II)	
	1.2.3. Paying attention to the cause of alcohol use (X)	-<br>Thinking about use/lifestyle (II)	Less or no alcohol use (X)<br>Awareness and insight alcohol use (II)
	1.3. Paying attention to abstinence, coping planning, and practicing with abstinence		
	1.3.1. Coping planning abstinence (I, IV, X)		
	1.3.2. Practicing with abstinence	-	Awareness alcohol use (IV)
	1.3.3. Raising awareness of responsible use/abstinence (I)	Awareness alcohol use (I)	
	1.4. Pointing out the risks and consequences of drinking behavior		
	1.4.1. Information about risks and consequences (I, II, IV, X)	Thinking about and recognize indirect consequences in own vulnerability (I, II)	Awareness of effects of alcohol use, less or no use (I, II)
		Shocked by consequences<br>Insight to use (IV)	Awareness of health effects of alcohol use (I)
		Increase risks perception for consequences Motivation to reduce	Less alcohol use (IV)
	2.Paying attention to lifestyle		
	2.1. Paying attention to meaning of life and teaching lifestyle changes		
	2.1.1. Attention and tips of meaning of life	Meaning in life	Less or no alcohol use (II)
	2.1.2. Teaching lifestyle changes (II)		
	2.1.3. Attention to different aspects of life (II)		
	3. Communication approaches		
	3.1. Motivational interviewing		
	3.1.1 Motivational interviewing (X, II)	Connect with client (X)	
		-	Awareness (X)
	3.2. Personalized content and conversation		
	3.2.1. Personalized content and conversation (I, II)		

4. The relationship between the client and practitioner			
4.1. Safety, trust, connection, and not being judged in contact with practitioner			
4.1.1. Client feels comfortable with practitioner (II)			
4.1.2. Low-threshold contact with practitioner (X)	-		Less or no alcohol use
4.1.3. Contact with practitioner feels safe	Opening conversation to discuss drinking behavior (X)		
4.1.4. Client feels accepted	Opening conversation to discuss drinking behavior (X)		
4.1.5. Client feels connection with practitioner	Opening conversation to discuss drinking behavior (X)		
4.1.6. Practitioner has an open attitude and does not judge	Client feels comfortable<br>-		Opening up at follow-up conversation (II)<br>Moderate drinking/awareness (II)
4.1.7. Good therapeutic relationship (X)			
4.1.8. Practitioner is friendly	Client feels comfortable		Openness at follow-up conversation (II)
4.2. Empathic behavior of practitioner			
4.2.1. Practitioner is empathic (I, X)	Client feels understood about alcohol-problems (X)	Client and practitioner collaborate in the identification of additional help, and the relationship between the client and the therapist improves	Less or no alcohol use (X)
	Client and practitioner collaborate in the identification of additional help, and the relationship between the client and the therapist improves (X)		
	Good relationship of trust (X)		
	Client becomes more honest and willing to discuss things (X)		
4.3. Critical, controlling, and confronting behavior of practitioner			
4.3.1. Practitioner has active role/guidance (II)	Engagement (II)		
4.3.2 Practitioner makes client reflect critically on client's actions (IV)	-		Less or no alcohol use (X)
4.3.3 Practitioner is critical/strict (II)			
4.3.4 Practitioner sets boundaries (IV)			
4.3.5 Practitioner confronts (II)			
5. Favorable setting			
5.1. Location is nearby home and/or familiar			
5.1.1. Location is nearby home and/or familiar	Low-threshold participation (I, IV)<br>Less effort to go the location		 Increase chances of participation (IV)
5.2. * Pleasant atmosphere			
5.2.1. Meeting with friendly and non-committal atmosphere	Clients can decide for themselves what they will and will not discuss (I)		
*6. * Other*			
6.1. Additional contact			

	6.1.1. Introductory interview	Alignment between practitioner and client		Client wants follow-up interview (II)
	6.1.2. Multiple sessions	More attention for (different) themes and needs (II)		
	6.1.3. Conversation with general practice-based nurse specialist/GP (II)			
	6.2. Other characteristics/input practitioner			
	6.2.1. Practitioner has much knowledge	Thinking about alcohol use		Motivation less alcohol use(X)
	6.2.2. Practitioner shares own experience	Equal contact	Connection between practitioner and client	Client is open for what practitioner has to offer (IV)
**B. Practitioner – not in-person – individual**	(No element, context only)	Practitioner functions as a big stick (X)		
	1. Communication approaches			
	1.1. Contact always possible			
	1.1.1.Always the possibility of contact (I)	Accessible help in difficult situations		Less or no alcohol use, think differently about alcohol use, being open about alcohol use (I, III)
	1.2. Conversation over the phone			
	1.2.1. Telephone conversation (I)	Accessible and flexible (distance, time and anonymity) (I, X)		
		Responding to emotions (I)		
	1.3. Anonymous			
	1.3.1. Anonymous and telephone conversation (I)	Distance between practitioner and client	Client is more likely to accept advice (I)	
			Client speaks and opens up easier (I)	
		Low-threshold (I)		
		Less attention, shame (I)		
		-		Think differently about alcohol (I)
		-		Being open about alcohol use (I)
	1.3.2. No relationship between client and practitioner	Honest instead of socially desirable contact		Think differently about alcohol use (I)
				Being open about alcohol use (I)
	1.4. Motivational interviewing			
	1.4.1. Motivational interviewing	Engagement		Awareness alcohol use (I, X)
		Open communication and client accept advice from practitioner (I)		
	1.5. Personalized content and conversation			
	1.5.1. Personalized content and conversation (I)	Tailoring to needs and level (I)		Increase knowledge and motivation (I)
		Non-commitment disappears		Less or no alcohol use (III)
		-		Knowledge about where to go when seeking help, learning to cope with alcohol use, increasing motivation for less or no alcohol use (I)
		Pick up emotions (I)	Establish a relationship	Communicate message better to client (I)
				Make the client think critically (I)
		Possibility of attention other problems (III)		Insight into the cause of the problem (III)

		Feeling of support		Less or no alcohol use (III, X)
	1.6. Personal contact and feedback			
	1.6.1. Personal contact and feedback (X)	Non-commitment disappears (X)		Less or no alcohol use (X)
	1.7. * Other			
	1.7.1. Five-phase model	Open communication and clients accept advice from practitioner (I)		Awareness alcohol use (I)
	1.7.2. Structure, clear expectations and agreements for client (III)	A big stick		Awareness alcohol use (III)
	1.7.3. Regular contact	A big stick	Motivation	Awareness alcohol use, less or no alcohol use (III)
	2. The relationship between the client and practitioner			
	2.1. Practitioner is empathic, supportive, and listens			
	2.1.1 Gentle approach with compliments (I)			
	2.1.2 Practitioner does not judge about alcohol behavior, supports and listen (I)			
	2.1.3 Practitioner support and listens (I)			
	2.1..4 Practitioner is empathic	Engagement	Clients accept information from practitioner<br>Open communication	Client will seek help from addiction services (I)
	2.2. * Practitioner takes away the shame with positive approach	Reduce craving for alcohol (I)		
	3. Providing additional help			
	3.1. Personal treatment with practitioner additional to self-help			
	3.1.1 Personal treatment with practitioner additional to selfhelp (III, X)	Working independent (III)<br>Keep more attention<br>Provide extra tailoring (III)<br>Possibility of involving relatives (III)<br>Recognition (III)		More likely to persist (X)
	*4. *Paying attention to drinking behavior*			
	4.1. Information about risks and possible help			
	4.1.1. Information about health risks	-		Increase knowledge about alcohol (I)
	4.1.2. Information about alcohol interventions (I)			
**C. Practitioner—in-person—relatives**	*1. Teaching the partner to deal with drinking behavior*			
	1.1 Teaching the partner to deal with drinking behavior			
	1.1.1. Teaching the partner about/dealing with drinking behavior(I, IV, X)	Partner gets more trust in client<br>More understanding and support from the relative for the client (X)		Growing together and taking steps (IV)
	2. Support of relatives			
	2.1 Relative provides support			
	2.1.1.Relatives are deployed to provide support (X)	-		Less or no alcohol use
	*3. * Other*			

	3.1. Relative controls			
	3.1.1. Relative gives or controls information (II)			
	3.2. Partner shares experience and insights with other partners			
	3.2.1 Partner shares experience and insights with other partners	Partner is relaxed and is less controlling		Easier for client to achieve goals (IV)
**D. Practitioner – in-person – group component**	(No element, context only)	Togetherness, connection, recognition (I)	Sharing 'stick behind the door' experience	Know how to improve quality of life during abstinence, abstinence (IV)
			-	Less or no alcohol use (IV)
		Recognition		Achieving goals (IV)
		Location gets people outside where addiction takes place (IV)		
		Safety (V)		
		Free to share things by others talking (I)		
	1. Paying attention to drinking behavior			
	1.1. Pointing out the risks and consequences of drinking behavior			
	1.1.1. Information about risk and consequences (IV, V)	Insight in addiction (IV)		
	1.2. Paying attention to abstinence and practicing with abstinence			
	1.2.1. Attention and tools for (sustained) abstinence	-		Less or no alcohol use (IV)
		Less alcohol use	Mood improves	People become more active and anxiety symptoms will reduce (IV)
	1.2.2. Practicing with new or tense situations	Learning to tolerate discomfort in life (IV)		
		Gain experience (IV)		
	1.3. *Discuss statements on alcohol use in groups			
	1.3.1. Discuss statements in groups	Thinking about alcohol use (I)		
		Openness about alcohol use (I)		
	2. Paying attention to lifestyle			
	2.1. ** Motivating to change lifestyle			
	2.2. Attention and tips regarding meaning of life, lifestyle, and problems when aging			
	2.2.1 Attention for meaning of life and life stage themes (IV, X)	Motivation to go to the next session (IV)		
		Better coping with cravings for alcohol (IV)		
		Motivation for change		Abstinence, know how to improve quality of life during abstinence (IV)
	2.1.2. Attention for lifestyle or motivate lifestyle change (X)	-		Less alcohol use (X)
	2.3. **Motivating to change lifestyle delivered in a workplace setting			

2.4. In a workplace setting and paying attention to prevention and lifestyle			
2.4.1. At work (X)	Increase awareness (X)		
2.4.2. At work with attention for prevention (X)			
2.4.3. At work with attention for lifestyle (X)			
2.4.4. * At work, openness about alcohol use	Negative consequences (X)		
	Support environment (X)		
	-		Less alcohol use (X)
2.4.5. * At work, people join national campaign (X)			
3. Communication approaches			
3.1. Motivational interviewing			
3.1.1. Motivational interviewing	-		Less or no alcohol use, awareness (X, IV)
3.2. Personalized content and conversation			
3.2.1. Personalized content and conversation (IV, X)			
3.2.2. * Customized CBT treatment (IV)			
3.3. * Other approaches			
3.3.1. Change way of thinking from negative to positive (IV)	Rethinking and self-pity reduces	Getting stronger to prevent relapse	Less or no alcohol use (IV)
3.3.2. Overcoming shame			Less or no alcohol use (III)
3.3.3. Mindfulness and running therapy (V)	Feeling signals in body and recognize (V)		
3.3.4. CGT focusing on substance use (IV)	-		Less or no alcohol use
3.3.5. Structure in days	Having rhythm and purpose to go somewhere (V)		
3.3.6. Variance of working methods (IV)			
4. The relationship between the client and peers			
4.1.Contact with peers			
4.1.1. Contact with peers (IV, IV, IV, X)	Being seen and feel supported (IV)		
	Connection (IV)		
4.1.2. Group composition like-minded peers (IV, IV)	Recognition and connection (IV,V, X)		
4.2. Closed group and maxim group size			
4.2.1.Maximized group size	Creating safety		Preventing drop out (IV)
	More attention per client (I, IV)		
4.2.2.Closed group	Improves atmosphere (IV)		
	Ongoing process (IV)		
	Safety	Daring to open up (IV)	
4.3. Engagement, understanding, and support towards peers			
4.3.1. Engagement and understanding each other (IV)	Connection (IV)		
	Safety	Talking about problems (IV)	Less or no alcohol use (IV)
	Togetherness, being able to ask for help from each other (IV)		

4.3.2. Support each other (V)			
4.3.3. People feeling heard in group	Less alcohol use	Mood improves	People become more active and anxiety symptoms will reduce (IV)
4.4. Addressing peers			
4.4.1. Addressing each other, reflect critically on your own actions (II, V)	Learning and getting insights		Less or no alcohol use (III, IV)
4.5. Sharing experiences and tips with peers			
4.5.1. Sharing experiences and tips (V, X)	Hopeful for own process (IV, V)		
	Openness about alcohol use (IV)		
	Recognition and acknowledgement (IV)		
	Putting own situation in perspective (IV)		
	Feeling the impact of addiction (IV)		
4.6. * Making agreements with peers			
4.6.1. Making agreements among themselves in group	A big stick (IV)		
5. The relationship between the client and practitioner			
5.1. Open attitude, not being judged and accessible contact with practitioner			
5.1.1. Practitioner is unbiased, open and not moralizing (V)			
5.1.2. Accessible contact practitioner	Experience support		Less relapse in alcohol use (V)
5.2. * Other			
5.2.1. Practitioner has the same age of clients (IV)	Recognition and click (IV)		
5.2.2. Presence two practitioners (IV)			
5.2.3. Interactive contact	Holding attention (I)		
5.2.3. Guest speakers focusing on different lifestyle themes (IV)	-		Abstinence, know how to improve quality of life during abstinence (IV)
6. Other activities			
6.1. Having lunch after every session			
6.1.1. After every session have a lunch together (IV, V)	Connection (IV)		Less or no alcohol use (IV)
	Atmosphere in the group (IV)		
	Unforced atmosphere		Discuss topics in a low-threshold way that are more difficult during a session (IV)
			Safe setting (IV)
	Relaxation and getting to know each other better (IV)		
	Insight of simplicity nutritious meal (IV)		
6.2. * Activities			
6.2.1. Social activities (V)	Daytime activities	Reduce loneliness (IV)	
6.2.2. Outdoor intervention in group walking with app	Stimulate to move or exercise		Better coping with cravings for alcohol (IV)

	7. Favorable setting			
	7.1. Pleasant and relaxed atmosphere			
	7.1.1. Meeting with friendly and non-committal atmosphere	Clients can decide for themselves what they will and will not discuss (I)		
	7.1.2. Besides serious atmosphere, also relaxed atmosphere with humor (IV)			
	7.2. Location is nearby home and/or familiar			
	7.2.1. Location is nearby home and/or familiar	Low-threshold participation (I, IV)		
		Less effort to get to location	Increase chance of participation (IV)	
	7.3. * Location is not admission clinic			
	7.3.1. Location is not admission clinic	People are not remembered by their admission (V)		
**E. No practitioner – not in-person - individual**	*1. Paying attention to drinking behavior*			
	1.1. Tracking alcohol use			
	1.1.1.Monitoring alcohol use	Keep track of progress		Less alcohol use (III)
	1.1.2. Tracking alcohol use in app/telephone	Insight into alcohol use(X)		Less or no alcohol use (X)
	1.1.3. * Information and tools relapse and dealing with difficult moments	Prevent relapse	Handle difficult moments better and consciously (III)	
	1.2. Abruptly quitting alcohol use			
	1.2.1. Abruptly quit alcohol use for a temporary period (III)	Learning experience that can be perceived as positive (II)	High self-efficacy and attitude change towards alcohol use (III)	High probability that people will use less or no alcohol use later (again) (III)
		Awareness behavioral change (III)		
	1.3. Pointing out the risks and consequences of drinking behavior			
	1.3.1. Paying attention to risks of alcohol use at age	Get people thinking (III)		
	1.4. * Receiving feedback			
	1.4.1. Receiving feedback	Motivation		Less alcohol use (III)
	1.4.2. Positive feedback	Motivation		Less alcohol use (III)
	2. Using tools			
	2.1. ** Web based interventions			
	2.2. Online self-help tool			
	2.2.1.App	No waiting list so quick participation (III)		
		Getting started with themes in a low-threshold way (X)		
	2.2.2. App that is simple and clear	Accessible (III)		
	2.2.3. Online self-help tool /program /module	Own direction implementation (III)		Less or no alcohol use (X)
		Low-threshold(III)		
		-		Awareness/ Less or no alcohol use/ Step towards treatment (IV)
	2.2.4. Short online module with playful approach (III)	Awareness		Small changes alcohol use (III)

	2.3. Via telephone		
	2.3.1. Via telephone	Saving time/cost (X)	
	2.4. Regular newsletter		
	2.4.1. Regular newsletter (III)	Being actively engaged Support (III)<br>Recognition support (III)	
		-	Being motived to use less or no alcohol use for longer periods of time (III)
	2.5. * Other		
	2.5.1. Webinar with peer chatbox	Personal contact what serves as social support (III)	
	3. The relationship between the client and peers		
	3.1. Joining with others, contact with peers		
	3.1.1. Additional contact with peers (III)		
	3.1.2. Joining with others (III)	Feeling of togetherness (III)<br>Feeling of recognition (III)<br>Social support (III)	Increase success of intervention (III)
	4. Favorable setting		
	4.1. Participation independent of location		
	4.1.1. Participation independent of location /participation possible from home (III)	Accessible and low-threshold (III)	
	5. Additional help		
	5.1. Personalized content and help additional to self-help		
	5.1.1. Additional personal coaching at self-help interventions (III)		
	5.1.2. Additional physical tools for client (III)	Feeling of physical participation at home (III)	
	5.1.3. Extra options for support (III)	Feeling of personal, voluntary and non-committal participation	Less or no alcohol use for one month (III)
	*6. * Other*		
	6.1. Attention other themes		
	6.1.1. Attention to activity spending	Get people thinking (III)	
	6.1.2. Attention for difficult aspects in life	Get people thinking (III)	
	6.1.3. Commitment with yourself by signing up (III)	Motivation	Increase success of intervention (III)
	6.1.4. Non commitment participation	Low-threshold (III)	Makes less or no alcohol use pleasant, step up to intensive help (III)
		Extra motivation (III)	
	6.1.5. Attention to positive experiences	Increase self-efficacy (III)	
	6.1.6. Setting goals	-	Less alcohol use (III)
**F. No practitioner – not in-person – group component**	(No element, context only)	Motivation and not being alone	Less or no alcohol use (X)
	1. Paying attention to drinking behavior		
	1.1. **Intervention to abstinent people		
	1.2. Focus on abstinence and paying attention to withdrawal		

1.2.1. Focus on abstinence, with attention to withdrawal (X)		
1.2.2. Focus on abstinence (X)		
2. Self-help groups		
2.1. AA and self-help group		
2.1.1. Aa/self-help group (X)	-	Less or no alcohol use (II)
3. The relationship between the client and peers		
3.1. Contact with peers		
3.1.1. Peer contact (IV, X)	Recognition (X)	
3.2. Sharing experiences and tips with peers		
3.2.1. Sharing experiences and tips with peers (III)	Hope and connection (X)<br>Persuade to participate (X)<br>Stimulate (X)	
3.3. * Other		
3.3.1. Composition of people with same level/serious issues (III)		
3.3.2. Making agreements and setting goals in group	-	Less or no alcohol use (X)
3.3.3. Helping each other (X)		
3.3.4. Online forum	Social support	Temporary stop of alcohol use (III)
*4. * Paying attention to other aspects*		
4.1. Broader focus than abstinence		
4.1.1. Broader focus than only abstinence	Thinking about what is important	Less or no alcohol use (X)

*= Program theories that were not included in Table 3. ** Initial program theories that were refined or not confirmed.
I = People with all types of alcohol use patterns; II = people with early stage problematic alcohol use; III = people with all types of alcohol use patterns, except for (heavy) problematic alcohol use or alcohol addiction; IV = people with problematic alcohol use and alcohol addiction; V = people who are in recovery from addiction. X = No target group mentioned because these elements were derived from CEMO configuration that we presented as statements during the interviews.
