# Supplementary material for: Professionals’ perspectives on interventions to reduce problematic alcohol use in older adults: a realist evaluation of working elements
Source: BMJ Open. 2024 Apr 15;14(4):e077851. doi: 10.1136/bmjopen-2023-077851 (PMC11029254; doi:10.1136/bmjopen-2023-077851)
Supplement: Author's manuscript [file bmjopen-2023-077851.draft_revisions.pdf]

BMJ Open is committed to open peer review. As part of this commitment we make the peer review history of every article we publish publicly available.

When an article is published we post the peer reviewers' comments and the authors' responses online. We also post the versions of the paper that were used during peer review. These are the versions that the peer review comments apply to.

The versions of the paper that follow are the versions that were submitted during the peer review process. They are not the versions of record or the final published versions. They should not be cited or distributed as the published version of this manuscript.

BMJ Open is an open access journal and the full, final, typeset and author-corrected version of record of the manuscript is available on our site with no access controls, subscription charges or pay-per-view fees (http://bmjopen.bmj.com).

If you have any questions on BMJ Open's open peer review process please email info.bmjopen@bmj.com

BMJ Open

Professionals' perspectives on interventions to reduce and prevent problematic alcohol use in older adults: A realist evaluation of working elements.

Journal:	*BMJ Open*
Manuscript ID	bmjopen-2023-077851
Article Type:	Original research
Date Submitted by the Author:	17-Jul-2023

Complete List of Authors:	van den Bulck, Fieke; Tilburg University Tranzo Scientific Centre for Care and Welfare, Tranzo<br>Knijff, Rikste; Tilburg University Tranzo Scientific Centre for Care and Welfare, Tranzo<br>Crutzen, Rik; School for Public Health and Primary Care (CAPHRI), Department of Health Promotion<br>van de Mheen, D; Tilburg University,<br>Bovens, Rob; Tilburg University Tranzo Scientific Centre for Care and Welfare, Tranzo<br>Stutterheim, Sarah; Maastricht University, Department of Work and Social Psychology<br>Van de Goor, Ien ; Tilburg University Tranzo Scientific Centre for Care and Welfare<br>Rozema, A; Tilburg University,

Keywords:	Aged, Health Services for the Aged, QUALITATIVE RESEARCH

SCHOLARONE™
Manuscripts

I, the Submitting Author has the right to grant and does grant on behalf of all authors of the Work (as defined in the below author licence), an exclusive licence and/or a non-exclusive licence for contributions from authors who are: i) UK Crown employees; ii) where BMJ has agreed a CC-BY licence shall apply, and/or iii) in accordance with the terms applicable for US Federal Government officers or employees acting as part of their official duties; on a worldwide, perpetual, irrevocable, royalty-free basis to BMJ Publishing Group Ltd ("BMJ") its licensees and where the relevant Journal is co-owned by BMJ to the co-owners of the Journal, to publish the Work in this journal and any other BMJ products and to exploit all rights, as set out in our licence.

The Submitting Author accepts and understands that any supply made under these terms is made by BMJ to the Submitting Author unless you are acting as an employee on behalf of your employer or a postgraduate student of an affiliated institution which is paying any applicable article publishing charge ("APC") for Open Access articles. Where the Submitting Author wishes to make the Work available on an Open Access basis (and intends to pay the relevant APC), the terms of reuse of such Open Access shall be governed by a Creative Commons licence – details of these licences and which Creative Commons licence will apply to this Work are set out in our licence referred to above.

Other than as permitted in any relevant BMJ Author's Self Archiving Policies, I confirm this Work has not been accepted for publication elsewhere, is not being considered for publication elsewhere and does not duplicate material already published. I confirm all authors consent to publication of this Work and authorise the granting of this licence.

For peer review only

Professionals' perspectives on interventions to reduce and prevent problematic alcohol use in older adults: A realist evaluation of working elements.

F.A.E. van den Bulck[1], R. Knijff[1], R. Crutzen[2], D. van de Mheen[1], R.H.L.M. Bovens[1], S.E. Stutterheim[2], L.A.M. van de Goor[1], & A.D. Rozema[1]

[1] Tranzo Scientific Center for Care and Wellbeing, School of Social and Behavioral Sciences, Tilburg University, the Netherlands.

[2] Department of Health Promotion, Care and Public Health Research Institute (CAPHRI), Maastricht University, the Netherlands.

Affiliation addresses

[1] Professor Cobbenhagenlaan 125, Postbus 90153, 5000 LE Tilburg, The Netherlands

[2] Peter Debyeplein 1, 6229 HA Maastricht, The Netherlands

Corresponding author

F.A.E. van den Bulck

Email: f.a.e.vdnbulck@tilburguniversity.edu

Address: Professor Cobbenhagenlaan 125, Postbus 90153, 5000 LE Tilburg, The Netherlands

Abstract

Objectives

This study set out to understand how (i.e., which elements), in which context, and why (which mechanisms) interventions are successful in preventing or reducing (problematic) alcohol use among older adults, from the perspective of professionals providing these interventions.

Design,

Guided by a Realist Evaluation approach, an existing initial program theory (IPT) on working elements in alcohol interventions was evaluated by conducting semi-structured interviews with professionals.

Setting and participants These professionals (N=20) provide interventions across several contexts: with or without practitioner involvement; in-person or not; and in an individual or group setting. Data were

For peer review only

coded and links between contexts, elements, mechanisms and outcomes were sought for to confirm, refute or refine the IPT.

Results

From the perspective of professionals, there are several general working elements in interventions for older adults: 1) *pointing out risks and consequences of drinking behavior*, 2) *paying attention to abstinence*, 3) *promoting contact with peers*, 4) *providing personalized content* and 5) *providing support*. We also found context-specific working elements, i.e., 1) *providing personalized conversations* and *motivational interviewing* with practitioners, 2) *ensuring safety, trust and a sense of connection* and *a location nearby home or a location that people are familiar with* in-person, and 3) *sharing experiences and tips* in group interventions. Furthermore, the mechanisms *awareness* and *low-threshold/accessibility* were important contributors to positive intervention outcomes.

Conclusion

In addition to the IPT, our findings emphasize the need for social contact and support, personalized content, and strong relations (both between client and practitioner, and client and peers) in interventions for older adults.

Strengths and limitations of this study

Strengths and limitations of this study

- Using a Realist Evaluation provides in-dept insights in interventions because it can explain why interventions are successful or not.
- Data were collected through interviews with a variety of professionals across eleven different alcohol interventions
- Habitation bias and acquiescence bias could have occurred in data collection, which may influence the confirmed IPT.

For peer review only

INTRODUCTION

In recent decades, drinking behavior has increased, particularly in older adults (1-5). This has been accompanied with increased problematic alcohol use including high-risk drinking, binge drinking, and alcohol use disorders (6-8). Moreover, this change in drinking patterns has been associated with increased alcohol-related health problems in older adults due to biological changes in metabolism, body fat, body water, and the reduced ability of the liver to process alcohol (9, 10), and due to the combination alcohol with (increasing amounts of) medication when aging (11).

This increase in problematic alcohol use among older adults emphasizes the need for effective interventions. There are several existing interventions to reduce alcohol use, such as cognitive behavioral therapy (CBT) or motivational enhancement therapy, that are considered to be appropriate for older adults (12-15). However, most interventions to reduce or prevent (problematic) alcohol use in which older adults participate are designed for the general adult population; there is no consensus yet on what works best for older adults specifically (16, 17). Nonetheless, two systematic reviews found that effective intervention components to reduce alcohol use among older adults are personalized feedback, advice on (alcohol) behaviors, educational materials, and counseling (18, 19).

A systematic review which included 61 studies – using Realist Evaluation – focused on understanding how, in which context, and why interventions are successful in preventing or reducing (problematic) alcohol consumption among older adults (20). This review explored what works for people of 18+ individuals years and older (which includes older adults), and older adults specifically, and found working elements across six different contexts.

First, in interventions that were delivered by a practitioner, in-person and to one individual (instead of a group), the working elements *paying attention to drinking behavior* and *the relationship between the client and practitioner* were identified. Second, in interventions that were delivered by a practitioner, not in-person and individual, *personal contact and feedback* and *online communication and feedback* were identified. Third, in interventions that were delivered by a practitioner, in-person and with involvement of relatives, *status of the relationship* and *teaching the partner to deal with drinking behavior* were identified. Fourth, in interventions that were delivered by a practitioner, in-person and in group settings, *motivating to change lifestyle* (delivered in a workplace setting) was identified. Fifth, in interventions that were delivered without a practitioner and individual, *web based* and *telephone based* interventions were working elements. Sixth, in interventions that were delivered without a practitioner, not in-person and in group setting, *focus on abstinence* was identified.

Overall, the results of the review showed three general working elements, i.e., 1) providing information about the consequences of alcohol consumption; 2) personalized feedback about drinking behavior; and 3) being in contact with others and communicating with them about (alcohol) problems (20). Of the 61 studies, only three evaluated interventions for older adults, and only the first and second general working element were found in these studies (21-23).

Evidently, in light of the small number of studies conducted specifically among older adults, more research on what works for older people is needed. The current study builds further on the previous performed review using Realist Evaluation.

Realist evaluations (RE) work from the assumption that interventions and their elements (E) work differently in different contexts (C). This means that interventions may be successful in some contexts and not in others because the mechanisms (M) needed for success are triggered to a different extent and lead to different outcomes (O). The strength of RE is that it offers a means to unpack the relationships between these contexts, elements, mechanisms, and outcomes. This is called the Context-Element-Mechanism-Outcome (CEMO) – configuration (24). RE starts with formulating an Initial Program Theory (IPT), based on the CEMOs founded in literature. Then the IPT needs to be confirmed, refused or refined using data that seeks to explain how the interventions work in real life contexts from the perspectives of those involved in its operation. Finally, based on the new data, the program theory might be refined.

This study is set out to confirm, refuse, or refine the findings of the formed IPT of Boumans et al. (22), to understand from the perspective of professionals providing these interventions how (i.e., which elements), in which context, and why (which mechanisms) interventions are successful in reducing (problematic) alcohol consumption among older adults. The perspective of professionals was considered important as they have extensive experience in addressing problematic alcohol use among older adults and a comprehensive understanding of what and why it works. Based on the perspectives of the professionals, CEMOs of the IPT will be confirmed, refuted or refined.

METHOD

Study context and design

For peer review only - http://bmjopen.bmj.com/site/about/guidelines.xhtml

In this qualitative study, we conducted semi-structured interviews with professionals providing interventions to older adults. This study is part of a larger qualitative research project that also investigates the perspectives of clients and close relatives of clients. Here, we focus on the professionals' perspectives. The larger study was approved by the Ethics Review Board at Tilburg University (RP508) and the COREQ guidelines were followed in reporting and design (25), see Supplementary Material 1.

Sampling and recruitment

In the current study, we set out to recruit and interview professionals providing various interventions across different organizations throughout the Netherlands.

First, we inventoried which interventions were used in practice by searching the Dutch National Database Centre for Healthy Living (*Centrum Gezond Leven*) and by consulting the national working group Elderly in the Dutch Partnership for Early Detection of Alcohol Problems (*Samenwerkingsverband Vroegsignalering Alcoholproblematiek*) and the national working group Alcohol and Elderly of The Dutch Addiction Association (*Verslavingskunde Nederland*). These two working groups comprise professionals from various organizations providing different interventions.

Second, based on this inventory, we included interventions if they 1) focused on universal, selective or indicated prevention; 2) were provided in an individual and/or group setting with or without relative involvement; 3) were provided face to face, online, and/or via telephone; 4) were provided in the Netherlands. Interventions were excluded if they 1) were not primarily aimed at lifestyle change; 2) were provided in a clinical setting; 3) were group-specific interventions (e.g., for veterans or athletes); or 4) focused on general health and lifestyle improvements without explicit mention of alcohol consumption.

Based on these selection criteria, the following Dutch alcohol interventions were included: Alcohol Information Line (*Alcohol Infolijn*), CBT for substance use (*CGT bij middelengebruik*), Enjoy Healthy (*Gezond Genieten*), Fresh Onwards, (*Fris Verder*), Maxx, Mirro, Moti-55, NoThanks (*IkPas*), Recovery-Guided Personal Program (*Herstel Ondersteunend Persoonlijk Programma*), Vitality days (Vitaliteitsdagen), Snowball sampling among professionals helped us identify one other intervention: Drink Less (*MinderDrinken*)

Third, we approached professionals of the two working groups either working with these interventions or we approached professionals at organizations working with the interventions and asked to connect us with professionals that were involved in providing the interventions. Professionals that

For peer review only - http://bmjopen.bmj.com/site/about/guidelines.xhtml

were interested received information about the study via email and some received information by telephone as well. They all received an information letter and informed consent form. Subsequently, they were invited for an interview.

In total, we contacted 25 professionals, of whom 20 participated. The remaining five did not respond to our emails. Participants' characteristics are shown in Table 1. Characteristics of included interventions are shown in Table 2.

Table 1. Participants' characteristics

Baseline characteristic	Participants<br>*n* = 20
Gender	
Women	15
Men	5
Age (years)	
25-39	6
40-54	4
55-69	7
≥ 70	3
Experience with the intervention (in years)	
<1	2*
1	3
2	4
3	2
4	4
5	3
6	1
7	-
8	-
9	1
Role**	
Health care worker, prevention worker, coach, psychiatric mental health nurse or (GZ) psychologist	13
Peer support worker	1
Volunteer	3
Founder of intervention	3
Advisor, coordinator, manager	3
Intervention	
Alcohol Information Line	3
CBT for substance use	2
DrinkLess	1
Enjoy Healthy	1
Fresh Onward	4
Maxx	1

For peer review only

Mirro	1
Moti-55	2
NoThanks	2
Recovery guided personal program	1
Vitality days	2

* One professional was only involved in the development of the intervention.

** Some professionals had multiple roles in the same interventions, e.g. working as a coach and coordinator.

For peer review only

Table 2. Intervention characteristics

Intervention	Contact in intervention with			Setting			Sessions			Target group intervention	
	Profession al	Peers	Relatives	Online, telephone	In-person	Anonymous	1	>1	N/a	Alcohol use**	Olde adul (55 or 65+
Alcohol Information line	X			X		X	X	X*		I	
CBT for substance use	X	X	X*		X			X		IV	X
Drink Less	X*			X		X*			X	III	
Enjoy Healthy	X	X			X		X			I	X
Fresh Onwards	X	X	X		X			X		IV	X
Maxx				X		X			X	III	
Mirro				X		X			X	III	
Moti-55	X		X*		X			X		II	X
NoThanks	X*			X		X*	X*	X*	X	III	
Recovery guided personal program	X		X*		X			X		V	
Vitality days	X	X*	X*		X		X			I	X

* = Optional, depending on client's preference

** I = People with all types of alcohol use patterns, II = People with early stage problematic alcohol use; III = People with all types of alcohol use patterns, except for (heavy) problematic alcohol use or alcohol addiction; IV = People with problematic alcohol use and alcohol addiction; V = People who are in recovery from addiction

For peer review only - http://bmjopen.bmj.com/site/about/guidelines.xhtml

Data collection

Based on the participants preferences, online (80%) and telephone (20%) interviews were conducted between June and December 2022 with a median of 56.7 minutes (ranging from 40.4 and 85.4 minutes). Demographic variables including age, gender, and some questions about the intervention were asked prior to the interviews. All interviews were preceded by oral informed consent. We used an interview guide that measured the perceived outcomes of the interventions, the working elements and mechanisms related to the outcome(s), and the influence of the setting in which the interventions were provided. At the end of the interview guide, we presented the previous found CEMO configurations of the IPT of the review of Boumans et al. and invited participants to confirm, refute or refine the CEMOs and asked if they could comment on the CEMOs, see interview guide (see Supplementary Material 2).

Data analysis

Professional transcriptionists transcribed the interviews verbatim. Based on the IPT, FvdB drafted a code tree including CEMO configurations. Researchers FvdB and RK independently and, in parallel, coded six of the 20 interviews based on the RE approach, which means that the links between contexts, elements, mechanisms, outcomes were sought for within the data. After the first transcript was coded by FvdB and RK, FvdB cross-checked both their configurations and codes. FvdB and RK iteratively discussed differences in configurations or coding, and where relevant, refinements were made. These steps were repeated for all of the six initial interviews, at which time, coding was fully aligned. Thereafter, FvdB coded the remaining interviews independently. When in doubt about how to best code sections of the data, FvdB consulted with RK and ADR. Additionally, to further improve the quality of the coding, RK, RC, RB, IvdG, SS, and ADR reviewed formed codes and CEMOs iteratively and provided feedback. Codes were then adjusted if necessary and some new codes were added. Data were analysed using Atlas.TI.

RESULTS

Results are categorized according to the context in which the interventions are delivered: 1) involvement or lack of involvement of a practitioner that provides the intervention directly to the participant; 2) provision of the intervention in-person or not; and 3) individual treatment, group treatment or treatment with relatives' involvement. We found data across six combinations of contexts. Table 3 provides an overview of the key working elements, mechanisms, and outcomes, mentioned by

For peer review only - http://bmjopen.bmj.com/site/about/guidelines.xhtml

more than one participant. For an even more comprehensive table including all CEMOs, even those mentioned by one respondent, see Table 4 (see Supplementary Material 3).

A. Practitioner—in-person—individual

Professionals confirmed that (1) *paying attention to drinking behavior* is important in interventions to reduce alcohol consumption because it could stimulate clients to start thinking about their alcohol use and create awareness of their use.

Alcohol is of course something that has a risk, can cause health damage. People think about that 'Do I want to do that to myself? I'm getting older, I'm getting more forgetful' So they start to think a lot more about what they are doing to themselves because of alcohol consumption. If you actually tell them that as a professional, then people really think twice about it: 'Wait a minute, I still want to enjoy my life and I want to age healthy'. [R9, prevention worker]

They also emphasized the importance of (2) *paying attention to lifestyle* and (3) using specific *communication approaches*, e.g. motivational interviewing. The professionals confirmed that (4) *the relationship between client and practitioner* is important because it could trigger open communication, and collaboration between client and practitioner that improves the relationship between practitioner and client. Finally, they mentioned that a (5) *favorable setting* in which the intervention is provided is important, i.e., a location that is nearby because it contributes to low-threshold participation.

B. Practitioner—not in-person—individual

Professionals confirmed that personal contact combined with feedback reduced alcohol consumption. In addition, they also mentioned several other (1) *communication approaches* as working elements, e.g. motivational interviewing, personalized contact, and anonymous participation. These approaches triggered different mechanisms, i.e., tailoring, and low-threshold and flexible participation.

And certainly with older people because they often already have certain habits for years or perhaps have a bad marriage or a very stressful job. So those life-stage issues, you can sort those out in personal treatment. [R3, psychologist and advisor]

It also makes it low threshold for people to call. We often have people on the phone who admit for the first time at all that something is going on. […] For example, people often call in their car and I think that is also a nice anonymous place where no one, no family member, a colleague, can see who they

For peer review only - http://bmjopen.bmj.com/site/about/guidelines.xhtml

are calling. […] People can choose when and where they have this conversation, so they can choose a time and location that they prefer.[R4, health care worker]

The professionals added that (2) *the relationship between client and practitioner* is important, e.g. when the professional is empathic, supportive and listens to the client because this could trigger engagement followed by open communication where the client accepts advice or information from the practitioner. Finally, they also mentioned that (3) *providing additional help* can be important because it could provide extra attention and tailoring.

C. Practitioner—in-person—relatives

Professionals confirmed that a partner is more understanding and supportive for the client when (1) *the partner is taught to deal with the drinking behavior of the client*, and that (2) *support of relatives* can lead to alcohol reduction among clients.

It is important for them to understand how addiction works, that it's not solved at once and that it is actually a disease. […] . So, the relative regains a bit more confidence and yes, this often creates space in which they can grow together and take steps. On the one hand, this strengthens the client and, on the other hand, it gives a relative more confidence that it can be done. [R16, peer support worker]

D. Practitioner—in-person—group component

Professionals mentioned that (1) *paying attention to drinking behavior* is important because it contributes to reductions in alcohol consumption. They confirmed that (2) *paying attention to lifestyle* was also a working element in this context because it motivates people to participate in the intervention and to change, which ultimately contributes to reduced alcohol consumptions. In addition, they also mentioned that (3) *communication approaches*, including motivational interviewing, and personalized contact and conversations were important. Professionals also mentioned that (4) *the relationship between client and peers* plays an important role in interventions because it could provide recognition, connection, support, hope, safety, and new insights for the client.

There is often a lot of negativities in the family atmosphere and I think in a group with peers it is accepted that people have a problem and they start looking to get it under control. So there is engagement, an open and accepting attitude that makes people feel safe and I think that is very important in a group, that people feel very safe to talk about it without feeling judged. [R13, MH psychologist]

For peer review only - http://bmjopen.bmj.com/site/about/guidelines.xhtml

Again, (5) *the relationship between the client and practitioner*, for example when the professional has an open attitude and does not judge the client, was important in interventions according to the professionals. Providing (6) *other activities* such as eating after a joint session were helpful because they contribute to making a connection and pleasant atmosphere.

Finally, (7) a *favorable setting* was important because it contributed to low-threshold participation.

It is often a very high threshold for people to go to an addiction center. So, I think that this kind of treatment could also be given in a community entre, or a nursing home, or at the GP in a small group, or something. [R14, psychiatric mental health nurse]

E. No practitioner—not in-person—individual

Professionals mentioned that (1) *paying attention to drinking behavior* contributes to reductions in alcohol consumption. Also (2) *using tools*, e.g. an online self-help tool, was considered beneficial in this setting because it enables low-threshold participation for participants and allowed them to be actively involved. Again, (3) *the relationship between client and peers* was important, because it provided clients with togetherness, recognition and support.

*I think that for this target group it is very welcome that, additionally in an online setting, they can do it alone or with a friend, or with a group where you meet once per week or two weeks. [*RP6, founder of intervention and manager]

Also, (4) *a favorable setting* was important because it could make participation low-threshold. Finally, (5) *additional help*, i.e., providing personalized content and help additional to self-help, was also seen as effective.

F. No practitioner—not in-person—group component

Professionals mentioned that (1) *paying attention to drinking behavior*, (2) *self-help groups* and (3) *the relationship between the client and peers* were all important in interventions in this context. No mechanisms were found that were mentioned by more than one professional.

For peer review only - http://bmjopen.bmj.com/site/about/guidelines.xhtml

Table 3. Summary of the program theory

Context	Element of intervention	Mechanism	Outcome	Target group interventio n**	IPT confirmed ***
**A. Practitioner – in-person – individual**	1. *Paying attention to drinking behavior*				
	1.1. Paying attention to drinking behavior	Think and act differently about alcohol consumptions	Less or no alcohol consumption		✓
	1.2. Tracking alcohol consumption and reflecting on consumption	Creating insight into consumption<br>Thinking about alcohol consumption	Less or no alcohol consumption<br>Awareness	II, III, IV, V	+
	1.3. Paying attention to abstinence, coping planning and practicing with abstinence	Raising awareness alcohol consumption		I, III, IV, X	+
	1.4. Pointing out the risks and consequences of drinking behavior	Thinking about indirect consequences of consumption	Awareness alcohol consumption, less or no alcohol consumption	I, II, IV	+
	2. *Paying attention to lifestyle*				
	2.1. Paying attention to meaning of life, and teaching lifestyle changes		Less or no alcohol consumption	II	+
	3. *Communication approaches*				
	3.1. Motivational interviewing			II, X	+
	3.2. Personalized content and conversation			I, II	+
	4. *The relationship between the client and practitioner*				
	4.1. Safety, trust, connection, and not being judged in contact with professional	Having open communication	Less alcohol consumption	II	+
	4.2. Empathic behavior of practitioner	Collaborating in the identification of needs and help, and improving the relationship between the client and the practitioner	Less or no alcohol consumption		✓
	4.3. Critical, controlling and confronting behavior of practitioner			II, V, IV, X	+
	5. *Favorable setting*				
	5.1. Location is nearby home and/or familiar	Low-threshold participation		I, IV	+
**B. Practitioner – not in person – individual**	1. *Communication approaches*				
	1.1. Contact always possible	Accessible help in difficult situations		I, III	+
	1.2. Conversation over the phone	Accessible and flexible participation		I, X	+
	1.3. Anonymous	Distance between practitioner and client<br>Client is more likely to accept advice		I	+
		Low-threshold		I	+
		Honest instead of socially desirable contact		I	+

				Think differently about alcohol consumption	I	+
	1.4. Motivational interviewing			Awareness	I, X	+
	1.5. Personalized content and conversation	Tailoring			I, III	+
		Non-commitment disappears		Less or no alcohol consumption	I, III	+
		Emotional support			I, III, X	+
	1.6. Personal contact and feedback			Less or no alcohol consumption		✓
	1.7. * Online communication and feedback			Less or no alcohol consumption		-
	2. *The relationship between the client and practitioner*					
	2.1. Practitioner is empathic, supportive and listens	Engagement	Open communication and client accepts advice or information from practitioner		I	+
	3. *Providing additional help*					
	3.1. Personal treatment with practitioner additional to selfhelp	Extra attention, tailoring			III, X	+
**C . Practitioner—in-person—relatives**	1. *Teaching the partner to deal with drinking behavior*					
	1.1. Teaching the partner to deal with drinking behavior	More understanding and support from the relative for the client			IV	✓
	2. *Support of relatives*					
	2.1. Relative provides support			Less or no alcohol consumption	X	+
**D. Practitioner—in-person—group component**	1. *Paying attention to drinking behavior*					
	1.1. Pointing out the risks and consequences of drinking behavior				IV, V	+
	1.2. Paying attention to abstinence and practicing with abstinence			Less or no alcohol consumption	IV	+
	2. *Paying attention to lifestyle*					
	2.1. *Motivating to change lifestyle					~
	2.2. Attention and tips regarding meaning of life, lifestyle and problems when aging	Motivation for participation and change		Less or no alcohol consumption, know how to improve quality of life during abstinence	IV, X	+
	2.3. *Motivating to change lifestyle delivered in a workplace setting					~
	2.4. In a workplace setting and paying attention to prevention and lifestyle.				X	+
	3. *Communication approaches*					
	3.1. Motivational interviewing			Less or no alcohol consumption, awareness	IV, X	+
	3.2. Personalized content and conversation				IV, X	+
	4. *The relationship between the client and peers*					
	4.1. Contact with peers	Recognition, connection and support			IV, V, X	+
	4.2. Closed group and maximized group size	Create an emotionally safe space			IV	+
		More attention per client				

For peer review only - http://bmjopen.bmj.com/site/about/guidelines.xhtml

	4.3. Engagement, understanding and support towards peers	Togetherness, connection and safety	Less or no alcohol consumption	IV	+
	4.4. Addressing peers	Gain new insights	Less or no alcohol consumption	II, III, IV, V	+
	4.5. Sharing experiences and tips with peers	Being hopeful in own process<br>Feeling recognized, relativating own situation		IV, V, X	+
	5. *The relationship between the client and practitioner*				
	5.1. Open attitude, not being judged and accessible contact with professional			I, V	+
	6. *Other activities*				
	6.1. Having lunch after every session	Make a connection and pleasant atmosphere		IV, V	+
	7. *Favorable setting*				
	7.1. Pleasant and relaxed atmosphere			I, IV	+
		Increase awareness		X	+
	7.2. Location is nearby home and/or familiar	Low-threshold participation		I, IV	+
**E: No practitioner — not in-person— individual**	1. *Paying attention to drinking behavior*				
	1.1. Tracking alcohol consumption	Insight into consumption and keep track of progress	Less or no alcohol consumption	III, X	+
	1.2. Abruptly quit alcohol use	Positive learning experience		II, III	+
	1.3. Pointing out the risks and consequences of drinking behavior			III	+
	2. *Using tools*				
	2.1. *Web based interventions				~
	2.2. Online self-help tool	Accessible, low-threshold and controlled participation	Awareness, less or no alcohol consumption, going to treatment	III, X	+
	2.3. Via telephone			X	+
	2.4. Regular newsletter	Being actively engaged		III	+
	3. *The relationship between the client and peers*				
	3.1. Joining with others, contact with peers	Togetherness, recognition and support		III	+
	4. *Favorable setting*				
	4.1. Location independent participation	Accessible and low-threshold		III	+
	5. *Additional help*				
	5.1. Personalized content and help additional to self-help			III	+
**F: No practitioner – not in-person - individual**	1. *Paying attention to drinking behavior*				
	1.1.*Intervention to abstinent people				~
	1.2. Focus on abstinence and paying attention to withdrawal			X	+
	2. *Self-help groups*				
	2.1. AA en self-help group			II, X	+
	3. *The relationship between the client and peers*				
	3.1.Contact with peers			III, IV, X	+
	3.2. Sharing experiences and tips with peers			IV,	+

For peer review only - http://bmjopen.bmj.com/site/about/guidelines.xhtml

*= Elements of Initial program theory that were refined, or were not confirmed.

** I = People with all types of alcohol use patterns, II = People with early-stage problematic alcohol use; III = People with all types of alcohol use patterns, except for (heavy) problematic alcohol use or alcohol addiction; IV = People with problematic alcohol use and alcohol addiction; V = People who are in recovery from addiction, X = No target group mentioned because these elements were derived from CEMO configuration that we presented as statements during the interviews.

***= ✓ = Initial theory confirmed, ~ = Initial theory refined, + = New theory added − = Initial theory not confirmed.

For peer review only

DISCUSSION

Key findings

Overall, five elements that were mentioned by more than one respondent were found in minimally three contexts and can be labelled as general working elements: 1) *pointing out the risks and consequences of drinking behavior*, 2) *paying attention to abstinence*, 3) *promoting contact with peers*, 4) providing *personalized content* and 5) *providing support*. We also found context-specific working elements, namely *providing personalized conversations* and *motivational interviewing,* specifically in interventions with practitioner involvement; *ensuring safety, trust and a sense of connection* and *a location nearby home or a location that people are familiar with* in in-person interventions; and *sharing experiences and tips* in interventions in group settings. Moreover, two mechanisms that were mentioned by more than one respondent were found in minimal in three contexts: *awareness* and *low-threshold participation/accessibility*. Our findings are generally in line with the initially formed program theory. However, we found many additional working elements, mechanisms and outcomes that were posited by several professionals resulting in a refined program theory.

Interpretation of findings

The first addition to the IPT is that older adults need social contact and support. *Promoting contact with peers* can help reduce loneliness or social isolation, and *providing support* from a practitioner, peers or relatives can help them to deal with negative emotions and problematic alcohol use. Compared to the general adult population, older adults are facing more often retirement, illness, loss of relatives, loneliness, or hopelessness, which may facilitate negative health consequences of alcohol consumption (26, 27). It is important to facilitate the process of helping older adults to develop contacts, for example in leisure settings *on a location that is nearby home and/or familiar* to make participation low-threshold.

The second addition to our IPT is that not only personalized feedback, but also *providing personalized content and conversations* are helpful for older adults, which is in line with findings in previous research showing that a personal approach with personalized feedback (21-23) and personalized reports (21, 22) are effective for older adults. Understanding the client’s motives for alcohol use can help providers to be more specific and effective for providing personalized and tailored interventions. A tailored approach with consideration of the client’s needs with aging-related challenges taken into account appears to be very effective(28) .

For peer review only - http://bmjopen.bmj.com/site/about/guidelines.xhtml

The third addition to our IPT is that both *the relationship between client and the practitioner* and *the relationship between client and peers* are important in interventions. Previous research showed that therapeutic alliance in the relationship between client and practitioner is associated with a reduced likelihood of dropout (29). Furthermore, older adults with substance use problems prefer interventions that are led by warm, honest, caring and relatively non-confrontational providers (30). These findings are in line with our working element *ensuring safety, trust and a sense of connection* and the importance of *the relationship between client and provider*.

In contrast, previous research showed mixed findings on the relationship between client and peers in substance use disorder populations: some research has found a significant association between group cohesion and intervention outcomes, and group processes and outcomes (31-36), while other research has found no significant association between group cohesion and outcomes (37, 38). Despite the fact that there seems to be no consensus in literature in the association between the relationship between client and peers, and intervention outcomes for the general adult population, we argue that there could be a strong association for older adults. Age-specific treatments may work better with older adults than mixed-age treatments(39). A group composition that match the preferences of older adults could be a pre-requisite to *ensure safety, trust and a sense of connection*, which contribute to positive outcomes.

Finally, we found two important mechanisms that contribute to positive interventions outcomes. First, awareness is an important mechanism in preventing or reducing problematic alcohol use. A previous study by Veerbeek and colleagues (2017) reported that older adults have limited awareness of the negative health effects of alcohol, and that the majority of older adults in their study did not think alcohol is addictive or unhealthy. In addition, the current standard for responsible alcohol consumption is often known to only a small proportion among older adults (40), which could suggest that only a few older adults recognize their own drinking pattern as problematic. This lack of knowledge emphasizes the importance of the working element *pointing out the risks and consequences of drinking behavior among* older adults. Emphasizing these risks can be experienced as threatening and therefore it is important to enhance older adults self-efficacy in the intervention first (41). Furthermore, knowledge is a fundamental first step, but information provision alone is not sufficient for behavior change (42). Second, accessibility/low-threshold is important in interventions for older adults. A major barrier for older adults seeking help is that they do not want to be labelled an ‘alcoholic’ or ‘addict’ (40), which can

For peer review only - http://bmjopen.bmj.com/site/about/guidelines.xhtml

make participation in interventions that are provided in addiction care centres high threshold. Providing interventions on a location that people do not directly associate with alcoholism, e.g. a community center, can make participation low-threshold. Another barrier for older adults can be difficulties with transportation (30). Travel allowance and accessibility of interventions can help older adults overcome these barriers.

Limitations and strengths

This study has some limitations. In the Netherlands, people with lower socio-economic status and ethnic minorities are often not reached by lifestyle intervention efforts and show low participation rates (43, 44). It is possible that the included interventions in this study are less likely to reach these groups, which could mean are that the professionals have limited experiences with the subgroups and that the overview of working elements and mechanisms in not fully adequate for these subgroups. It is therefore important to provide interventions with *personalized content and conversations* for specific subgroups, because it can provide a tailored approach. For example, older adults with a language deficiency could benefit from more specific elements in communication with the professional. Also, cultural differences may ask for different approaches. A study showed that religious older Moroccan-Dutch and Turkish-Dutch women can have other motivators (e.g. religious or cultural) to abstain from alcohol consumption than older native Dutch women had (45). Further research should focus on what works for specific subgroups in older adults and to what extent these older adults participate in interventions to prevent or reduce problematic alcohol use. The second limitation concerns biases when the CEMO statements were presented at the end of the interview. Possibly acquiescence bias occurred here, meaning that respondents agreed more easily to these statements, e.g. because they responded in ways they thought the researcher wanted them to respond, or because they wanted to finish the interview. Respondents were invited to explain their opinions on these statements, but they often responded by only saying that they agreed to these statements without explanation. Habituation bias could have occurred as well when the participants provided the same answers in response to similarly-worded presented CEMO statements. These two biases may influence our confirmed IPT. Finally, some CEMO configurations were only confirmed when the statements were presented. Some working elements, e.g. empathic behavior of the practitioner, might be so obvious that they were therefore initially not mentioned. We think that there could be more of such elements that are missing in our overview because of these reasons.

For peer review only

A strength of this study is that we were able to include a variety of professionals across all eleven interventions, and the high response rate. Another strength is the Realist Evaluation approach was used, as this provides explanations from the perspective of professionals in what works to prevent or reduce problematic alcohol use among older adults. This approach provides in-dept insights in interventions because it can explain why interventions are successful or not.

Practical and research implications

Based on our findings, we have a number of recommendations for practice and future research. Professionals and developers of interventions might focus on three aspects. First, practitioners should have attention for the elements that are related to the role of the practitioner in in-person interventions. Second, developers of interventions need to pay particular attention to accessible or low-threshold participation, for example by providing interventions in a *favorable setting*. Third, practitioners and developers together should focus more on the element *promoting contact with peers*. For example, practitioners working in group interventions can invite clients to *share experiences and tips* with peers, and developers of online self-help tools can refer people to online forums where people can *share experiences and tips* with peers.

Further research should focus on perspectives of clients who participate in interventions and their relatives, to refine the program theory. Also, further research should focus on interventions in the context with practitioner, not in-person, and individual and in context with no practitioner, not in-person, and in groups is warranted, because elements in these contexts are limited in this study.

CONCLUSION

In addition to the previous formulated IPT, our findings emphasize the need for social contact and support, personalized content, and strong relations (both between client and practitioner, and client and peers) in interventions for older adults. Whereas the working element 'social contact' was not found in previous literature as a working element, probably due to limited performed studies, our study found this as an additional important working element, and providers of alcohol interventions should be aware and make use of this element when treating older adults. Future research should study perspective of older adults themselves and their relatives on working elements and mechanisms in interventions.

REFERENCES

For peer review only - http://bmjopen.bmj.com/site/about/guidelines.xhtml

1. Breslow RA, Castle IJP, Chen CM, Graubard BI. Trends in Alcohol Consumption Among Older Americans: National Health Interview Surveys, 1997 to 2014. Alcoholism: Clinical and Experimental Research. 2017;41(5):976-86.
2. Han BH, Moore AA, Sherman S, Keyes KM, Palamar JJ. Demographic trends of binge alcohol use and alcohol use disorders among older adults in the United States, 2005–2014. Drug and Alcohol Dependence. 2017;170:198-207.
3. Kim JW, Lee DY, Lee BC, Jung MH, Kim H, Choi YS, et al. Alcohol and Cognition in the Elderly: A Review. Psychiatry Investigation. 2012;9(1):8.
4. Roche AM, Kostadinov V. Baby boomers and booze: we should be worried about how older Australians are drinking. Medical Journal of Australia. 2019;210(1):38-9.
5. Wolf I-K, Du Y, Knopf H. Changes in prevalence of psychotropic drug use and alcohol consumption among the elderly in Germany: results of two National Health Interview and Examination Surveys 1997-99 and 2008-11. BMC Psychiatry. 2017;17(1).
6. Mauro PM, Askari MS, Han BH. Gender differences in any alcohol screening and discussions with providers among older adults in the United States, 2015 to 2019. Alcoholism: Clinical and Experimental Research. 2021;45(9):1812-20.
7. Grant BF, Chou SP, Saha TD, Pickering RP, Kerridge BT, Ruan WJ, et al. Prevalence of 12-Month Alcohol Use, High-Risk Drinking, and <i>DSM-IV</i> Alcohol Use Disorder in the United States, 2001-2002 to 2012-2013. JAMA Psychiatry. 2017;74(9):911.
8. Knott CS, Scholes S, Shelton NJ. Could more than three million older people in England be at risk of alcohol-related harm? A cross-sectional analysis of proposed age-specific drinking limits. Age Ageing. 2013;42(5):598.
9. Meier P, Seitz HK. Age, alcohol metabolism and liver disease. Current Opinion in Clinical Nutrition and Metabolic Care. 2008;11(1):21.
10. Cederbaum AI. Alcohol Metabolism. Clinics in Liver Disease. 2012;16(4):667-85.
11. Moore AA, Whiteman EJ, Ward KT. Risks of combined alcohol/medication use in older adults. The American Journal of Geriatric Pharmacotherapy. 2007;5(1):64-74.
12. Kuerbis A, Sacco P, Blazer DG, Moore AA. Substance abuse among older adults. Clinics in geriatric medicine. 2014;30(3):629-54.
13. Schonfeld L, Dupree LW. Treatment Approaches for Older Problem Drinkers. International Journal of the Addictions. 1995;30(13-14):1819.
14. Dupree LW, Broskowski H, Schonfeld L. The Gerontology Alcohol Project: A Behavioral Treatment Program for Elderly Alcohol Abusers. The Gerontologist. 1984;24(5):510.
15. Rice C, Longabaugh R, Beattie M, Noel N. Age group differences in response to treatment for problematic alcohol use. Addiction. 1993;88(10):1369-75.
16. Seddon JL, Wadd S, Wells E, Elliott L, Madoc-Jones I, Breslin J. Drink wise, age well; reducing alcohol related harm among people over 50: a study protocol. BMC Public Health. 2019;19(1).
17. Schultz SK, Arndt S, Liesveld J. Locations of facilities with special programs for older substance abuse clients in the US. International Journal of Geriatric Psychiatry. 2003;18(9):839-43.
18. Armstrong-Moore R, Haighton C, Davinson N, Ling J. Interventions to reduce the negative effects of alcohol consumption in older adults: a systematic review. BMC Public Health. 2018;18:13.
19. Megherbi-Moulay O, Igier V, Julian B, Franchitto N, Sordes F. Alcohol Use in Older Adults: A Systematic Review of Biopsychosocial Factors, Screening Tools, and Treatment Options. International Journal of Mental Health and Addiction. 2022.

For peer review only - http://bmjopen.bmj.com/site/about/guidelines.xhtml

20. Boumans J, van de Mheen D, Crutzen R, Dupont H, Bovens R, Rozema A. Understanding How and Why Alcohol Interventions Prevent and Reduce Problematic Alcohol Consumption among Older Adults: A Systematic Review. Int J Environ Res Public Health. 2022;19(6):3188.
21. Ettner SL, Xu H, Duru OK, Ang A, Tseng C-H, Tallen L, et al. The Effect of an Educational Intervention on Alcohol Consumption, At-Risk Drinking, and Health Care Utilization in Older Adults: The Project SHARE Study. Journal of Studies on Alcohol and Drugs. 2014;75(3):447-57.
22. Fink A, Elliott MN, Tsai M, Beck JC. An Evaluation of an Intervention to Assist Primary Care Physicians in Screening and Educating Older Patients Who Use Alcohol. Journal of the American Geriatrics Society. 2005;53(11):1937-43.
23. Kuerbis AN, Yuan SE, Borok J, Lefevre PM, Kim GS, Lum D, et al. Testing the Initial Efficacy of a Mailed Screening and Brief Feedback Intervention to Reduce At-Risk Drinking in Middle-Aged and Older Adults: The Comorbidity Alcohol Risk Evaluation Study. Journal of the American Geriatrics Society. 2015;63(2):321-6.
24. Pawson R, Tilley N, Tilley N. Realistic Evaluation: SAGE Publications; 1997.
25. Tong A, Sainsbury P, Craig J. Consolidated criteria for reporting qualitative research (COREQ): a 32-item checklist for interviews and focus groups. International Journal for Quality in Health Care. 2007;19(6):349.
26. Kuerbis A. The impact of retirement on the drinking patterns of older adults: a review. Addictive Behaviors. 2012;37(5):587.
27. Barry KL. Drinking over the lifespan: Focus on older adults. Alcohol Research: Current Reviews. 2016;38(1):115.
28. Atiq Rafay R. Common themes and issues in geriatric psychotherapy. Psychiatry (Edgmont (Pa : Township)). 2006;3(6):53-6.
29. Meier PS, Donmall MC, McElduff P, Barrowclough C, Heller RF. The role of the early therapeutic alliance in predicting drug treatment dropout. Drug and Alcohol Dependence. 2006;83(1):57-64.
30. Holland JM, Rozalski V, Beckman L, Rakhkovskaya LM, Klingspon KL, Donohue B, et al. Treatment Preferences of Older Adults with Substance Use Problems. Clinical Gerontologist. 2016;39(1):15-24.
31. Valeri L. Group therapy for women with substance use disorders: In-session affiliation predicts women's substance use treatment outcomes. Journal of Substance Abuse Treatment. 2018;94:60.
32. Kelly PJ, Deane FP, Baker AL. Group Cohesion and Between Session Homework Activities Predict Self-Reported Cognitive–Behavioral Skill Use Amongst Participants of SMART Recovery Groups. Journal of Substance Abuse Treatment. 2015;51:53-8.
33. Cavicchioli M. Group relationships during a dialectical behavior therapy skills training program for the treatment of alcohol and concurrent substance use disorders: Evidence and theoretical considerations. Group Dynamics: Theory, Research, and Practice. 2021;25(2):152.
34. Sotskova A. Understanding the role of group cohesion and group alliance in a secular peer support group for recovery from substance misuse. Journal of Groups in Addiction and Recovery. 2016;11(2):137.
35. Rice SL, Tonigan JS. Impressions of Alcoholics Anonymous (AA) Group Cohesion: A Case for a Nonspecific Factor Predicting Later AA Attendance. Alcoholism Treatment Quarterly. 2012;30(1):40-51.
36. Pooler DK, Qualls N, Rogers R, Johnston D. An Exploration of Cohesion and Recovery Outcomes in Addiction Treatment Groups. Social Work with Groups. 2014;37(4):314-30.
37. Cruz M, Osilla KC, Paddock SM. Group Cohesion and Climate in Cognitive Behavioral Therapy for Individuals with a First-Time DUI. Alcoholism Treatment Quarterly. 2020;38(1):68-86.
38. Gillaspy JA, Wright AR, Campbell C, Stokes S, Adinoff B. Group Alliance and Cohesion as Predictors of Drug and Alcohol Abuse Treatment Outcomes. Psychotherapy Research. 2002;12(2):213-29.

For peer review only - http://bmjopen.bmj.com/site/about/guidelines.xhtml

39. Kuerbis A, Sacco P. A Review of Existing Treatments for Substance abuse among the Elderly and Recommendations for Future Directions. Substance Abuse: Research and Treatment. 2013;7:SART.S7865.
40. Veerbeek M, Heijkants C, Willemse B. Alcoholgebruik onder 55-plussers. Utrecht: Trimbos Instituut. 2017.
41. Peters G-JY, Ruiter RAC, Kok G. Threatening communication: a critical re-analysis and a revised meta-analytic test of fear appeal theory. Health Psychology Review. 2013;7(sup1):S8-S31.
42. Arlinghaus KR, Johnston CA. Advocating for Behavior Change With Education. American Journal of Lifestyle Medicine. 2018;12(2):113-6.
43. El Fakiri F, Hoes AW, Uitewaal PJM, Frenken RAA, Bruijnzeels MA. Process evaluation of an intensified preventive intervention to reduce cardiovascular risk in general practices in deprived neighbourhoods. European Journal of Cardiovascular Nursing. 2008;7(4):296.
44. Magnée T, Burdorf A, Brug J, Kremers SPM, Oenema A, Van Assema P, et al. Equity-Specific Effects of 26 Dutch Obesity-Related Lifestyle Interventions. American Journal of Preventive Medicine. 2013;44(6):e61-e70.
45. Sialino LD, Wijnhoven HAH, Van Oostrom SH, Picavet HSJ, Verschuren WMM, Visser M, et al. Perspectives of older women in the Netherlands: identifying motivators and barriers for healthy lifestyles and determinants of healthy aging. BMC Public Health. 2023;23(1).

Data availability statement

No additional data available.

Competing interests

The authors declare no conflict of interest.

Funding

This research was funded by The Netherlands organisation for Health Research and Development (ZonMw number: 555002034).

For peer review only

For peer review only - http://bmjopen.bmj.com/site/about/guidelines.xhtml

Table 2. Intervention characteristiscs

Intervention	Contact in intervention with			Setting			Sessions			Target group intervention	
	Professional	Peers	Relatives	Online, telephone	In-person	Anonymous	1	>1	N/a	Alcohol use**	Older adults (55+ or 65+)
Alcohol Information line	X			X		X	X	X*		I	
CBT for substance use	X	X	X*		X			X		IV	X
Drink Less	X*			X		X*			X	III	
Enjoy Healthy	X	X			X		X			I	X
Fresh Onwards	X	X	X		X			X		IV	X
Maxx				X		X			X	III	
Mirro				X		X			X	III	
Moti-55	X		X*		X			X		II	X
NoThanks	X*			X		X*	X*	X*	X	III	
Recovery guided personal program	X		X*		X			X		V	
Vitality days	X	X*	X*		X		X			I	X

* = Optional, depending on client's preference

** I = People with all types of alcohol use patterns, II = People with early stage problematic alcohol use; III = People with all types of alcohol use patterns, except for (heavy) problematic alcohol use or alcohol addiction; IV = People with problematic alcohol use and alcohol addiction; V = People who are in recovery from addiction

For peer review only

Supplementary Material 2: Interviewguide

Background information intervention

- What is the aim of [name intervention]?

- Is there a specific strategy, method or approach used in the interventions? Can you explain?
- Which target group/people can participate in [name intervention]? Why?
- Which target groups/people can nog participate in [name intervention]? Why?

[DEPENDING ON PRACTITIONER INVOLVEMENT OR NOT]

- What is the role of the professional?
- What does the contact between participant and professional look like?
- What expertise, or qualifications, must a professional have to provide [name intervention]? And why?

I am now going to ask you questions about your perspective on the working elements of [name intervention]and how it is provided. I would like to ask you to answer from your <u>own experience</u> as a professional.

Working elements

- What impact do you think [name intervention] has on the <u>actual alcohol</u> <u>consumption</u> of participants aged 55+?
- What impact do you think [name intervention] has on how people aged 55+ <u>think about alcohol consumption?</u>

- In your opinion, what makes [name of intervention] cause [listed outcomes]?
- What do you think are working elements of the intervention?
  For each element, ask the following things:
  o What works well for whom? Can you explain this?
  o To what extent do you think this works well? Can you explain this?
  o How does this work? Can you explain?
  o Why do you think this works well? Can you explain?

Context

- What is the impact of how [the support/name of intervention] is provided? By provided you can think of it being offered [individually/in a group] and [online/offline]?
  o For whom do you think this mode works well? Can you explain this?
  o To what extent do you think this works well? Can you explain?
  o Why does this work well according to you? Can you explain?
  o Why do you think this works well? Can you explain?

- What is the impact of the location where [the support/name of intervention] is provided?
  o For whom do you think this location works well? Can you explain?
  o To what extent do you think this works well? Can you explain?
  o Why does it works well? Can you explain?
  o Why do you think this works well? Can you explain?

Relatives

- To what extent do participants aged 55+ receive support from relatives during [name intervention]?
- o What does this support look like?
- What is the impact of support from relatives? Can you explain?
  o For whom do you think support works well? Can you explain?
  o To what extent do you think this support works well? Can you explain?
  o Why do you think it works well? Can you explain?
  o Why do you think this works well? Can you explain?

- What support would (further) be desirable during participation in [name intervention]? How do you think this could be achieved?

- How do you think relatives experience providing support?
- To what extent do you think relatives need help or support?

CEMO statements

I am going to present a few statements. I would like to invite you to respond to these statements.
Practitioner – in-person – individual (A)<br>• An intervention with face to face guidance from a professional can make people (55+) reduce or stop drinking.<br>o Definitely when there is a focus on drinking bheaviour, making people think about their own alcohol use.<br>o Definitely when the professional is very empathetic, making participant and professional work together in finding appropriate help and improving their relationship.
Practitioner – not in-person – individual (B)<br>• An intervention with no face-to-face guidance from a professional can make people (55+) reduce or stop drinking.<br>o Definitely when there is personal contact with feedback.<br>o Definitely when there is online communication with feedback.
Practitioner – in-person – relatives (C)<br>• An intervention with face to face guidance from a professional with relatives' involvement can make people (55+) reduce or stop drinking.<br>o Defenitely when the partner is taught to deal with the behavior of the drinking partner, this can lead to more understanding and support from the non-drinking partner for the drinking partner.
Practitioner – in-person – group component (D)<br>• An intervention with face-to-face guidance from a professional in a group setting can make people (55+) reduce or stop drinking.<br>o Definitely when people are motivated to make lifestyle changes.<br>o Definitely when this is delivered in a workplace setting.
No practitioner – not in-person – individual (E)<br>• An intervention without guidance from a professional can make people (55+) reduce or stop drinking. .<br>o Definitely in web based interventions<br>o Definitely with telephone based interventions.
No practitioner – not in-person – group component (F)<br>• An intervention without guidance from a professional in a group setting can make people (55+) reduce or stop drinking.<br>o Definitely when the intervention focuses on abstinention, which increases awareness of their alcohol consumption
Closing interview *(5 minutes)*
• We are including the following interventions in the study: Moti-55, Ikpas, the alcohol info line, Fris Verder, Healthy Enjoyment, Vitality Days, Maxx, the LessDrinking app and Mirro. To your knowledge, are there any other interventions for older adults (55+)?
• Is there anything else you wild like to add to the interview?
• We would like to publicly share the data and information from these interviews and questionnaire you have completed with other researchers. This will allow this information to be reused for new research in the future.<br>• Before we share this data, we will anonymise all information. This means that personal data or data that can be traced back to you as a person such as name, age or other info that reveals your identity will not be shared with them. Such information will be blacked out before the data is made public.<br>• Do you give permission to store the anonymised research data for an unlimited period and to make it publicly available to other researchers?<br>You are not required to give permission for this. Without permission, we will not make the data publicly available and it will not be used for follow-up research. Even if you do not give permission for this, your data can still be used for current research.
**Recorder off**
• I would like to thank you for the interview and the time you were willing to spend.<br>• You will be digitally sent a voucher worth 20 euros.

- Should there be any questions at a later moment, please contact me.
- Also, if you do not have any questions but feel the need to talk after, please contact me.

For peer review only

Supplementary Material 3. Comprehensive program theory.

Context	Element of intervention (How)	Mechanism (Why)	Outcome
**A.Practitioner – in-person – individual**	(No element, context only)	No misery others (II)<br>Practitioner functions as a big stick (II)<br>Dare to discuss things face to face better than online (IV)	
	1. Paying attention to drinking behavior		
	1.1 Paying attention to drinking behavior		
	1.1.1. Paying attention to drinking behavior	Thinking and awareness	Less or no alcohol consumption (X)
	1.1.2. Conversation with practitioner about alcohol consumption (X)		
	1.2 Tracking alcohol consumption and reflecting on consumption		
	1.2.1. Reflecting on alcohol consumption (II, X)	Insight to consumption (II)<br>Confrontation and reflection (II)	
	1.2.2. Tracking alcohol consumption (II)	Insight to consumption (II, III, IV)<br>Confrontation (II)	
	1.2.3. Paying attention to the cause of alcohol consumption (X)	-<br>Thinking about consumption/lifestyle (II)	Less or no alcohol consumption (X)<br>Awareness and insight alcohol consumption (II)
	1.3. Paying attention to abstinence, coping planning and practicing with abstinence		
	1.3.1. Coping planning abstinence (I, IV, X)		
	1.3.2. Practicing with abstinence	-	Awareness alcohol consumption (IV)
	1.3.3. Raising awareness of responsible consumption/abstinence (I)	Awareness alcohol consumption (I)	
	1.4. Pointing the risks and consequences of drinking behavior		
	1.4.1. Information about risks and consequences (I, II, IV, X)	Thinking about and recognize indirect consequences in own vulnerability (I, II)	Awareness of effects of alcohol consumption, less or no consumption (I, II)
		Shocked by consequences	Awareness of health effects of alcohol consumption (I)
		Insight to consumption (IV)	
		Increase risks perception for consequences → Motivation to reduce	Less alcohol consumption (IV)
	2.Paying attention to lifestyle		
	1.1. Paying attention and giving tips of meaning of life, and teaching lifestyle changes		
	2.1.1. Attention and tips of meaning of life	Meaning in life	Less or no alcohol consumption (II)
	2.1.2. Teaching lifestyle changes (II)		
	2.1.3. Attention to different aspects of life (II)		
	3. Communication approaches		
	3.1. Motivational interviewing		
	3.1.1 Motivational interviewing (X, II)	Connect with client (X)	
		-	Awareness (X)
	3.2. Personalized content and conversation		

3.2.1. Personalized content and conversation (I, II)			
4. The relationship between the client and practitioner			
4.1. Safety, trust, connection, open attitude and not being judged in contact with the practitioner			
4.1.1. Client feels comfortable with practitioner (II)			
4.1.2. Low-threshold contact with practitioner (X)	-		Less or no alcohol consumption
4.1.3. Contact with practitioner feels safe	Opening conversation to discuss drinking behavior (X)		
4.1.4. Client feels accepted	Opening conversation to discuss drinking behavior (X)		
4.1.5. Client feels connection with practitioner	Opening conversation to discuss drinking behavior (X)		
4.1.6. Practitioner has an open attitude and does not judge	Client feels comfortable<br>-		Opening up at follow-up conversation (II)<br>Moderate drinking/awareness (II)
4.1.7. Good therapeutic relationship (X)			
4.1.8. Practitioner is friendly	Client feels comfortable		Openness at follow-up conversation (II)
4.2. Emphatic behavior of practitioner			
4.2.1. Practitioner is empathic (I, X)	Client feels understood about alcohol-problems (X)	Client and practitioner collaborate in the identification of additional help, and the relationship between the client and the therapist improves	Less or no alcohol consumption (X)
	Client and practitioner collaborate in the identification of additional help, and the relationship between the client and the therapist improves (X)		
	Good relationship of trust (X)		
	Client becomes more honest and willing to discuss things (X)		
4.3. Critical, controlling and confronting behavior of practitioner			
4.3.1. Practitioner has active role/guidance (II)	Engagement (II)		
4.3.2 Practitioner makes client reflect critically on client's actions (IV)	-		Less or no alcohol consumption (X)
4.3.3 Practitioner is critical/strict (II)			
4.3.4 Practitioner sets boundaries (IV)			
4.3.5 Practitioner confronts (II)			
5. Favorable setting			
5.1. Location is nearby home and/or familiar			
5.1.1. Location is nearby home and/or familiar	Low-threshold participation (I, IV)		
	Less effort to go the location		Increase chances of participation (IV)
5.2. *Pleasant atmosphere			
5.2.1. Meeting with friendly and non-committal atmosphere*	Clients can decide for themselves what they will and will not discuss (I)		
6. *Other			

	6.1. Additional contact			
	6.1.1. Introductory interview	Alignment between practitioner and client		Client wants follow-up interview (II)
	6.1.2. Multiple sessions	More attention for (different) themes and needs (II)		
	6.1.3. Conversation with general practice-based nurse specialist/GP (II)			
	6.2. Other characteristics/input practitioner			
	6.2.1. Practitioner has much knowledge	Thinking about alcohol consumption		Motivation less alcohol consumption(X)
	6.2.2. Practitioner shares own experience	Equal contact	Connection between practitioner and client	Client is open for what practitioner has to offer (IV)
**B.Practitioner – not in-person – individual**	(No element, context only)	Practitioner functions as a big stick (X)		
	1. Communication approaches			
	1.1. Contact always possible			
	1.1.1.Always the possibility of contact (I)	Accessible help in difficult situations		Less or no alcohol consumption, think differently about alcohol consumption, being open about alcohol consumption (I, III)
	1.2. Conversation over the phone			
	1.2.1. Telephone conversation (I)	Accessible and flexible (distance, time and anonymity) (I, X)		
		Responding to emotions (I)		
	1.3. Anonymous			
	1.3.1. Anonymous and telephone conversation (I)	Distance between practitioner and client	Client is more likely to accept advice (I)	
			Client speaks and opens up easier (I)	
		Low-threshold (I)		
		Less attention, shame (I)		
		-		Think differently about alcohol (I)
		-		Being open about alcohol consumption (I)
	1.3.2. No relationship between client and practitioner	Honest instead of socially desirable contact		Think differently about alcohol consumption (I)
				Being open about alcohol consumption (I)
	1.4. Motivational interviewing			
	1.4.1. Motivational interviewing	Engagement		Awareness alcohol consumption (I, X)
		Open communication and client accept advice from practitioner (I)		
	1.5. Personalized content and conversation			
	1.5.1. Personalized content and conversation (I)	Afstemming op hulpvraag en niveau (I)		Increase knowledge and motivation (I)
		Non-commitment disappears		Less or no alcohol consumption (III)
		-		Knowledge about where to go when seeking help, learning to cope with alcohol consumption, increasing motivation for less or no alcohol consumption (I)

		Pick up emotions (I)	Establish a relationship	Communicate message better to client (I)<br>Make the client think critically (I)
		Possibility of attention other problems (III)<br>Feeling of support		Insight into the cause of the problem (III)<br>Less or no alcohol consumption (III, X)
	1.6. Personal contact and feedback			
	1.6.1. Personal contact and feedback (X)	Non-commitment disappears (X)		Less or no alcohol consumption (X)
	1.7. Other*			
	1.7.1. Five-phase model*	Open communication and clients accept advice from practitioner (I)		Awareness alcohol consumption (I)
	1.7.2. Structure, clear expectations and agreements for client* (III)	A big stick		Awareness alcohol consumption (III)
	1.7.3. Regular contact*	A big stick	Motivation	Awareness alcohol consumption, less or no alcohol consumption (III)
	2. The relationship between the client and practitioner			
	2.1. Practitioner is empathic, supportive and listens			
	2.1.1 Gentle approach with compliments (I)			
	2.1.2 Practitioner does not judge about alcohol behavior, supports and listen (I)			
	2.1.3 Practitioner support and listens (I)			
	2.1..4 Practitioner is empathic	Engagement	Clients accept information from practitioner<br>Open communication	Client will seek help from addiction services (I)
	2.2. Practitioner takes away the shame with positive approach*	Reduce craving for alcohol (I)		
	3. Providing additional help			
	3.1. Personal treatment with practitioner additional to selfhelp			
	3.1.1 Personal treatment with practitioner additional to selfhelp (III, X)	Working independent (III)<br>Keep more attention<br>Provide extra tailoring (III)<br>Possibility of involving relatives (III)<br>Recognition (III)		More likely to persist (X)
	*4. *Paying attention to drinking behavior*			
	4.1. Information about risks and possible help			
	4.1.1. Information about health risks	-		Increase knowledge about alcohol (I)
	4.1.2. Information about alcohol interventions (I)			
**C. Practitioner—in-person—relatives**	*1. Teaching the partner to deal with drinking behavior*			
	1.1 Teaching the partner to deal with drinking behavior			
	1.1.1. Teaching the partner about/dealing with drinking behavior(I, IV, X)	Partner gets more trust in client<br>More understanding and support from the relative for the client (X)		Growing together and taking steps (IV)

	2. Support of relatives			
	2.1 Support of relatives			
	2.1.1.Relatives are deployed to provide support (X)	-		Less or no alcohol consumption
	3. *Other			
	3.1. Relative controls			
	3.1.1. Relative gives or controls information (II)			
	3.2. Partner shares experience and insights with other partners			
	3.2.1 Partner shares experience and insights with other partners	Partner is relaxed and is less controlling		Easier for client to achieve goals (IV)
**D. Practitioner – in-person – group component**	(No element, context only)	Togetherness, connection, recognition (I)	Sharing 'stick behind the door' experience	Know how to improve quality of life during abstinence, abstinence (IV)
			-	Less or no alcohol consumption (IV)
		Recognition		Achieving goals (IV)
		Location gets people outside where addiction takes place (IV)		
		Safety (V)		
		Free to share things by others talking (I)		
	1. Paying attention to drinking behavior			
	1.1. Pointing out the risks and consequences of drinking behavior			
	1.1 Information about risk and consequences (IV, V)	Insight in addiction (IV)		
	1.2. Paying attention to abstinence and practicing with abstinence			
	1.2.1. Attention and tools for (sustained) abstinence	-		Less or no alcohol consumption (IV)
		Less alcohol consumption	Mood improves	People become more active and anxiety symptoms will reduce (IV)
	1.2.2. Practicing with new or tense situations	Learning to tolerate discomfort in life (IV)		
		Gain experience (IV)		
	1.3. *Discuss statements on alcohol consumption in groups*			
	1.3.1. Discuss statements in groups	Thinking about alcohol consumption (I)		
		Openness about alcohol consumption (I)		
	2. Paying attention to lifestyle			
	2.1. **Motivating to change lifestyle			
	2.2. Attention and tips regarding meaning of life, lifestyle and problems when aging			
	2.2.1 Attention for meaning of life and life stage themes (IV, X)	Motivation to go to the next session (IV)		
		Better coping with cravings for alcohol (IV)		
		Motivation for change		Abstinence, know how to improve quality of life during abstinence (IV)

2.1.2. Attention for lifestyle or motivate lifestyle change (X)	-		Less alcohol consumption (X)
2.3. **Motivating to change lifestyle delivered in a workplace setting			
2.4. In a workplace setting and paying attention to prevention and lifestyle.			
2.4.1. At work (X)	Increase awareness (X)		
2.4.2. At work with attention for prevention (X)			
2.4.3. At work with attention for lifestyle (X)			
2.4.4. At work, openness about alcohol consumption*	Negative consequences (X)<br>Support environment (X)		
	-		Less alcohol consumption (X)
2.4.5. At work, people join national campaign* (X)			
3. Communication approaches			
3.1. Motivational interviewing			
3.1.1. Motivational interviewing	-		Less or no alcohol consumption, awareness (X, IV)
3.2. Personalized content and conversation			
3.2.1. Personalized content and conversation (IV, X)			
3.2.2. Customized CBT treatment (IV)			
3.3. *Other approaches			
3.3.1. Change way of thinking from negative to positive (IV)	Rethinking and self-pity reduces	Getting stronger to prevent relapse	Less or no alcohol consumption (IV)
3.3.2. Overcoming shame			Less or no alcohol consumption (III)
3.3.3. Mindfulness and running therapy (V)	Feeling signals in body and recognize (V)		
3.3.4. CGT focusing on substance use (IV)	-		Less or no alcohol consumption
3.3.5. Structure in days	Having rhythm and purpose to go somewhere (V)		
3.3.6. Variance of working methods (IV)			
4. The relationship between the client and peers			
4.1.Contact with peers			
4.1.1. Contact with peers (IV, IV, IV, X)	Being seen and feel supported (IV)<br>Connection (IV)		
4.1.2. Group composition like-minded peers (IV, IV)	Recognition and connection (IV,V, X)		
4.2. Closed group and maximized group size			
4.2.1.Maximized group size	Creating safety<br>More attention per client (I, IV)		Preventing drop out (IV)
4.2.2.Closed group	Improves atmosphere (IV)<br>Ongoing process (IV)<br>Safety	Daring to open up (IV)	
4.3. Engagement, understanding and support towards peers			
4.3.1. Engagement and understanding of each other	Connection (IV)		

For peer review only

(IV)	Safety	Talking about problems (IV)	Less or no alcohol consumption (IV)
	Togetherness, being able to ask for help from each other (IV)		
4.3.2. Support each other (V)			
4.3.3. People feeling heard in group	Less alcohol consumption	Mood improves	People become more active and anxiety symptoms will reduce (IV)
4.4. Addressing peers			
4.4.1. Addressing each other, reflect critically on your own actions (II, V)	Learning and getting insights		Less or no alcohol consumption (III, IV)
4.5. Sharing experiences and tips with each other			
4.5.1. Sharing experiences and tips (V, X)	Hopeful for own process (IV, V)		
	Openness about alcohol consumption (IV)		
	Recognition and acknowledgement (IV)		
	Putting own situation in perspective (IV)		
	Feeling the impact of addiction (IV)		
4.6. *Making agreements with peers			
4.6.1. Making agreements among themselves in group	A big stick (IV)		
5. The relationship between the client and practitioner			
5.1. Open attitude, not being judged and accessible contact with practitioner			
5.1.1. Practitioner is unbiased, open and not moralizing (V)			
5.1.2. Accessible contact practitioner	Experience support		Less relapse in alcohol consumption (V)
5.2. Other*			
5.2.1. Practitioner has the same age of clients (IV)	Recognition and click (IV)		
5.2.2. Presence two practitioners (IV)			
5.2.3. Interactive contact	Holding attention (I)		
5.2.3. Guest speakers focussing on different lifestyle themes* (IV)	-		Abstinence, know how to improve quality of life during abstinence (IV)
6. Other activities			
6.1. After every session lunch together			
6.1.1. After every session have a lunch together (IV, V)	Connection (IV)		Less or no alcohol consumption (IV)
	Atmosphere in the group (IV)		
	Unforced atmosphere		Discuss topics in a low-threshold way that are more difficult during a session (IV)
			Safe setting (IV)
	Relaxation and getting to know each other better (IV)		
	Insight of simplicity nutritious meal (IV)		
6.2. *Activities			

	6.2.1. Social activities (V)	Daytime activities	Reduce loneliness (IV)	
	6.2.2. Outdoor intervention in group walking with app	Stimulate to move or exercise		Better coping with cravings for alcohol (IV)
	7. Favorable setting			
	7.1. Pleasant and relaxed atmosphere			
	7.1.1. Meeting with friendly and non-committal atmosphere	Clients can decide for themselves what they will and will not discuss (I)		
	7.1.2. Besides serious atmosphere, also relaxed atmosphere with humour (IV)			
	7.2. Location is nearby home and/or familiar			
	7.2.1. Location is nearby home and/or familiar	Low-threshold participation (I, IV)		
		Less effort to get to location	Increase chance of participation (IV)	
	7.3. *Location is not at admission clinic			
	7.3.1. Location is not at admission clinic	People are not haunted with memories of admission (V)		
**E. No practitioner – not in-person - individual**	*1. Paying attention to drinking behavior*			
	1.1. Tracking alcohol consumption			
	1.1.1.Monitoring alcohol consumption	Keep track of progress		Less alcohol consumption (III)
	1.1.2. Tracking alcohol consumption in app/telephone	Insight into alcohol consumption(X)		Less or no alcohol consumption (X)
	1.1.3. Information and tools relapse and dealing with difficult moments *	Prevent relapse	Handle difficult moments better and consciously (III)	
	1.2. Abruptly changing alcohol consumption			
	1.2.1. Abruptly changing alcohol consumption for a temporary period (III)	Learning experience that can be perceived as positive (II)	High self-efficacy and attitude change towards alcohol consumption (III)	High probability that people will use less or no alcohol consumption later (again) (III)
		Awareness behavioral change (III)		
	1.3. Pointing out the risks and consequences of drinking behavior			
	1.3.1. Paying attention to risks of alcohol consumption at age	Get people thinking (III)		
	1.4. *Receiving feedback			
	1.4.1. Receiving feedback	Motivation		Less alcohol consumption (III)
	1.4.2. Positive feedback	Motivation		Less alcohol consumption (III)
	2. Using tools			
	2.1. **Web based interventions			
	2.2. Online self-help tool			
	2.2.1.App	No waiting list so quick participation (III)		
		Getting started with themes in a low-threshold way (X)		
	2.2.2. App that is simple and clear	Accessible (III)		
	2.2.3. Online self-help tool /program /module	Own direction implementation (III)		Less or no alcohol consumption (X)
		Low-threshold(III)		

		-		Awareness/ Less or no alcohol consumption/ Step towards treatment (IV)
	2.2.4. Short online module with playful approach (III)	Awareness		Small changes alcohol consumption (III)
	2.3. Via telephone			
	2.3.1. Via telephone	Saving time/cost (X)		
	2.4. Regular newsletter			
	2.4.1. Regular newsletter (III)	Being actively engaged	Support (III)	
		Recognition support (III)		
		-		Being motived to use less or no alcohol consumption for longer periods of time (III)
	2.5. *Other			
	2.5.1. Webinar with peerchatbox	Personal contact what serves as social support (III)		
	3. The relationship between the client and peers			
	3.1. Joining with others, contact with peers			
	3.1.1. Additional contact with peers (III)			
	3.1.2. Joining with others (III)	Feeling of togetherness (III)		Increase success of intervention (III)
		Feeling of recognition (III)		
		Social support (III)		
	4. Favorable setting			
	4.1. Location independent participation			
	4.1.1. Location-independent/participation possible from home (III)	Accessible and low-threshold (III)		
	5. Additional help			
	5.1. Personalized content and help additional to self-help			
	5.1.1. Additional personal coaching at self-help interventions (III)			
	5.1.2. Additional physical tools for client (III)	Feeling of physical participation at home (III)		
	5.1.3. Extra options for support (III)	Feeling of personal, voluntary and non-committal participation		Less or no alcohol consumption for one month (III)
	6. *Other			
	6.1. Attention other themes			
	6.1.1. Attention to activity spending	Get people thinking (III)		
	6.1.2. Attention for difficult aspects in life	Get people thinking (III)		
	6.1.3. Commitment with yourself by signing up (III)	Motivation		Increase success of intervention (III)
	6.1.4. Non commitment participation	Low-threshold (III)		Makes less or no alcohol consumption pleasant, step up to intensive help (III)
		Extra motivation (III)		
	6.1.5. Attention to positive experiences	Increase self-efficacy (III)		
	6.1.6. Setting goals	-		Less alcohol consumption (III)
**F. No**	(No element, context only)	Motivation and not being alone		Less or no alcohol consumption (X)

**practitioner – group component**	*1. Paying attention to drinking behavior*		
	1.1. **Intervention to abstinent people		
	1.2. Focus on abstinence and paying attention to withdrawal		
	1.2.1. Focus on abstinence, with attention to withdrawal (X)		
	1.2.2. Focus on abstinence (X)		
	2. Self-help groups		
	2.1. AA and self-help group		
	2.1.1. Aa/self-help group (X)	-	Less or no alcohol consumption (II)
	3. The relationship between the client and peers		
	3.1. Contact with peers		
	3.1.1. Peer contact (IV, X)	Recognition (X)	
	3.2. Sharing experiences and tips with peers		
	3.2.1. Sharing experiences and tips with peers (III)	Hope and connection (X)<br>Persuade to participate (X)<br>Stimulate (X)	
	3.3. *Other		
	3.3.1. Composition of people with same level/serious issues (III)		
	3.3.2. Making agreements and setting goals in group	-	Less or no alcohol consumption (X)
	3.3.3. Helping each other (X)		
	3.3.4. Online forum	Social support	Temporary stop of alcohol consumption (III)
	4. *Paying attention to other aspects		
	4.1. Broader focus than abstinence		
	4.1.1. Broader focus than only abstinence	Thinking about what is important	Less or no alcohol consumption (X)

*= Program theories that were not included in Table X. ** Initial program theories that were refined or not confirmed.
I = People with all types of alcohol use patterns, II = People with early stage problematic alcohol use; III = People with all types of alcohol use patterns, except for (heavy) problematic alcohol use or alcohol addiction; IV = People with problematic alcohol use and alcohol addiction; V = People who are in recovery from addiction, X = No target group mentioned because these elements were derived from CEMO configuration that we presented as statements during the interviews.

For peer review only

Supplementary Material 1: The COREQ Checklist

**Domain 1: Research team and reflexivity**		
		Reported on page no.
Personal characteristics		
Interviewer/facilitator	Fieke van den Bulck	n/a
Credentials	MSc	Not reported in manuscript
Occupation	PhD	Not reported in manuscript
Gender	Woman	Not reported in manuscript
Experience and training	Conducted interviews as a part of her Ph.D.	Not reported in manuscript
Relationship established	yes	Not reported in manuscript
Participant knowledge of the interviewer	Created relationship during preparation for the research study .	Not reported in manuscript
Interviewer characteristics		Not reported
**Domain 2: Study design**		
Methodological orientation	Interviews with a realist evaluation approach	p. 5, 8
Sampling	Purposive and convenience sample	Not reported in manuscript
Method of approach	Email or telephone	p. 5, 6
Sample size	20 interviews with health care worker, prevention worker, coach, psychiatric mental health nurse, psychologist, peer support worker, volunteer, founder of intervention, advisor, coordinator, manager	p. 6
Non-participation	5 did not participate	p. 6
Setting of data collection	The interviews professionals were conducted via Microsoft Teams, Skype or telephone, between June and December 2022	p. 8
Presence of non-participants	No	Not reported
Description of sample		p. 6-7
Interview guide		p. 8
Repeat interviews	No	Not reported in manuscript
Audio/visual recordings	Audio for Skype and telephone, visual recordings for microsoft teams interviews	Not reported in manuscript
Field notes	Yes	Not reported
Duration	The interviews lasted from 40.4 to 85.4 minutes	p. 8

For peer review only

Data saturation	Interviews were conducted until data saturation occurred broadly by context, except for context C and F.	Not reported in manuscript
Transcripts returned	N/a	Not reported
**Domain 3: analysis and findings**		
Number of data coders	2	p. 8
Description of the coding tree		p.8
Derivation of themes	Themes were derived from data	p. 8
Software	Atlas.TI	p. 8
Participant checking	No	Not reported
Quotations presented	Yes, and each quotation is identified	p. 9-11
Data and findings consistent	Yes	
Clarity of major themes	Yes	p. 9-11, 16-19
Clarity of minor themes	Yes	p. 11-14

For peer review only

Professionals' perspectives on interventions to reduce problematic alcohol use in older adults: A realist evaluation of working elements.

Journal:	*BMJ Open*
Manuscript ID	bmjopen-2023-077851.R1
Article Type:	Original research
Date Submitted by the Author:	15-Jan-2024
Complete List of Authors:	van den Bulck, Fieke; Tilburg University Tranzo Scientific Centre for Care and Welfare, Tranzo<br>Knijff, Rikste; Tilburg University Tranzo Scientific Centre for Care and Welfare, Tranzo<br>Crutzen, Rik; School for Public Health and Primary Care (CAPHRI), Department of Health Promotion<br>van de Mheen, D; Tilburg University,<br>Bovens, Rob; Tilburg University Tranzo Scientific Centre for Care and Welfare, Tranzo<br>Stutterheim, Sarah; Maastricht University, Department of Work and Social Psychology<br>Van de Goor, Ien ; Tilburg University Tranzo Scientific Centre for Care and Welfare<br>Rozema, A; Tilburg University,
**Primary Subject Heading**:	Addiction
Secondary Subject Heading:	Qualitative research
Keywords:	Aged, Health Services for the Aged, QUALITATIVE RESEARCH

SCHOLARONE™ Manuscripts

I, the Submitting Author has the right to grant and does grant on behalf of all authors of the Work (as defined in the below author licence), an exclusive licence and/or a non-exclusive licence for contributions from authors who are: i) UK Crown employees; ii) where BMJ has agreed a CC-BY licence shall apply, and/or iii) in accordance with the terms applicable for US Federal Government officers or employees acting as part of their official duties; on a worldwide, perpetual, irrevocable, royalty-free basis to BMJ Publishing Group Ltd ("BMJ") its licensees and where the relevant Journal is co-owned by BMJ to the co-owners of the Journal, to publish the Work in this journal and any other BMJ products and to exploit all rights, as set out in our licence.

The Submitting Author accepts and understands that any supply made under these terms is made by BMJ to the Submitting Author unless you are acting as an employee on behalf of your employer or a postgraduate student of an affiliated institution which is paying any applicable article publishing charge ("APC") for Open Access articles. Where the Submitting Author wishes to make the Work available on an Open Access basis (and intends to pay the relevant APC), the terms of reuse of such Open Access shall be governed by a Creative Commons licence – details of these licences and which Creative Commons licence will apply to this Work are set out in our licence referred to above.

Other than as permitted in any relevant BMJ Author's Self Archiving Policies, I confirm this Work has not been accepted for publication elsewhere, is not being considered for publication elsewhere and does not duplicate material already published. I confirm all authors consent to publication of this Work and authorise the granting of this licence.

For peer review only

Professionals' perspectives on interventions to reduce problematic alcohol use in older adults: A realist evaluation of working elements.

F.A.E. van den Bulck[1], R. Knijff[1], R. Crutzen[2], D. van de Mheen[1], R.H.L.M. Bovens[1], S.E. Stutterheim[2], L.A.M. van de Goor[1], & A.D. Rozema[1]

[1] Tranzo Scientific Center for Care and Wellbeing, School of Social and Behavioral Sciences, Tilburg University, the Netherlands.

[2] Department of Health Promotion, Care and Public Health Research Institute (CAPHRI), Maastricht University, the Netherlands.

Affiliation addresses

[1] Professor Cobbenhagenlaan 125, Postbus 90153, 5000 LE Tilburg, The Netherlands

[2] Peter Debyeplein 1, 6229 HA Maastricht, The Netherlands

Corresponding author

F.A.E. van den Bulck

Email: f.a.e.vdnbulck@tilburguniversity.edu

Address: Professor Cobbenhagenlaan 125, Postbus 90153, 5000 LE Tilburg, The Netherlands

For peer review only

Abstract

Objectives

This study set out to understand how (i.e., which elements), in which context, and why (which mechanisms) interventions are successful in reducing (problematic) alcohol use among older adults, from the perspective of professionals providing these interventions.

Design

Guided by a Realist Evaluation approach, an existing initial program theory (IPT) on working elements in alcohol interventions was evaluated by conducting semi-structured interviews with professionals.

Setting and participants

These professionals (N=20) provide interventions across several contexts: with or without practitioner involvement; in-person or not; and in an individual or group setting. Data were coded and links between contexts, elements, mechanisms and outcomes were sought for to confirm, refute or refine the IPT.

Results

From the perspective of professionals, there are several general working elements in interventions for older adults: 1) *pointing out risks and consequences of drinking behavior*, 2) *paying attention to abstinence*, 3) *promoting contact with peers*, 4) *providing personalized content* and 5) *providing support*. We also found context-specific working elements, i.e., 1) *providing personalized conversations* and *motivational interviewing* with practitioners, 2) *ensuring safety, trust and a sense of connection* and *a location nearby home or a location that people are familiar with* in-person, and 3) *sharing experiences and tips* in group interventions. Furthermore, the mechanisms *awareness* and *accessible and low-threshold participation* were important contributors to positive intervention outcomes.

Conclusion

In addition to the IPT, our findings emphasize the need for social contact and support, personalized content, and strong relations (both between client and practitioner, and client and peers) in interventions for older adults.

Strengths and limitations of this study

Strengths and limitations of this study

- Using a Realist Evaluation provides in-dept insights in interventions because it can explain why interventions are successful or not.
- Data were collected through interviews with a variety of professionals across eleven different alcohol interventions
- Habitation bias and acquiescence bias could have occurred in data collection, which may influence the confirmed IPT.

For peer review only - http://bmjopen.bmj.com/site/about/guidelines.xhtml

INTRODUCTION

In recent decades, drinking behavior has increased, particularly in older adults (1-5). This has been accompanied with increased problematic alcohol use including high-risk drinking, binge drinking, and alcohol use disorders (6-8). Moreover, this change in drinking patterns has been associated with increased alcohol-related health problems in older adults due to biological changes in metabolism, body fat, body water, and the reduced ability of the liver to process alcohol (9, 10), and due to the combination alcohol with (increasing amounts of) medication when aging (11).

This increase in problematic alcohol use among older adults emphasizes the need for effective interventions. There are several existing approaches and specific interventions to reduce alcohol use, such as Screening, Brief Intervention, and Referral to Treatment (SBIRT), cognitive behavioral therapy (CBT) or motivational enhancement therapy, that are considered to be appropriate for older adults (12-16). However, most interventions to reduce (problematic) alcohol use in which older adults participate are designed for the general adult population; there is no consensus yet on what works best for older adults specifically (17, 18). Nonetheless, two systematic reviews found that effective intervention components to reduce alcohol use among older adults are personalized feedback, advice on (alcohol) behaviors, educational materials, and counseling (19, 20).

A systematic review which included 61 studies – using Realist Evaluation – focused on understanding how, in which context, and why interventions are successful in preventing or reducing (problematic) alcohol consumption among older adults (21). This review explored what works for people of 18+ individuals years and older (which includes older adults), and older adults specifically, and created an initial program theory based on the found working elements across six different contexts.

First, in interventions that were delivered in the context with practitioner involvement, in-person and to one individual (instead of a group), the working elements *paying attention to drinking behavior* and *the relationship between the client and practitioner* were identified. Second, in interventions that were delivered in the context with practitioner involvement, not in-person and individual, *personal contact and feedback* and *online communication and feedback* were identified. Third, in interventions that were delivered in the context with practitioner involvement, in-person and with involvement of relatives, *status of the relationship* and *teaching the partner to deal with drinking behavior* were identified. Fourth, in interventions that were delivered in the context with practitioner involvement, in-person and in group settings, *motivating to change lifestyle* (delivered in a workplace setting) was identified. Fifth, in interventions that were delivered in the context without practitioner involvement

For peer review only - http://bmjopen.bmj.com/site/about/guidelines.xhtml

and individual, *web based* and *telephone based* interventions were working elements. Sixth, in interventions that were delivered in the context without a practitioner, not in-person and in group setting, *focus on abstinence* was identified.

Overall, the results of the review showed three general working elements, i.e., 1) providing information about the consequences of alcohol consumption; 2) personalized feedback about drinking behavior; and 3) being in contact with others and communicating with them about (alcohol) problems (21). Of the 61 studies, only three evaluated interventions for older adults, and only the first and second general working element were found in these studies (22-24).

Evidently, in light of the small number of studies conducted specifically among older adults, more research on what works for older people is needed. The current study builds further on the previous performed review using Realist Evaluation.

Realist evaluation (RE) work from the assumption that interventions and their elements (E) work differently in different contexts (C). The context refers to the circumstances in which interventions operate, e.g. an online context in which an intervention is provided. Interventions may be successful in some contexts and not in others because the underlying processes through which the interventions bring change, called the mechanisms (M), are triggered to a different extent and lead to different intervention outcomes (O). Therefore, the interaction between the intervention elements (E), contexts (C), and mechanisms (E) play an important role in shaping the intervention outcomes (O). The strength of RE is that it offers a means to unpack the relationships between these contexts, elements, mechanisms, and outcomes. This is called the Context-Element-Mechanism-Outcome (CEMO) – configuration (25). RE starts with formulating an Initial Program Theory (IPT), based on the CEMOs founded in literature. Then the IPT needs to be confirmed, refused or refined using data that seeks to explain how the interventions work in real life contexts from the perspectives of those involved in its operation. Finally, based on the new data, the program theory might be refined.

This study is set out to confirm, refuse, or refine the IPT, consisting of the working elements, mechanisms and outcomes across six different contexts, as identified by Boumans et al (22). More specifically, the objective of this study is to understand from the perspective of professionals providing these interventions how (i.e., which elements), in which context, and why (which mechanisms) interventions are successful in reducing (problematic) alcohol use among older adults. The perspective

For peer review only - http://bmjopen.bmj.com/site/about/guidelines.xhtml

of professionals was considered important as they have extensive experience in addressing problematic alcohol use among older adults and a comprehensive understanding of what and why it works. Based on the perspectives of the professionals, CEMOs of the IPT will be confirmed, refuted or refined.

METHOD

Study context and design

In this qualitative study, we conducted semi-structured interviews with professionals providing interventions to older adults. This study is part of a larger qualitative research project that also investigates the perspectives of clients and close relatives of clients. Here, we focus on the professionals' perspectives. The larger study was approved by the Ethics Review Board at Tilburg University (RP508) and the COREQ guidelines were followed in reporting and design (26), see Supplementary Material 1.

Sampling and recruitment

In the current study, we set out to recruit and interview professionals providing various interventions across different organizations throughout the Netherlands.

First, we inventoried which interventions were used in practice by searching the Dutch National Database Centre for Healthy Living (*Centrum Gezond Leven*) and by consulting the national working group Elderly in the Dutch Partnership for Early Detection of Alcohol Problems (*Samenwerkingsverband Vroegsignalering Alcoholproblematiek*) and the national working group Alcohol and Elderly of The Dutch Addiction Association (*Verslavingskunde Nederland*). These two working groups comprise professionals from various organizations providing different interventions.

Second, based on this inventory, we included interventions if they 1) focused on universal, selective or indicated prevention; 2) were provided in an individual and/or group setting with or without relative involvement; 3) were provided face to face, online, and/or via telephone; 4) were provided in the Netherlands. Interventions were excluded if they 1) were not primarily aimed at lifestyle change; 2) were provided in a clinical setting; 3) were group-specific interventions (e.g., for veterans or athletes); or 4) focused on general health and lifestyle improvements without explicit mention of alcohol use.

Based on these selection criteria, the following Dutch alcohol interventions were included: Alcohol Information Line (*Alcohol Infolijn*), CBT for substance use (*CGT bij middelengebruik*), Enjoy Healthy (*Gezond Genieten*), Fresh Onwards, (*Fris Verder*), Maxx, Mirro, Moti-55, NoThanks (*IkPas*), Recovery-Guided Personal Program (*Herstel Ondersteunend Persoonlijk Programma*), Vitality days

For peer review only - http://bmjopen.bmj.com/site/about/guidelines.xhtml

(Vitaliteitsdagen), Snowball sampling among professionals helped us identify one other intervention: Drink Less (*MinderDrinken*)

Third, we approached professionals of the two working groups either working with these interventions or we approached professionals at organizations working with the interventions and asked to connect us with professionals that were involved in providing the interventions. A convenience sampling technique was used to recruit the professionals. Professionals that were interested received information about the study via email and some received information by telephone as well. They all received an information letter and informed consent form. Subsequently, they were invited for an interview.

In total, we contacted 25 professionals, of whom 20 participated. The remaining five did not respond to our emails. Participants' characteristics are shown in Table 1. Characteristics of included interventions are shown in Table 2.

Table 1. Participants' characteristics

Baseline characteristic	Participants
	n = 20
Gender	
Women	15
Men	5
Age (years)	
25-39	6
40-54	4
55-69	7
≥ 70	3
Experience with the intervention (in years)	
<1	2*
1	3
2	4
3	2
4	4
5	3
6	1
7	-
8	-
9	1
Role**	
Health care worker, prevention worker, coach, psychiatric mental health nurse or (GZ) psychologist	13
Peer support worker	1
Volunteer	3

For peer review only - http://bmjopen.bmj.com/site/about/guidelines.xhtml

Founder of intervention	3
Advisor, coordinator, manager	3
Intervention	
Alcohol Information Line	3
CBT for substance use	2
DrinkLess	1
Enjoy Healthy	1
Fresh Onward	4
Maxx	1
Mirro	1
Moti-55	2
NoThanks	2
Recovery guided personal program	1
Vitality days	2

* One professional was only involved in the development of the intervention.
** Some professionals had multiple roles in the same interventions, e.g. working as a coach and coordinator.

Data collection

Based on the participants preferences, online (80%) and telephone (20%) interviews were conducted between June and December 2022 with a median of 56.7 minutes (ranging from 40.4 and 85.4 minutes). Demographic variables including age, gender, and some questions about the intervention were asked prior to the interviews. All interviews were preceded by oral informed consent. We used an interview guide that measured the perceived outcomes of the interventions, the working elements and mechanisms related to the outcome(s), and the influence of the setting in which the interventions were provided. At the end of the interview guide, we presented the previous found CEMO configurations of the IPT of the review of Boumans et al. and invited participants to confirm, refute or refine the CEMOs and asked if they could comment on the CEMOs, see interview guide (see Supplementary Material 2). Interviews were conducted until data saturation occurred.

Patient and public involvement statement

None.

For peer review only - http://bmjopen.bmj.com/site/about/guidelines.xhtml

Table 2. Intervention characteristics

Intervention	Contact in intervention with			Setting		Sessions	Target group intervention	
	Professional	Peers	Relatives	Online, telephone	Face to face		Client's alcohol use **	Older adults (55+ or 65+)
Alcohol Information line	X			X		≥1	I	
CBT for substance use	X	X	X*		X	>1	IV	X
Drink Less	X*			X		N/a	III	
Enjoy Healthy	X	X			X	1	I	X
Fresh Onwards	X	X	X*		X	>1	IV	X
Maxx				X		N/a	III	
Mirro				X		N/a	III	
Moti-55	X		X*		X	>1	II	X
NoThanks	X*			X		N/a	III	
Recovery guided personal program	X		X*		X	>1	V	
Vitality days	X	X*	X*		X	1	I	X

* = The involvement of this person in the intervention is optional and depending on the client's preference

** I = People with all types of alcohol use patterns, II = People with early stage problematic alcohol use; III = People with all types of alcohol use patterns, except for (heavy) problematic alcohol use or alcohol addiction; IV = People with problematic alcohol use and alcohol addiction; V = People who are in recovery from addiction

For peer review only

Data analysis

Professional transcriptionists transcribed the interviews verbatim. Based on the IPT, FvdB drafted a code tree including CEMO configurations. Researchers FvdB and RK independently and, in parallel, coded six of the 20 interviews based on the RE approach, which means that the links between contexts, elements, mechanisms, outcomes were sought for within the data. After the first transcript was coded by FvdB and RK, FvdB cross-checked both their configurations and codes. FvdB and RK iteratively discussed differences in configurations or coding, and where relevant, refinements were made. These steps were repeated for all of the six initial interviews, at which time, coding was fully aligned. Thereafter, FvdB coded the remaining interviews independently. When in doubt about how to best code sections of the data, FvdB consulted with RK and ADR. Additionally, to further improve the quality of the coding, RK, RC, RB, IvdG, SS, and ADR reviewed formed codes and CEMOs iteratively and provided feedback. Codes were then adjusted if necessary and some new codes were added. Data were analysed using Atlas.TI.

RESULTS

Results are categorized according to the context in which the interventions are delivered: 1) involvement or lack of involvement of a practitioner that provides the intervention directly to the participant; 2) provision of the intervention in-person or not; and 3) individual treatment, group treatment or treatment with relatives' involvement. We found data across six combinations of contexts that the IPT conformed to. Below, we describe the working intervention elements (e) and Table 3 provides an overview of the key working elements, mechanisms, and outcomes, mentioned by more than one participant. Table 4 provides an overview of the summarized findings of the program theory. For an even more comprehensive table including all CEMOs, even those mentioned by one respondent, see Supplementary Table 1.

A. Practitioner—in-person—individual

Professionals confirmed that (1) *paying attention to drinking behavior* (e) is important in interventions to reduce alcohol use (o) because it could stimulate clients to start thinking about their alcohol use (m) and create awareness of their use (m).

Alcohol is of course something that has a risk, can cause health damage. People think about that 'Do I want to do that to myself? I'm getting older, I'm getting more forgetful' So they start to think a lot more about what they are doing to themselves because of alcohol use. If you actually tell them that as

For peer review only - http://bmjopen.bmj.com/site/about/guidelines.xhtml

a professional, then people really think twice about it: 'Wait a minute, I still want to enjoy my life and I want to age healthy'. [R9, prevention worker]

They also emphasized the importance of (2) *paying attention to lifestyle* (e) and (3) using specific *communication approaches* (e), e.g. motivational interviewing. The professionals confirmed that (4) *the relationship between client and practitioner* (e) is important because it could trigger open communication (m), and collaboration between client and practitioner that improves the relationship between practitioner and client (m). Finally, they mentioned that a (5) *favorable setting* (e) in which the intervention is provided is important, i.e., a location that is nearby because it contributes to low-threshold participation (m).

B. Practitioner—not in-person—individual

Professionals confirmed that personal contact combined with feedback reduced alcohol use (o). In addition, they also mentioned several other (1) *communication approaches* (e), e.g. motivational interviewing, personalized contact, and anonymous participation. These approaches triggered different mechanisms, i.e., tailoring (m), and low-threshold and flexible participation (m).

And certainly with older people because they often already have certain habits for years or perhaps have a bad marriage or a very stressful job. So those life-stage issues, you can sort those out in personal treatment. [R3, psychologist and advisor]

It also makes it low threshold for people to call. We often have people on the phone who admit for the first time at all that something is going on. […] For example, people often call in their car and I think that is also a nice anonymous place where no one, no family member, a colleague, can see who they are calling. […] People can choose when and where they have this conversation, so they can choose a time and location that they prefer.[R4, health care worker]

The professionals added that (2) *the relationship between client and practitioner* (e)is important, e.g. when the professional is empathic, supportive and listens to the client because this could trigger engagement (m) followed by open communication where the client accepts advice or information from the practitioner (m). Finally, they also mentioned that (3) *providing additional help* (e) can be important because it could provide extra attention (m) and tailoring (m).

C. Practitioner—in-person—relatives

For peer review only - http://bmjopen.bmj.com/site/about/guidelines.xhtml

Professionals confirmed that a partner is more understanding and supportive for the client (m) when (1) *the partner is taught to deal with the drinking behavior of the client* (e), and that (2) *support of relatives* (e) can lead to alcohol reduction (o) among clients.

> *It is important for them to understand how addiction works, that it's not solved at once and that it is actually a disease. […] . So, the relative regains a bit more confidence and yes, this often creates space in which they can grow together and take steps. On the one hand, this strengthens the client and, on the other hand, it gives a relative more confidence that it can be done.* [R16, peer support worker]

D. Practitioner—in-person—group component

Professionals mentioned that (1) *paying attention to drinking behavior* (e) is important because it contributes to reductions in alcohol use (o). They confirmed that (2) *paying attention to lifestyle* (e) was also a working element in this context because it motivates people to participate in the intervention and to change (m), which ultimately contributes to reduced alcohol use (o). In addition, they also mentioned that (3) *communication approaches* (e), including motivational interviewing, and personalized contact and conversations were important. Professionals also mentioned that (4) *the relationship between client and peers* (e) plays an important role in interventions because it could provide recognition, connection, support, hope, safety, and new insights (m) for the client.

> *There is often a lot of negativities in the family atmosphere and I think in a group with peers it is accepted that people have a problem and they start looking to get it under control. So there is engagement, an open and accepting attitude that makes people feel safe and I think that is very important in a group, that people feel very safe to talk about it without feeling judged.* [R13, MH psychologist]

Again, (5) *the relationship between the client and practitioner* (e), for example when the professional has an open attitude and does not judge the client, was important in interventions according to the professionals. Providing (6) *other activities* (e) such as eating after a joint session were helpful because they contribute to making a connection (m) and pleasant atmosphere (m).

Finally, (7) a *favorable setting* was important because it contributed to low-threshold participation.

For peer review only - http://bmjopen.bmj.com/site/about/guidelines.xhtml

It is often a very high threshold for people to go to an addiction center. So, I think that this kind of treatment could also be given in a community entre, or a nursing home, or at the GP in a small group, or something. [R14, psychiatric mental health nurse]

E. No practitioner—not in-person—individual

Professionals mentioned that (1) *paying attention to drinking behavior* (e) contributes to reductions in alcohol use (o). Also (2) *using tools* (e), e.g. an online self-help tool, was considered beneficial in this setting because it enables low-threshold participation (m) for participants and allowed them to be actively involved (m). Again, (3) *the relationship between client and peers* (e) was important, because it provided clients with togetherness, recognition and support (m).

I think that for this target group it is very welcome that, additionally in an online setting, they can do it alone or with a friend, or with a group where you meet once per week or two weeks. [RP6, founder of intervention and manager]

Also, (4) *a favorable setting* (e) was important because it could make participation low-threshold (m). Finally, (5) *additional help* (e), i.e., providing personalized content and help additional to self-help, was also seen as effective.

F. No practitioner—not in-person—group component

Professionals mentioned that (1) *paying attention to drinking behavior* (e), (2) *self-help groups* (e) and (3) *the relationship between the client and peers* (e) were all important in interventions in this context. No mechanisms were found that were mentioned by more than one professional.

Table 3. The program theory

Context	Element of intervention	Mechanism	Outcome	Target group**	IPT ***
A. Practitioner – in-person – individual	1. *Paying attention to drinking behavior*				
	1.1. * Paying attention to drinking behavior	Think and act differently about alcohol use	Less or no alcohol use	X	✓
	1.2. Tracking alcohol use and reflecting on use	a. Creating insight into use<br>b. Thinking about alcohol use	Less or no alcohol use<br>Awareness	II, III, IV, X	+
	1.3. Paying attention to abstinence, coping planning and practicing with abstinence	Raising awareness alcohol use		I, IV, X	+
	1.4. Pointing out the risks and consequences of drinking behavior	Thinking about indirect consequences of use	Awareness, less or no alcohol use	I, II, IV	+
	2. *Paying attention to lifestyle*				
	2.1. Paying attention to meaning of life, and teaching lifestyle changes		Less or no alcohol use	II	+
	3. *Communication approaches*				
	3.1. Motivational interviewing			II, X	+
	3.2. Personalized content and conversation			I, II	+
	4. *The relationship between the client and*				

For peer review only - http://bmjopen.bmj.com/site/about/guidelines.xhtml

	practitioner				
	4.1. Safety, trust, connection, and not being judged in contact with professional	Having open communication	Less alcohol use	II, X	+
	4.2. * Empathic behavior of practitioner	Collaborating in identification of help and needs, and improve relationship between client and practitioner	Less or no alcohol use	I, X	✓
	4.3. Critical, controlling and confronting behavior of practitioner			II, IV, X	+
	5. *Favorable setting*				
	5.1. Location is nearby home and/or familiar	Low-threshold participation		I, IV	+
B. Practitioner – not in person – individual	1. *Communication approaches*				
	1.1. Contact always possible	Accessible help in difficult situations		I, III	+
	1.2. Conversation over the phone	Accessible and flexible participation		I, X	+
	1.3. Anonymous	a Distance in contact, making client more likely to accept advice<br>b. Low-threshold<br>c. Honest instead of social desirable contact	Think differently about alcohol use	I	+
	1.4. Motivational interviewing		Awareness	I, X	+
	1.5. Personalized content and conversation	a. Tailoring		I, III	+
		b. Non-commitment disappears	Less or no alcohol use	I, III	+
		c. Emotional support		I, III, X	+
	1.6. * Personal contact and feedback		Less or no alcohol use	X	✓
	1.7. * Online communication and feedback		Less or no alcohol use		-
	2. *The relationship between the client and practitioner*				
	2.1. Practitioner is empathic, supportive and listens	Engaging, open communication and client accepts advice or information from practitioner		I	+
	3. *Providing additional help*				
	3.1. Personal treatment with practitioner additional to selfhelp	Extra attention, tailoring		III, X	+
C . Practitioner—in-person—relatives	. *Teaching the partner to deal with drinking behavior*				
	1.1. * Teaching the partner to deal with drinking behavior	More understanding and support from the relative for the client		I, IV, X	✓
	2. Support of relatives				
	2.1. Relative provides support		Less or no alcohol use	X	+
D. Practitioner—in-person—group component	1. *Paying attention to drinking behavior*				
	1.1. Pointing out the risks and consequences of drinking behavior			IV, V	+
	1.2. Paying attention to abstinence and practicing with abstinence		Less or no alcohol use	IV	+
	2. *Paying attention to lifestyle*				
	2.1. *Motivating to change lifestyle				~
	2.2. Attention and tips regarding meaning of life, lifestyle and problems when aging	Motivation for participation and change	Less or no alcohol use, know how to improve life quality during abstinence	IV, X	+
	2.3. *Motivating to change lifestyle delivered in a workplace setting				~
	2.4. In a workplace setting and paying attention to prevention and lifestyle			X	+
	3. *Communication approaches*				
	3.1. Motivational interviewing		Less or no alcohol use, awareness	IV, X	+
	3.2. Personalized content and conversation			IV, X	+
	4. *The relationship between the client and peers*				
	4.1. Contact with peers	Recognition, connection and support		IV, V, X	+
	4.2. Closed group and maximized group size	a. Create an emotionally safe space<br>b. More attention per client		I, IV	+
	4.3. Engagement, understanding and support towards peers	Togetherness, connection and safety	Less or no alcohol use	IV	+
	4.4. Addressing peers	Gain new insights	Less or no alcohol use	II, III, IV, V	+
	4.5. Sharing experiences and tips with peers	a. Being hopeful in own process		IV, V, X	

For peer review only - http://bmjopen.bmj.com/site/about/guidelines.xhtml

		b. Feeling recognized, relativating own situation			+
	5. *The relationship between the client and practitioner*				
	5.1. Open attitude, not being judged and accessible contact with practitioner			V	+
	6. *Other activities*				
	6.1. Having lunch after every session	Making connection and pleasant atmosphere		IV, V	+
	7. *Favorable setting*				
	7.1. Pleasant and relaxed atmosphere			I, IV	+
	7.2. Location is nearby home and/or familiar	Low-threshold participation		I, IV	+
E. No practitioner—not in-person—individual	1. *Paying attention to drinking behavior*				
	1.1. Tracking alcohol use	Insight into use and tracking progress	Less or no alcohol use	III, X	+
	1.2. Abruptly quit alcohol use	Positive learning experience		II, III	+
	1.3. Pointing out the risks and consequences of drinking behavior			III	+
	2. *Using tools*				
	2.1. *Web and telephone based interventions				~
	2.2. Online self-help tool	Accessible, low-threshold and controlled participation	Awareness, less or no alcohol use, going to treatment	III, X	+
	2.3. Via telephone			X	+
	2.4. Regular newsletter	Being actively engaged		III	+
	3. *The relationship between the client and peers*				
	3.1. Joining with others, contact with peers	Togetherness, recognition and support		III	+
	4. *Favorable setting*				
	4.1. Location independent participation	Accessible and low-threshold		III	+
	5. *Additional help*				
	5.1. Personalized content and help additional to self-help			III	+
F. No practitioner – not in-person – group component	1. *Paying attention to drinking behavior*				
	1.1.*Intervention to abstinent people				~
	1.2. Focus on abstinence and paying attention to withdrawal			X	+
	2. *Self-help groups*				
	2.1. AA and self-help group			X	+
	3. *The relationship between the client and peers*				
	3.1.Contact with peers			IV, X	+
	3.2. Sharing experiences and tips with peers			IV, X	+

* Elements of Initial program theory.
** Target group of the intervention. I = People with all types of alcohol use patterns, II = People with early-stage problematic alcohol use; III = People with all types of alcohol use patterns, except for (heavy) problematic alcohol use or alcohol addiction; IV = People with problematic alcohol use and alcohol addiction; V = People who are in recovery from addiction, X = No target group mentioned because these elements were derived from CEMO configuration that we presented as statements during the interviews.
***=✓ = Initial theory confirmed, ~ = Initial theory refined, + = New theory added − = Initial theory not confirmed.

Table 4. Summary of the program theory

Context	Element of intervention	Mechanism*	Outcome
A. Practitioner – in-person – individual	Paying attention to drinking behavior	Changing perceptions on own use (1.1, 1.2.a., 1.2.b., 1.3.) and perceptions consequences (1.4.)	Less or no alcohol use, awareness
	Paying attention to lifestyle		
	Communication approaches		
	The relationship between the client and practitioner	Improving communication (4.1.) and collaboration with practitioner (4.2.)	
	Favorable setting	Low-threshold participation (5.1.)	
B. Practitioner – not in person – individual	Communication approaches	Low- threshold participation (1.3.b), tailoring (1.1., 1.2., 1.5.a.), improving communication with practitioner (1.3.a., 1.3.c.), engagement (1.5.b), and emotional support (1.5.c)	Less or no alcohol use, awareness, changing perspective on own alcohol use
	The relationship between the client and practitioner	Engagement and improving communication with practitioner (2.1.)	
	Providing additional help	Tailoring (3.1.)	
C . Practitioner—in-person—relatives	Teaching the partner to deal with drinking behavior	More understanding and support from the relative for the client (1.1.)	Less or no alcohol use
D. Practitioner—in-	Paying attention to drinking behavior		Less or no alcohol

For peer review only - http://bmjopen.bmj.com/site/about/guidelines.xhtml

person—group component	Paying attention to lifestyle	Motivation (2.2.)	use, awareness
	Communication approaches		
	The relationship between the client and peers	Social and emotional support (4.1., 4.3., 4.5.b.), pleasant atmosphere (4.2.a.), tailoring (4.2.b.), and changing perceptions on own use (4.4., 4.5.a., 4.5.b.)	
	The relationship between the client and practitioner		
	Other activities	Social contact and pleasant atmosphere (6.1.)	
	Favorable setting	Low-threshold participation (7.2.)	
E. No practitioner— not in-person— individual	Paying attention to drinking behavior	Changes perceptions on own use (1.1., 1.2.)	Less or no alcohol use
	Using tools	Low-threshold (2.2.) and engagement (2.4.)	
	The relationship between the client and peers	Social and emotional support (3.1.)	
	Favorable setting	Low-threshold participation(4.1.)	
	Additional help		
F. No practitioner – not in-person – group component	Paying attention to drinking behavior		
	Self-help groups		
	The relationship between the client and peers		

*= The mechanisms that are presented are the summarized mechanisms from Table 3, along with the numbering of the original mechanisms as indicated in Table 3.

DISCUSSION

Key findings

Overall, five elements that were mentioned by more than one respondent were found in minimally three contexts and can be labelled as general working elements: 1) *pointing out the risks and consequences of drinking behavior*, 2) *paying attention to abstinence*, 3) *promoting contact with peers*, 4) providing *personalized content* and 5) *providing support*. We also found context-specific working elements, namely *providing personalized conversations* and *motivational interviewing,* specifically in interventions with practitioner involvement; *ensuring safety, trust and a sense of connection* and *a location nearby home or a location that people are familiar with* in in-person interventions; and *sharing experiences and tips* in interventions in group settings. Moreover, two mechanisms that were mentioned by more than one respondent were found in minimal in three contexts: *awareness* and *accessible and low-threshold participation*. Our findings are generally in line with the initially formed program theory. However, we found many additional working elements, mechanisms and outcomes that were posited by several professionals resulting in a refined program theory.

Interpretation of findings

The first addition to the IPT is that older adults need social contact and support. *Promoting contact with peers* can help reduce loneliness or social isolation, and *providing support* from a practitioner, peers or relatives can help them to deal with negative emotions and problematic alcohol use. Compared to the general adult population, older adults are facing more often retirement, illness, loss of relatives, loneliness, or hopelessness, which may facilitate negative health consequences of

For peer review only - http://bmjopen.bmj.com/site/about/guidelines.xhtml

alcohol use (27, 28). It is important to facilitate the process of helping older adults to develop contacts, for example in leisure settings *on a location that is nearby home and/or familiar* to make participation low-threshold.

The second addition to our IPT is that not only personalized feedback, but also *providing personalized content and conversations* are helpful for older adults, which is in line with findings in previous research showing that a personal approach with personalized feedback (22-24) and personalized reports (22, 23) are effective for older adults. Understanding the client's motives for alcohol use can help providers to be more specific and effective for providing personalized and tailored interventions. A tailored approach with consideration of the client's needs with aging-related challenges taken into account appears to be very effective(29) .

The third addition to our IPT is that both *the relationship between client and the practitioner* and *the relationship between client and peers* are important in interventions. Previous research showed that therapeutic alliance in the relationship between client and practitioner is associated with a reduced likelihood of dropout (30). Furthermore, older adults with substance use problems prefer interventions that are led by warm, honest, caring and relatively non-confrontational providers (31). These findings are in line with our working element *ensuring safety, trust and a sense of connection* and the importance of *the relationship between client and provider*.

In contrast, previous research showed mixed findings on the relationship between client and peers in substance use disorder populations: some research has found a significant association between group cohesion and intervention outcomes, and group processes and outcomes (32-37), while other research has found no significant association between group cohesion and outcomes (38, 39). Even though there seems to be no consensus in literature in the association between the relationship between client and peers, and intervention outcomes for the general adult population, we argue that there could be a strong association for older adults. Age-specific treatments may work better with older adults than mixed-age treatments (40). A group composition that matches the preferences of older adults could be a pre-requisite to *ensure safety, trust and a sense of connection*, which contribute to positive outcomes.

Finally, we found two important mechanisms that contribute to positive interventions outcomes. First, awareness is an important mechanism in preventing or reducing problematic alcohol use. A

For peer review only - http://bmjopen.bmj.com/site/about/guidelines.xhtml

previous study by Veerbeek and colleagues (2017) reported that older adults have limited awareness of the negative health effects of alcohol, and that the majority of older adults in their study did not think alcohol is addictive or unhealthy. In addition, the current standard for responsible alcohol consumption is often known to only a small proportion among older adults (41), and people with problematic alcohol use often do not recognize their own drinking pattern as problematic(42, 43). . This lack of knowledge emphasizes the importance of the working element *pointing out the risks and consequences of drinking behavior among* older adults. Emphasizing these risks can be experienced as threatening and therefore it is important to enhance older adults self-efficacy in the intervention first (44). Furthermore, knowledge is a fundamental first step, but information provision alone is not sufficient for behavior change (45). Second, *accessible and low-threshold participation* is an important mechanism in interventions for older adults. A major barrier for older adults seeking help is that they do not want to be labelled an 'alcoholic' or 'addict' (41), which can make participation in interventions that are provided in addiction care centres high threshold. Providing interventions on a location that people do not directly associate with alcoholism, e.g. a community center, can make participation low-threshold. Another barrier for older adults can be difficulties with transportation (31). Travel allowance and accessibility of interventions can help older adults overcome these barriers.

Limitations and strengths

This study has some limitations. In the Netherlands, people with lower socio-economic status and ethnic minorities are often not reached by lifestyle intervention efforts and show low participation rates (46, 47). It is possible that the included interventions in this study are less likely to reach these groups, which could mean are that the professionals have limited experiences with the subgroups and that the overview of working elements and mechanisms in not fully adequate for these subgroups. It is therefore important to provide interventions with *personalized content and conversations* for specific subgroups, because it can provide a tailored approach. For example, older adults with a language deficiency could benefit from more specific elements in communication with the professional. Also, cultural differences may ask for different approaches. A study showed that religious older Moroccan-Dutch and Turkish-Dutch women can have other motivators (e.g. religious or cultural) to abstain from alcohol use than older native Dutch women had (48). Further research should focus on what works for specific subgroups in older adults and to what extent these older adults participate in interventions to reduce problematic alcohol use. The second limitation concerns biases when the CEMO statements were presented at the end of the interview. Possibly acquiescence bias occurred here, meaning that respondents agreed more

For peer review only - http://bmjopen.bmj.com/site/about/guidelines.xhtml

easily to these statements, e.g. because they responded in ways they thought the researcher wanted them to respond, or because they wanted to finish the interview. Respondents were invited to explain their opinions on these statements, but they often responded by only saying that they agreed to these statements without explanation. Habituation bias could have occurred as well when the participants provided the same answers in response to similarly-worded presented CEMO statements. These two biases may influence our confirmed IPT. Finally, some CEMO configurations were only confirmed when the statements were presented. Some working elements, e.g. empathic behavior of the practitioner, might be so obvious that they were therefore initially not mentioned. We think that there could be more of such elements that are missing in our overview because of these reasons.

A strength of this study is that we were able to include a variety of professionals across all eleven interventions, and the high response rate. Another strength is the Realist Evaluation approach was used, as this provides explanations from the perspective of professionals in what works to reduce problematic alcohol use among older adults. This approach provides in-dept insights in interventions because it can explain why interventions are successful or not.

Practical and research implications

Based on our findings, we have a number of recommendations for practice and future research. Professionals and developers of interventions might focus on three aspects. First, practitioners should have attention for the elements that are related to the role of the practitioner in in-person interventions, more specifically in existing brief interventions which are already often used by practitioners. Second, developers of interventions need to pay particular attention to accessible or low-threshold participation, for example by providing interventions in a *favorable setting*. Third, practitioners and developers together should focus more on the element *promoting contact with peers*. For example, practitioners working in group interventions can invite clients to *share experiences and tips* with peers, and developers of online self-help tools can refer people to online forums where people can *share experiences and tips* with peers.

Further research should focus on perspectives of clients who participate in interventions and their relatives, to refine the program theory. Also, further research should focus on interventions in the context with practitioner, not in-person, and individual and in context with no practitioner, not in-person, and in groups is warranted, because elements in these contexts are limited in this study.

For peer review only - http://bmjopen.bmj.com/site/about/guidelines.xhtml

CONCLUSION

In addition to the previous formulated IPT, our findings emphasize the need for social contact and support, personalized content, and strong relations (both between client and practitioner, and client and peers) in interventions for older adults. Whereas the working element 'social contact' was not found in previous literature as a working element, probably due to limited performed studies, our study found this as an additional important working element, and providers of alcohol interventions should be aware and make use of this element when treating older adults. Future research should study perspective of older adults themselves and their relatives on working elements and mechanisms in interventions.

Contributors

FvdB, ADR, RC, RB and DvdM developed the research questions and study methodology. FvdB and ADR developed an interview guide an RC, RB and DvdM provided inputs to the interview guide. Data were collected by FvdB. Data were coded by FvdB and co-coded by RK, and checked by ADR. ADR, RC, RB, DvdM, IvdG, and SS then provided feedback on the code-document. FvdB produced the draft manuscript under daily supervision of ADR. ADR, RC, RB, DvdM, IvdG, and SS have revised the manuscript critically for important content.

Funding

This research was funded by The Netherlands organisation for Health Research and Development (ZonMw number: 555002034).

Competing interests

The authors declare no conflict of interest.

Patient and public involvement

Patients and/or the public were not involved in the design, conduct, reporting, or dissemination plans of this research.

Ethics approval

The study was approved by the Ethics Review Board at Tilburg University (RP508). Participants gave informed consent to participate in the study before taking part.

Data availability statement

For peer review only - http://bmjopen.bmj.com/site/about/guidelines.xhtml

No additional data available.

REFERENCES

1. Breslow RA, Castle IJP, Chen CM, Graubard BI. Trends in Alcohol Consumption Among Older Americans: National Health Interview Surveys, 1997 to 2014. Alcoholism: Clinical and Experimental Research. 2017;41(5):976-86.
2. Han BH, Moore AA, Sherman S, Keyes KM, Palamar JJ. Demographic trends of binge alcohol use and alcohol use disorders among older adults in the United States, 2005–2014. Drug and Alcohol Dependence. 2017;170:198-207.
3. Kim JW, Lee DY, Lee BC, Jung MH, Kim H, Choi YS, et al. Alcohol and Cognition in the Elderly: A Review. Psychiatry Investigation. 2012;9(1):8.
4. Roche AM, Kostadinov V. Baby boomers and booze: we should be worried about how older Australians are drinking. Medical Journal of Australia. 2019;210(1):38-9.
5. Wolf I-K, Du Y, Knopf H. Changes in prevalence of psychotropic drug use and alcohol consumption among the elderly in Germany: results of two National Health Interview and Examination Surveys 1997-99 and 2008-11. BMC Psychiatry. 2017;17(1).
6. Mauro PM, Askari MS, Han BH. Gender differences in any alcohol screening and discussions with providers among older adults in the United States, 2015 to 2019. Alcoholism: Clinical and Experimental Research. 2021;45(9):1812-20.
7. Grant BF, Chou SP, Saha TD, Pickering RP, Kerridge BT, Ruan WJ, et al. Prevalence of 12-Month Alcohol Use, High-Risk Drinking, and <i>DSM-IV</i> Alcohol Use Disorder in the United States, 2001-2002 to 2012-2013. JAMA Psychiatry. 2017;74(9):911.
8. Knott CS, Scholes S, Shelton NJ. Could more than three million older people in England be at risk of alcohol-related harm? A cross-sectional analysis of proposed age-specific drinking limits. Age Ageing. 2013;42(5):598.
9. Meier P, Seitz HK. Age, alcohol metabolism and liver disease. Current Opinion in Clinical Nutrition and Metabolic Care. 2008;11(1):21.
10. Cederbaum AI. Alcohol Metabolism. Clinics in Liver Disease. 2012;16(4):667-85.
11. Moore AA, Whiteman EJ, Ward KT. Risks of combined alcohol/medication use in older adults. The American Journal of Geriatric Pharmacotherapy. 2007;5(1):64-74.
12. Kuerbis A, Sacco P, Blazer DG, Moore AA. Substance abuse among older adults. Clinics in geriatric medicine. 2014;30(3):629-54.
13. Schonfeld L, Dupree LW. Treatment Approaches for Older Problem Drinkers. International Journal of the Addictions. 1995;30(13-14):1819.
14. Dupree LW, Broskowski H, Schonfeld L. The Gerontology Alcohol Project: A Behavioral Treatment Program for Elderly Alcohol Abusers. The Gerontologist. 1984;24(5):510.
15. Rice C, Longabaugh R, Beattie M, Noel N. Age group differences in response to treatment for problematic alcohol use. Addiction. 1993;88(10):1369-75.
16. Schonfeld L, Hazlett RW, Hedgecock DK, Duchene DM, Burns LV, Gum AM. Screening, brief intervention, and referral to treatment for older adults with substance misuse. American journal of public health. 2015;105(1):205-11.
17. Seddon JL, Wadd S, Wells E, Elliott L, Madoc-Jones I, Breslin J. Drink wise, age well; reducing alcohol related harm among people over 50: a study protocol. BMC Public Health. 2019;19(1).
18. Schultz SK, Arndt S, Liesveld J. Locations of facilities with special programs for older substance abuse clients in the US. International Journal of Geriatric Psychiatry. 2003;18(9):839-43.

For peer review only - http://bmjopen.bmj.com/site/about/guidelines.xhtml

19. Armstrong-Moore R, Haighton C, Davinson N, Ling J. Interventions to reduce the negative effects of alcohol consumption in older adults: a systematic review. BMC Public Health. 2018;18:13.
20. Megherbi-Moulay O, Igier V, Julian B, Franchitto N, Sordes F. Alcohol Use in Older Adults: A Systematic Review of Biopsychosocial Factors, Screening Tools, and Treatment Options. International Journal of Mental Health and Addiction. 2022.
21. Boumans J, van de Mheen D, Crutzen R, Dupont H, Bovens R, Rozema A. Understanding How and Why Alcohol Interventions Prevent and Reduce Problematic Alcohol Consumption among Older Adults: A Systematic Review. Int J Environ Res Public Health. 2022;19(6):3188.
22. Ettner SL, Xu H, Duru OK, Ang A, Tseng C-H, Tallen L, et al. The Effect of an Educational Intervention on Alcohol Consumption, At-Risk Drinking, and Health Care Utilization in Older Adults: The Project SHARE Study. Journal of Studies on Alcohol and Drugs. 2014;75(3):447-57.
23. Fink A, Elliott MN, Tsai M, Beck JC. An Evaluation of an Intervention to Assist Primary Care Physicians in Screening and Educating Older Patients Who Use Alcohol. Journal of the American Geriatrics Society. 2005;53(11):1937-43.
24. Kuerbis AN, Yuan SE, Borok J, Lefevre PM, Kim GS, Lum D, et al. Testing the Initial Efficacy of a Mailed Screening and Brief Feedback Intervention to Reduce At-Risk Drinking in Middle-Aged and Older Adults: The Comorbidity Alcohol Risk Evaluation Study. Journal of the American Geriatrics Society. 2015;63(2):321-6.
25. Pawson R, Tilley N, Tilley N. Realistic Evaluation: SAGE Publications; 1997.
26. Tong A, Sainsbury P, Craig J. Consolidated criteria for reporting qualitative research (COREQ): a 32-item checklist for interviews and focus groups. International Journal for Quality in Health Care. 2007;19(6):349.
27. Kuerbis A. The impact of retirement on the drinking patterns of older adults: a review. Addictive Behaviors. 2012;37(5):587.
28. Barry KL. Drinking over the lifespan: Focus on older adults. Alcohol Research: Current Reviews. 2016;38(1):115.
29. Atiq Rafay R. Common themes and issues in geriatric psychotherapy. Psychiatry (Edgmont (Pa : Township)). 2006;3(6):53-6.
30. Meier PS, Donmall MC, McElduff P, Barrowclough C, Heller RF. The role of the early therapeutic alliance in predicting drug treatment dropout. Drug and Alcohol Dependence. 2006;83(1):57-64.
31. Holland JM, Rozalski V, Beckman L, Rakhkovskaya LM, Klingspon KL, Donohue B, et al. Treatment Preferences of Older Adults with Substance Use Problems. Clinical Gerontologist. 2016;39(1):15-24.
32. Valeri L. Group therapy for women with substance use disorders: In-session affiliation predicts women's substance use treatment outcomes. Journal of Substance Abuse Treatment. 2018;94:60.
33. Kelly PJ, Deane FP, Baker AL. Group Cohesion and Between Session Homework Activities Predict Self-Reported Cognitive–Behavioral Skill Use Amongst Participants of SMART Recovery Groups. Journal of Substance Abuse Treatment. 2015;51:53-8.
34. Cavicchioli M. Group relationships during a dialectical behavior therapy skills training program for the treatment of alcohol and concurrent substance use disorders: Evidence and theoretical considerations. Group Dynamics: Theory, Research, and Practice. 2021;25(2):152.
35. Sotskova A. Understanding the role of group cohesion and group alliance in a secular peer support group for recovery from substance misuse. Journal of Groups in Addiction and Recovery. 2016;11(2):137.
36. Rice SL, Tonigan JS. Impressions of Alcoholics Anonymous (AA) Group Cohesion: A Case for a Nonspecific Factor Predicting Later AA Attendance. Alcoholism Treatment Quarterly. 2012;30(1):40-51.
37. Pooler DK, Qualls N, Rogers R, Johnston D. An Exploration of Cohesion and Recovery Outcomes in Addiction Treatment Groups. Social Work with Groups. 2014;37(4):314-30.

For peer review only - http://bmjopen.bmj.com/site/about/guidelines.xhtml

38. Cruz M, Osilla KC, Paddock SM. Group Cohesion and Climate in Cognitive Behavioral Therapy for Individuals with a First-Time DUI. Alcoholism Treatment Quarterly. 2020;38(1):68-86.
39. Gillaspy JA, Wright AR, Campbell C, Stokes S, Adinoff B. Group Alliance and Cohesion as Predictors of Drug and Alcohol Abuse Treatment Outcomes. Psychotherapy Research. 2002;12(2):213-29.
40. Kuerbis A, Sacco P. A Review of Existing Treatments for Substance abuse among the Elderly and Recommendations for Future Directions. Substance Abuse: Research and Treatment. 2013;7:SART.S7865.
41. Veerbeek M, Heijkants C, Willemse B. Alcoholgebruik onder 55-plussers. Utrecht: Trimbos Instituut. 2017.
42. Smith JJ, Spanakis P, Gribble R, Stevelink SAM, Rona RJ, Fear NT, et al. Prevalence of at-risk drinking recognition: A systematic review and meta-analysis. Drug and Alcohol Dependence. 2022;235:109449.
43. Morris J, Albery IP, Moss A, Heather N. Promoting problem recognition amongst harmful drinkers: A conceptual model for problem framing factors. The Handbook of Alcohol Use. 2021:221.
44. Peters G-JY, Ruiter RAC, Kok G. Threatening communication: a critical re-analysis and a revised meta-analytic test of fear appeal theory. Health Psychology Review. 2013;7(sup1):S8-S31.
45. Arlinghaus KR, Johnston CA. Advocating for Behavior Change With Education. American Journal of Lifestyle Medicine. 2018;12(2):113-6.
46. El Fakiri F, Hoes AW, Uitewaal PJM, Frenken RAA, Bruijnzeels MA. Process evaluation of an intensified preventive intervention to reduce cardiovascular risk in general practices in deprived neighbourhoods. European Journal of Cardiovascular Nursing. 2008;7(4):296.
47. Magnée T, Burdorf A, Brug J, Kremers SPM, Oenema A, Van Assema P, et al. Equity-Specific Effects of 26 Dutch Obesity-Related Lifestyle Interventions. American Journal of Preventive Medicine. 2013;44(6):e61-e70.
48. Sialino LD, Wijnhoven HAH, Van Oostrom SH, Picavet HSJ, Verschuren WMM, Visser M, et al. Perspectives of older women in the Netherlands: identifying motivators and barriers for healthy lifestyles and determinants of healthy aging. BMC Public Health. 2023;23(1).

For peer review only

Supplementary Material 1

The COREQ Checklist

**Domain 1: Research team and reflexivity**		
Personal characteristics		
Interviewer/facilitator	Fieke van den Bulck	n/a
Credentials	MSc	Not reported in manuscript
Occupation	PhD	Not reported in manuscript
Gender	Woman	Not reported in manuscript
Experience and training	Conducted interviews as a part of her Ph.D.	Not reported in manuscript
Relationship with participants		
Relationship established	yes	Not reported in manuscript
Participant knowledge of the interviewer	Created relationship during preparation for the research study .	Not reported in manuscript
Interviewer characteristics		Not reported
**Domain 2: Study design**		
Theoretical framework		
Methodological orientation	Interviews with a realist evaluation approach	p. 5, 8
Participant selection		
Sampling	Purposive and convenience sample	Not reported in manuscript
Method of approach	Email or telephone	p. 4
Sample size	20 interviews with health care worker, prevention worker, coach, psychiatric mental health nurse, psychologist, peer support worker, volunteer, founder of intervention, advisor, coordinator, manager	p. 6, 7
Non-participation	5 did not participate	p. 6
Setting		
Setting of data collection	The interviews professionals were conducted via Microsoft Teams, Skype or telephone, between June and December 2022	p. 7
Presence of non-participants	No	Not reported
Description of sample		p. 6, 7
Data collection		
Interview guide		p. 7
Repeat interviews	No	Not reported in manuscript
Audio/visual recordings	Audio for Skype and telephone, visual recordings for microsoft teams interviews	Not reported in manuscript
Field notes	Yes	Not reported

For peer review only

Duration	The interviews ranged from 40.4 to 85.4 minutes	p. 7
Data saturation	Interviews were conducted until data saturation occured	p. 7
Transcripts returned	N/a	Not reported
**Domain 3: analysis and findings**		
Data analysis		
Number of data coders	2	p. 9
Description of the coding tree		p. 9
Derivation of themes	Themes were derived from data	p. 9
Software	Atlas.TI	p. 9
Participant checking	No	Not reported
Reporting		
Quotations presented	Yes, and each quotation is identified	p. 9-12
Data and findings consistent	Yes	
Clarity of major themes	Yes	p. 9-12, 14-17
Clarity of minor themes	Yes	p. 12-14

For peer review only

Supplementary Material 2

Interviewguide

Background information intervention

- What is the aim of [name intervention]?

- Is there a specific strategy, method or approach used in the interventions? Can you explain?
- Which target group/people can participate in [name intervention]? Why?
- Which target groups/people can nog participate in [name intervention]? Why?

[DEPENDING ON PRACTITIONER INVOLVEMENT OR NOT]

- What is the role of the professional?
- What does the contact between participant and professional look like?
- What expertise, or qualifications, must a professional have to provide [name intervention]? And why?

I am now going to ask you questions about your perspective on the working elements of [name intervention]and how it is provided. I would like to ask you to answer from your own experience as a professional.

Working elements

- What impact do you think [name intervention] has on the actual alcohol consumption of participants aged 55+?
- What impact do you think [name intervention] has on how people aged 55+ think about alcohol consumption?

- In your opinion, what makes [name of intervention] cause [listed outcomes]?
- What do you think are working elements of the intervention?
  For each element, ask the following things:
  o What works well for whom? Can you explain this?
  o To what extent do you think this works well? Can you explain this?
  o How does this work? Can you explain?
  o Why do you think this works well? Can you explain?

Context

- What is the impact of how [the support/name of intervention] is provided? By provided you can think of it being offered [individually/in a group] and [online/offline]?
  o For whom do you think this mode works well? Can you explain this?
  o To what extent do you think this works well? Can you explain?
  o Why does this work well according to you? Can you explain?
  o Why do you think this works well? Can you explain?

- What is the impact of the location where [the support/name of intervention] is provided?
  o For whom do you think this location works well? Can you explain?
  o To what extent do you think this works well? Can you explain?
  o Why does it works well? Can you explain?
  o Why do you think this works well? Can you explain?

Relatives

- To what extent do participants aged 55+ receive support from relatives during [name intervention]?
- o What does this support look like?
- What is the impact of support from relatives? Can you explain?
  o For whom do you think support works well? Can you explain?
  o To what extent do you think this support works well? Can you explain?
  o Why do you think it works well? Can you explain?
  o Why do you think this works well? Can you explain?

- What support would (further) be desirable during participation in [name intervention]? How do you think this could be achieved?

- How do you think relatives experience providing support?
- To what extent do you think relatives need help or support?

**CEMO statements**
I am going to present a few statements. I would like to invite you to respond to these statements.
Practitioner – in-person – individual (A)<br>• An intervention with face to face guidance from a professional can make people (55+) reduce or stop drinking.<br>o Definitely when there is a focus on drinking bheaviour, making people think about their own alcohol use.<br>o Definitely when the professional is very empathetic, making participant and professional work together in finding appropriate help and improving their relationship.
Practitioner – not in-person – individual (B)<br>• An intervention with no face-to-face guidance from a professional can make people (55+) reduce or stop drinking.<br>o Definitely when there is personal contact with feedback.<br>o Definitely when there is online communication with feedback.
Practitioner – in-person – relatives (C)<br>• An intervention with face to face guidance from a professional with relatives' involvement can make people (55+) reduce or stop drinking.<br>o Defenitely when the partner is taught to deal with the behavior of the drinking partner, this can lead to more understanding and support from the non-drinking partner for the drinking partner.
Practitioner – in-person – group component (D)<br>• An intervention with face-to-face guidance from a professional in a group setting can make people (55+) reduce or stop drinking.<br>o Definitely when people are motivated to make lifestyle changes.<br>o Definitely when this is delivered in a workplace setting.
No practitioner – not in-person – individual (E)<br>• An intervention without guidance from a professional can make people (55+) reduce or stop drinking. .<br>o Definitely in web based interventions<br>o Definitely with telephone based interventions.
No practitioner – not in-person – group component (F)<br>• An intervention without guidance from a professional in a group setting can make people (55+) reduce or stop drinking.<br>o Definitely when the intervention focuses on abstinention, which increases awareness of their alcohol consumption
Closing interview *(5 minutes)*
• We are including the following interventions in the study: Moti-55, Ikpas, the alcohol info line, Fris Verder, Healthy Enjoyment, Vitality Days, Maxx, the LessDrinking app and Mirro. To your knowledge, are there any other interventions for older adults (55+)?
• Is there anything else you wild like to add to the interview?
• We would like to publicly share the data and information from these interviews and questionnaire you have completed with other researchers. This will allow this information to be reused for new research in the future.<br><br>• Before we share this data, we will anonymise all information. This means that personal data or data that can be traced back to you as a person such as name, age or other info that reveals your identity will not be shared with them. Such information will be blacked out before the data is made public.<br><br>• Do you give permission to store the anonymised research data for an unlimited period and to make it publicly available to other researchers?<br>You are not required to give permission for this. Without permission, we will not make the data publicly available and it will not be used for follow-up research. Even if you do not give permission for this, your data can still be used for current research.
**Recorder off**
• I would like to thank you for the interview and the time you were willing to spend.

- You will be digitally sent a voucher worth 20 euros.
- Should there be any questions at a later moment, please contact me.
- Also, if you do not have any questions but feel the need to talk after, please contact me.

For peer review only

Supplementary Table 1. Comprehensive program theory

Context	Element of intervention	Mechanism	Outcome
**A. Practitioner – in-person – individual**	(No element, context only)	No misery others (II)<br>Practitioner functions as a big stick (II)<br>Dare to discuss things face to face better than online (IV)	
	1. Paying attention to drinking behavior		
	1.1 Paying attention to drinking behavior		
	1.1.1. Paying attention to drinking behavior	Thinking and awareness	Less or no alcohol use (X)
	1.1.2. Conversation with practitioner about alcohol use (X)		
	1.2 Tracking alcohol use and reflecting on use		
	1.2.1. Reflecting on alcohol use (II, X)	Insight to use (II)<br>Confrontation and reflection (II)	
	1.2.2. Tracking alcohol use (II)	Insight to use (II, III, IV)<br>Confrontation (II)	
	1.2.3. Paying attention to the cause of alcohol use (X)	-<br>Thinking about use/lifestyle (II)	Less or no alcohol use (X)<br>Awareness and insight alcohol use (II)
	1.3. Paying attention to abstinence, coping planning and practicing with abstinence		
	1.3.1. Coping planning abstinence (I, IV, X)		
	1.3.2. Practicing with abstinence	-	Awareness alcohol use (IV)
	1.3.3. Raising awareness of responsible use/abstinence (I)	Awareness alcohol use (I)	
	1.4. Pointing out the risks and consequences of drinking behavior		
	1.4.1. Information about risks and consequences (I, II, IV, X)	Thinking about and recognize indirect consequences in own vulnerability (I, II)	Awareness of effects of alcohol use, less or no use (I, II)
		Shocked by consequences<br>Insight to use (IV)	Awareness of health effects of alcohol use (I)
		Increase risks perception for consequences → Motivation to reduce	Less alcohol use (IV)
	2.Paying attention to lifestyle		
	2.1. Paying attention to meaning of life, and teaching lifestyle changes		
	2.1.1. Attention and tips of meaning of life	Meaning in life	Less or no alcohol use (II)
	2.1.2. Teaching lifestyle changes (II)		
	2.1.3. Attention to different aspects of life (II)		
	3. Communication approaches		
	3.1. Motivational interviewing		
	3.1.1 Motivational interviewing (X, II)	Connect with client (X)<br>-	 Awareness (X)
	3.2. Personalized content and conversation		
	3.2.1. Personalized content and conversation (I, II)		

4. The relationship between the client and practitioner			
4.1. Safety, trust, connection, and not being judged in contact with practitioner			
4.1.1. Client feels comfortable with practitioner (II)			
4.1.2. Low-threshold contact with practitioner (X)	-		Less or no alcohol use
4.1.3. Contact with practitioner feels safe	Opening conversation to discuss drinking behavior (X)		
4.1.4. Client feels accepted	Opening conversation to discuss drinking behavior (X)		
4.1.5. Client feels connection with practitioner	Opening conversation to discuss drinking behavior (X)		
4.1.6. Practitioner has an open attitude and does not judge	Client feels comfortable<br>-		Opening up at follow-up conversation (II)<br>Moderate drinking/awareness (II)
4.1.7. Good therapeutic relationship (X)			
4.1.8. Practitioner is friendly	Client feels comfortable		Openness at follow-up conversation (II)
4.2. Empathic behavior of practitioner			
4.2.1. Practitioner is empathic (I, X)	Client feels understood about alcohol-problems (X)	Client and practitioner collaborate in the identification of additional help, and the relationship between the client and the therapist improves	Less or no alcohol use (X)
	Client and practitioner collaborate in the identification of additional help, and the relationship between the client and the therapist improves (X)		
	Good relationship of trust (X)		
	Client becomes more honest and willing to discuss things (X)		
4.3. Critical, controlling and confronting behavior of practitioner			
4.3.1. Practitioner has active role/guidance (II)	Engagement (II)		
4.3.2 Practitioner makes client reflect critically on client's actions (IV)	-		Less or no alcohol use (X)
4.3.3 Practitioner is critical/strict (II)			
4.3.4 Practitioner sets boundaries (IV)			
4.3.5 Practitioner confronts (II)			
5. Favorable setting			
5.1. Location is nearby home and/or familiar			
5.1.1. Location is nearby home and/or familiar	Low-threshold participation (I, IV)<br>Less effort to go the location		 Increase chances of participation (IV)
5.2. * Pleasant atmosphere			
5.2.1. Meeting with friendly and non-committal atmosphere	Clients can decide for themselves what they will and will not discuss (I)		
*6. * Other*			
6.1. Additional contact			

	6.1.1. Introductory interview	Alignment between practitioner and client		Client wants follow-up interview (II)
	6.1.2. Multiple sessions	More attention for (different) themes and needs (II)		
	6.1.3. Conversation with general practice-based nurse specialist/GP (II)			
	6.2. Other characteristics/input practitioner			
	6.2.1. Practitioner has much knowledge	Thinking about alcohol use		Motivation less alcohol use(X)
	6.2.2. Practitioner shares own experience	Equal contact	Connection between practitioner and client	Client is open for what practitioner has to offer (IV)
**B. Practitioner – not in-person – individual**	(No element, context only)	Practitioner functions as a big stick (X)		
	1. Communication approaches			
	1.1. Contact always possible			
	1.1.1.Always the possibility of contact (I)	Accessible help in difficult situations		Less or no alcohol use, think differently about alcohol use, being open about alcohol use (I, III)
	1.2. Conversation over the phone			
	1.2.1. Telephone conversation (I)	Accessible and flexible (distance, time and anonymity) (I, X)<br>Responding to emotions (I)		
	1.3. Anonymous			
	1.3.1. Anonymous and telephone conversation (I)	Distance between practitioner and client	Client is more likely to accept advice (I)<br>Client speaks and opens up easier (I)	
		Low-threshold (I)<br>Less attention, shame (I)		
		-		Think differently about alcohol (I)
		-		Being open about alcohol use (I)
	1.3.2. No relationship between client and practitioner	Honest instead of socially desirable contact		Think differently about alcohol use (I)<br>Being open about alcohol use (I)
	1.4. Motivational interviewing			
	1.4.1. Motivational interviewing	Engagement		Awareness alcohol use (I, X)
		Open communication and client accept advice from practitioner (I)		
	1.5. Personalized content and conversation			
	1.5.1. Personalized content and conversation (I)	Tailoring to needs and level (I)		Increase knowledge and motivation (I)
		Non-commitment disappears		Less or no alcohol use (III)
		-		Knowledge about where to go when seeking help, learning to cope with alcohol use, increasing motivation for less or no alcohol use (I)
		Pick up emotions (I)	Establish a relationship	Communicate message better to client (I)<br>Make the client think critically (I)

		Possibility of attention other problems (III)<br>Feeling of support		Insight into the cause of the problem (III)<br>Less or no alcohol use (III, X)
	1.6. Personal contact and feedback			
	1.6.1. Personal contact and feedback (X)	Non-commitment disappears (X)		Less or no alcohol use (X)
	1.7. * Other			
	1.7.1. Five-phase model	Open communication and clients accept advice from practitioner (I)		Awareness alcohol use (I)
	1.7.2. Structure, clear expectations and agreements for client (III)	A big stick		Awareness alcohol use (III)
	1.7.3. Regular contact	A big stick	Motivation	Awareness alcohol use, less or no alcohol use (III)
	2. The relationship between the client and practitioner			
	2.1. Practitioner is empathic, supportive and listens			
	2.1.1 Gentle approach with compliments (I)			
	2.1.2 Practitioner does not judge about alcohol behavior, supports and listen (I)			
	2.1.3 Practitioner support and listens (I)			
	2.1..4 Practitioner is empathic	Engagement	Clients accept information from practitioner<br>Open communication	Client will seek help from addiction services (I)
	2.2. * Practitioner takes away the shame with positive approach	Reduce craving for alcohol (I)		
	3. Providing additional help			
	3.1. Personal treatment with practitioner additional to selfhelp			
	3.1.1 Personal treatment with practitioner additional to selfhelp (III, X)	Working independent (III)<br>Keep more attention<br>Provide extra tailoring (III)<br>Possibility of involving relatives (III)<br>Recognition (III)		More likely to persist (X)
	*4. *Paying attention to drinking behavior*			
	4.1. Information about risks and possible help			
	4.1.1. Information about health risks	-		Increase knowledge about alcohol (I)
	4.1.2. Information about alcohol interventions (I)			
**C. Practitioner —in-person— relatives**	*1. Teaching the partner to deal with drinking behavior*			
	1.1 Teaching the partner to deal with drinking behavior			
	1.1.1. Teaching the partner about/dealing with drinking behavior(I, IV, X)	Partner gets more trust in client<br>More understanding and support from the relative for the client (X)		Growing together and taking steps (IV)
	2. Support of relatives			
	2.1 Relative provides support			
	2.1.1.Relatives are deployed to provide support (X)	-		Less or no alcohol use

	*3. * Other*			
	3.1. Relative controls			
	3.1.1. Relative gives or controls information (II)			
	3.2. Partner shares experience and insights with other partners			
	3.2.1 Partner shares experience and insights with other partners	Partner is relaxed and is less controlling		Easier for client to achieve goals (IV)
**D. Practitioner – in-person – group component**	(No element, context only)	Togetherness, connection, recognition (I)<br>Recognition<br>Location gets people outside where addiction takes place (IV)<br>Safety (V)<br>Free to share things by others talking (I)	Sharing 'stick behind the door' experience<br>-	Know how to improve quality of life during abstinence, abstinence (IV)<br>Less or no alcohol use (IV)<br>Achieving goals (IV)
	1. Paying attention to drinking behavior			
	1.1. Pointing out the risks and consequences of drinking behavior			
	1.1.1. Information about risk and consequences (IV, V)	Insight in addiction (IV)		
	1.2. Paying attention to abstinence and practicing with abstinence			
	1.2.1. Attention and tools for (sustained) abstinence	-<br>Less alcohol use	Mood improves	Less or no alcohol use (IV)<br>People become more active and anxiety symptoms will reduce (IV)
	1.2.2. Practicing with new or tense situations	Learning to tolerate discomfort in life (IV)<br>Gain experience (IV)		
	1.3. *Discuss statements on alcohol use in groups			
	1.3.1. Discuss statements in groups	Thinking about alcohol use (I)<br>Openness about alcohol use (I)		
	2. Paying attention to lifestyle			
	2.1. ** Motivating to change lifestyle			
	2.2. Attention and tips regarding meaning of life, lifestyle and problems when aging			
	2.2.1 Attention for meaning of life and life stage themes (IV, X)	Motivation to go to the next session (IV)<br>Better coping with cravings for alcohol (IV)<br>Motivation for change		Abstinence, know how to improve quality of life during abstinence (IV)
	2.1.2. Attention for lifestyle or motivate lifestyle change (X)	-		Less alcohol use (X)

2.3. **Motivating to change lifestyle delivered in a workplace setting			
2.4. In a workplace setting and paying attention to prevention and lifestyle			
2.4.1. At work (X)	Increase awareness (X)		
2.4.2. At work with attention for prevention (X)			
2.4.3. At work with attention for lifestyle (X)			
2.4.4. * At work, openness about alcohol use	Negative consequences (X)		
	Support environment (X)		
	-		Less alcohol use (X)
2.4.5. * At work, people join national campaign (X)			
3. Communication approaches			
3.1. Motivational interviewing			
3.1.1. Motivational interviewing	-		Less or no alcohol use, awareness (X, IV)
3.2. Personalized content and conversation			
3.2.1. Personalized content and conversation (IV, X)			
3.2.2. * Customized CBT treatment (IV)			
3.3. * Other approaches			
3.3.1. Change way of thinking from negative to positive (IV)	Rethinking and self-pity reduces	Getting stronger to prevent relapse	Less or no alcohol use (IV)
3.3.2. Overcoming shame			Less or no alcohol use (III)
3.3.3. Mindfulness and running therapy (V)	Feeling signals in body and recognize (V)		
3.3.4. CGT focusing on substance use (IV)	-		Less or no alcohol use
3.3.5. Structure in days	Having rhythm and purpose to go somewhere (V)		
3.3.6. Variance of working methods (IV)			
4. The relationship between the client and peers			
4.1.Contact with peers			
4.1.1. Contact with peers (IV, IV, IV, X)	Being seen and feel supported (IV)		
	Connection (IV)		
4.1.2. Group composition like-minded peers (IV, IV)	Recognition and connection (IV,V, X)		
4.2. Closed group and maximized group size			
4.2.1.Maximized group size	Creating safety		Preventing drop out (IV)
	More attention per client (I, IV)		
4.2.2.Closed group	Improves atmosphere (IV)		
	Ongoing process (IV)		
	Safety	Daring to open up (IV)	
4.3. Engagement, understanding and support towards peers			
4.3.1. Engagement and understanding each other (IV)	Connection (IV)		
	Safety	Talking about problems (IV)	Less or no alcohol use (IV)

For peer review only

	Togetherness, being able to ask for help from each other (IV)		
4.3.2. Support each other (V)			
4.3.3. People feeling heard in group	Less alcohol use	Mood improves	People become more active and anxiety symptoms will reduce (IV)
4.4. Addressing peers			
4.4.1. Addressing each other, reflect critically on your own actions (II, V)	Learning and getting insights		Less or no alcohol use (III, IV)
4.5. Sharing experiences and tips with peers			
4.5.1. Sharing experiences and tips (V, X)	Hopeful for own process (IV, V)		
	Openness about alcohol use (IV)		
	Recognition and acknowledgement (IV)		
	Putting own situation in perspective (IV)		
	Feeling the impact of addiction (IV)		
4.6. * Making agreements with peers			
4.6.1. Making agreements among themselves in group	A big stick (IV)		
5. The relationship between the client and practitioner			
5.1. Open attitude, not being judged and accessible contact with practitioner			
5.1.1. Practitioner is unbiased, open and not moralizing (V)			
5.1.2. Accessible contact practitioner	Experience support		Less relapse in alcohol use (V)
5.2. * Other			
5.2.1. Practitioner has the same age of clients (IV)	Recognition and click (IV)		
5.2.2. Presence two practitioners (IV)			
5.2.3. Interactive contact	Holding attention (I)		
5.2.3. Guest speakers focusing on different lifestyle themes (IV)	-		Abstinence, know how to improve quality of life during abstinence (IV)
6. Other activities			
6.1. Having lunch after every session			
6.1.1. After every session have a lunch together (IV, V)	Connection (IV)		Less or no alcohol use (IV)
	Atmosphere in the group (IV)		
	Unforced atmosphere		Discuss topics in a low-threshold way that are more difficult during a session (IV)
			Safe setting (IV)
	Relaxation and getting to know each other better (IV)		
	Insight of simplicity nutritious meal (IV)		
6.2. * Activities			
6.2.1. Social activities (V)	Daytime activities	Reduce loneliness (IV)	

	6.2.2. Outdoor intervention in group walking with app	Stimulate to move or exercise		Better coping with cravings for alcohol (IV)
	7. Favorable setting			
	7.1. Pleasant and relaxed atmosphere			
	7.1.1. Meeting with friendly and non-committal atmosphere	Clients can decide for themselves what they will and will not discuss (I)		
	7.1.2. Besides serious atmosphere, also relaxed atmosphere with humor (IV)			
	7.2. Location is nearby home and/or familiar			
	7.2.1. Location is nearby home and/or familiar	Low-threshold participation (I, IV)		
		Less effort to get to location	Increase chance of participation (IV)	
	7.3. * Location is not admission clinic			
	7.3.1. Location is not admission clinic	People are not remembered by their admission (V)		
**E. No practitioner – not in-person - individual**	*1. Paying attention to drinking behavior*			
	1.1. Tracking alcohol use			
	1.1.1.Monitoring alcohol use	Keep track of progress		Less alcohol use (III)
	1.1.2. Tracking alcohol use in app/telephone	Insight into alcohol use(X)		Less or no alcohol use (X)
	1.1.3. * Information and tools relapse and dealing with difficult moments	Prevent relapse	Handle difficult moments better and consciously (III)	
	1.2. Abruptly quit alcohol use			
	1.2.1. Abruptly quit alcohol use for a temporary period (III)	Learning experience that can be perceived as positive (II)	High self-efficacy and attitude change towards alcohol use (III)	High probability that people will use less or no alcohol use later (again) (III)
		Awareness behavioral change (III)		
	1.3. Pointing out the risks and consequences of drinking behavior			
	1.3.1. Paying attention to risks of alcohol use at age	Get people thinking (III)		
	1.4. * Receiving feedback			
	1.4.1. Receiving feedback	Motivation		Less alcohol use (III)
	1.4.2. Positive feedback	Motivation		Less alcohol use (III)
	2. Using tools			
	2.1. ** Web based interventions			
	2.2. Online self-help tool			
	2.2.1.App	No waiting list so quick participation (III)<br>Getting started with themes in a low-threshold way (X)		
	2.2.2. App that is simple and clear	Accessible (III)		
	2.2.3. Online self-help tool /program /module	Own direction implementation (III)<br>Low-threshold(III)		Less or no alcohol use (X)
		-		Awareness/ Less or no alcohol use/ Step towards treatment (IV)

	2.2.4. Short online module with playful approach (III)	Awareness		Small changes alcohol use (III)
	2.3. Via telephone			
	2.3.1. Via telephone	Saving time/cost (X)		
	2.4. Regular newsletter			
	2.4.1. Regular newsletter (III)	Being actively engaged<br>Recognition support (III)<br>-	Support (III)	 <br>Being motived to use less or no alcohol use for longer periods of time (III)
	2.5. * Other			
	2.5.1. Webinar with peer chatbox	Personal contact what serves as social support (III)		
	3. The relationship between the client and peers			
	3.1. Joining with others, contact with peers			
	3.1.1. Additional contact with peers (III)			
	3.1.2. Joining with others (III)	Feeling of togetherness (III)<br>Feeling of recognition (III)<br>Social support (III)		Increase success of intervention (III)
	4. Favorable setting			
	4.1. Location independent participation			
	4.1.1. Location-independent/participation possible from home (III)	Accessible and low-threshold (III)		
	5. Additional help			
	5.1. Personalized content and help additional to self-help			
	5.1.1. Additional personal coaching at self-help interventions (III)			
	5.1.2. Additional physical tools for client (III)	Feeling of physical participation at home (III)		
	5.1.3. Extra options for support (III)	Feeling of personal, voluntary and non-committal participation		Less or no alcohol use for one month (III)
	6. * Other			
	6.1. Attention other themes			
	6.1.1. Attention to activity spending	Get people thinking (III)		
	6.1.2. Attention for difficult aspects in life	Get people thinking (III)		
	6.1.3. Commitment with yourself by signing up (III)	Motivation		Increase success of intervention (III)
	6.1.4. Non commitment participation	Low-threshold (III)<br><br>Extra motivation (III)		Makes less or no alcohol use pleasant, step up to intensive help (III)
	6.1.5. Attention to positive experiences	Increase self-efficacy (III)		
	6.1.6. Setting goals	-		Less alcohol use (III)
**F. No practitioner – not in-person –**	(No element, context only)	Motivation and not being alone		Less or no alcohol use (X)
	1. Paying attention to drinking behavior			
	1.1. **Intervention to abstinent people			

**group component**	1.2. Focus on abstinence and paying attention to withdrawal		
	1.2.1. Focus on abstinence, with attention to withdrawal (X)		
	1.2.2. Focus on abstinence (X)		
	2. Self-help groups		
	2.1. AA and self-help group		
	2.1.1. Aa/self-help group (X)	-	Less or no alcohol use (II)
	3. The relationship between the client and peers		
	3.1. Contact with peers		
	3.1.1. Peer contact (IV, X)	Recognition (X)	
	3.2. Sharing experiences and tips with peers		
	3.2.1. Sharing experiences and tips with peers (III)	Hope and connection (X)<br>Persuade to participate (X)<br>Stimulate (X)	
	3.3. * Other		
	3.3.1. Composition of people with same level/serious issues (III)		
	3.3.2. Making agreements and setting goals in group	-	Less or no alcohol use (X)
	3.3.3. Helping each other (X)		
	3.3.4. Online forum	Social support	Temporary stop of alcohol use (III)
	4. * Paying attention to other aspects		
	4.1. Broader focus than abstinence		
	4.1.1. Broader focus than only abstinence	Thinking about what is important	Less or no alcohol use (X)

*= Program theories that were not included in Table 3. ** Initial program theories that were refined or not confirmed.
I = People with all types of alcohol use patterns, II = People with early stage problematic alcohol use; III = People with all types of alcohol use patterns, except for (heavy) problematic alcohol use or alcohol addiction; IV = People with problematic alcohol use and alcohol addiction; V = People who are in recovery from addiction, X = No target group mentioned because these elements were derived from CEMO configuration that we presented as statements during the interviews.

For peer review only - http://bmjopen.bmj.com/site/about/guidelines.xhtml

BMJ Open

Professionals' perspectives on interventions to reduce problematic alcohol use in older adults: A realist evaluation of working elements.

Journal:	*BMJ Open*
Manuscript ID	bmjopen-2023-077851.R2
Article Type:	Original research
Date Submitted by the Author:	15-Mar-2024
Complete List of Authors:	van den Bulck, Fieke; Tilburg University Tranzo Scientific Centre for Care and Welfare, Tranzo<br>Knijff, Rikste; Tilburg University Tranzo Scientific Centre for Care and Welfare, Tranzo<br>Crutzen, Rik; School for Public Health and Primary Care (CAPHRI), Department of Health Promotion<br>van de Mheen, D; Tilburg University,<br>Bovens, Rob; Tilburg University Tranzo Scientific Centre for Care and Welfare, Tranzo<br>Stutterheim, Sarah; Maastricht University, Department of Work and Social Psychology<br>Van de Goor, Ien ; Tilburg University Tranzo Scientific Centre for Care and Welfare<br>Rozema, A; Tilburg University,
**Primary Subject Heading**:	Addiction
Secondary Subject Heading:	Qualitative research
Keywords:	Aged, Health Services for the Aged, QUALITATIVE RESEARCH

SCHOLARONE™
Manuscripts

I, the Submitting Author has the right to grant and does grant on behalf of all authors of the Work (as defined in the below author licence), an exclusive licence and/or a non-exclusive licence for contributions from authors who are: i) UK Crown employees; ii) where BMJ has agreed a CC-BY licence shall apply, and/or iii) in accordance with the terms applicable for US Federal Government officers or employees acting as part of their official duties; on a worldwide, perpetual, irrevocable, royalty-free basis to BMJ Publishing Group Ltd ("BMJ") its licensees and where the relevant Journal is co-owned by BMJ to the co-owners of the Journal, to publish the Work in this journal and any other BMJ products and to exploit all rights, as set out in our licence.

The Submitting Author accepts and understands that any supply made under these terms is made by BMJ to the Submitting Author unless you are acting as an employee on behalf of your employer or a postgraduate student of an affiliated institution which is paying any applicable article publishing charge ("APC") for Open Access articles. Where the Submitting Author wishes to make the Work available on an Open Access basis (and intends to pay the relevant APC), the terms of reuse of such Open Access shall be governed by a Creative Commons licence – details of these licences and which Creative Commons licence will apply to this Work are set out in our licence referred to above.

Other than as permitted in any relevant BMJ Author's Self Archiving Policies, I confirm this Work has not been accepted for publication elsewhere, is not being considered for publication elsewhere and does not duplicate material already published. I confirm all authors consent to publication of this Work and authorise the granting of this licence.

For peer review only

Professionals' perspectives on interventions to reduce problematic alcohol use in older adults: A realist evaluation of working elements

F.A.E. van den Bulck[1], R. Knijff[1], R. Crutzen[2], D. van de Mheen[1], R.H.L.M. Bovens[1], S.E. Stutterheim[2], I. van de Goor[1], & A.D. Rozema[1]

[1] Tranzo Scientific Center for Care and Wellbeing, School of Social and Behavioral Sciences, Tilburg University, the Netherlands.

[2] Department of Health Promotion, Care and Public Health Research Institute (CAPHRI), Maastricht University, the Netherlands.

Affiliation addresses

[1] Professor Cobbenhagenlaan 125, Postbus 90153, 5000 LE Tilburg, The Netherlands

[2] Peter Debyeplein 1, 6229 HA Maastricht, The Netherlands

Corresponding author

F.A.E. van den Bulck

Email: f.a.e.vdnbulck@tilburguniversity.edu

Address: Professor Cobbenhagenlaan 125, Postbus 90153, 5000 LE Tilburg, The Netherlands

Abstract

Objectives

This study set out to understand how (which elements), in which context, and why (which mechanisms) interventions are successful in reducing (problematic) alcohol use among older adults, from the perspective of professionals providing these interventions.

Design

Guided by a realist evaluation approach, an existing initial program theory (IPT) on working elements in alcohol interventions was evaluated by conducting semi-structured interviews with professionals.

Setting and participants

For peer review only

These professionals (N=20) provide interventions across several contexts: with or without practitioner involvement; in-person or not; and in an individual or group setting. Data were coded and links between contexts, elements, mechanisms, and outcomes were sought for to confirm, refute, or refine the IPT.

Results

From the perspective of professionals, there are several general working elements in interventions for older adults: 1) *pointing out risks and consequences of drinking behavior*; 2) *paying attention to abstinence*; 3) *promoting contact with peers*; 4) *providing personalized content*; and 5) *providing support*. We also found context-specific working elements: 1) *providing personalized conversations* and *motivational interviewing* with practitioners; 2) *ensuring safety, trust and a sense of connection* and *a location nearby home or a location that people are familiar with* in person, and 3) *sharing experiences and tips* in group interventions. Furthermore, the mechanisms *awareness* and *accessible and low-threshold participation* were important contributors to positive intervention outcomes.

Conclusion

In addition to the IPT, our findings emphasize the need for social contact and support, personalized content, and strong relationships (both between client and practitioner, and client and peers) in interventions for older adults.

Strengths and limitations of this study

- Using a realist evaluation provides in-depth insights in interventions because it can explain why interventions are successful or not.
- Data were collected through interviews with a variety of professionals across eleven different alcohol interventions.
- Habitation bias and acquiescence bias could have occurred in data collection, which may influence the confirmed IPT.

For peer review only - http://bmjopen.bmj.com/site/about/guidelines.xhtml

INTRODUCTION

In recent decades, alcohol use has increased, particularly in older adults (1-5). This has been accompanied by increased problematic alcohol use including high-risk drinking, binge drinking, and alcohol use disorders (6-8). Moreover, this change in drinking patterns has been associated with increased alcohol-related health problems in older adults due to biological changes in metabolism, body fat, body water, and the reduced ability of the liver to process alcohol (9, 10), and due to the combination of alcohol with (increasing amounts of) medication when aging (11).

This increase in problematic alcohol use among older adults emphasizes the need for effective interventions. There are several existing approaches and specific interventions to reduce alcohol use, such as screening, brief Intervention, and referral to treatment (SBIRT), cognitive behavioral therapy (CBT) or motivational enhancement therapy, that are considered to be appropriate for older adults (12-16). However, most interventions to reduce (problematic) alcohol use in which older adults participate are designed for the general adult population; there is no consensus yet on what works best for specifically older adults (17, 18). Nonetheless, two systematic reviews found that effective intervention components to reduce alcohol use among older adults are personalized feedback, advice on (alcohol) behaviors, educational materials, and counseling (19, 20).

Realist evaluation (RE) work from the assumption that interventions and their elements (E) work differently in different contexts (C). The context refers to the circumstances in which interventions operate —e.g., an online context in which an intervention is provided. Interventions may be successful in some contexts and not in others because the underlying processes through which the interventions bring change, called the mechanisms (M), are triggered to a different extent and lead to different intervention outcomes (O). Therefore, the interaction between the intervention elements (E), contexts (C), and mechanisms (M) plays an important role in shaping the intervention outcomes (O). The strength of RE is that it offers a means to unpack the relationships between these contexts, elements, mechanisms, and outcomes. This is called the context-element-mechanism-outcome (CEMO) configuration (21). RE starts with formulating an initial program theory (IPT) based on the CEMOs founded in literature. The IPT then needs to be confirmed, refuted, or refined using data that seeks to explain how the intervention works in real life contexts from the perspectives of those involved in its operation. Finally, based on the new data, the program theory might be refined.

For peer review only

A systematic review which included 61 studies – using RE – focused on understanding how, in what context, and why interventions are successful in preventing or reducing (problematic) alcohol consumption among older adults (22). This review explored what works for individuals aged 18 years and older (which includes older adults), and for older adults specifically, and created an IPT based on the working elements found across six different contexts.

Firstly, in interventions that were delivered in a context with practitioner involvement, in person, and to one individual (instead of a group), the working elements of *paying attention to drinking behavior* and *the relationship between the client and practitioner* were identified. Secondly, in interventions that were delivered in a context with practitioner involvement, not in person and individual, *personal contact and feedback* and *online communication and feedback* were identified. In interventions that were delivered in a context of practitioner involvement, in person and with involvement of relatives, *status of the relationship* and *teaching the partner to deal with drinking behavior* were identified. In interventions that were delivered in a context of practitioner involvement, in person and in group settings, *motivating to change lifestyle* (delivered in a workplace setting) was identified. In interventions that were delivered in an individual context without practitioner involvement, *web-based* and *telephone-based* interventions were working elements. Finally, in interventions that were delivered in a context without a practitioner, not in person and in a group setting, *focus on abstinence* was identified.

Overall, the results of the review showed three general working elements: 1) providing information about the consequences of alcohol consumption; 2) personalized feedback about drinking behavior; and 3) being in contact with others and communicating with them about (alcohol) problems (22). Of the 61 studies, only three evaluated interventions for older adults, and only the first and second general working elements were found in these studies (23-25).

Evidently, in light of the small number of studies conducted specifically among older adults, more research on what works for older people is needed. The current study builds on the previous review performed using RE.

This study sets out to confirm, refute, or refine the IPT, consisting of the working elements, mechanisms and outcomes across six different contexts, as identified by Boumans et al (22). More specifically, the objective of this study is to understand from the perspective of professionals providing these interventions how (which elements), in what context, and why (which mechanisms) interventions are successful in reducing (problematic) alcohol use among older adults. The perspective of

For peer review only - http://bmjopen.bmj.com/site/about/guidelines.xhtml

professionals was considered important as they have extensive experience in addressing problematic alcohol use among older adults and a comprehensive understanding of what works and why . Based on the perspectives of the professionals, CEMOs of the IPT will be confirmed, refuted, or refined.

METHOD

Study Context and Design

In this qualitative study, we conducted semi-structured interviews with professionals providing interventions to older adults. This study is part of a larger qualitative research project investigating the perspectives of clients and close relatives of clients. Here, we focus on the professionals' perspectives. The larger study was approved by the Ethics Review Board at Tilburg University (RP508) and the COREQ guidelines were followed in reporting and design (26). See Supplementary Material 1.

Sampling and Recruitment

In the current study, we set out to recruit and interview professionals providing various interventions across different organizations throughout the Netherlands.

Firstly, we inventoried which interventions were used in practice by searching the Dutch National Database Centre for Healthy Living (*Centrum Gezond Leven*) and consulting the national working groups Elderly in the Dutch Partnership for Early Detection of Alcohol Problems (*Samenwerkingsverband Vroegsignalering Alcoholproblematiek*) and Alcohol and Elderly of The Dutch Addiction Association (*Verslavingskunde Nederland*). These two working groups comprise professionals from various organizations providing different interventions.

Secondly, based on this inventory, we included interventions if they 1) focused on universal, selective or indicated prevention; 2) were provided in an individual and/or group setting with or without relative involvement; 3) were provided face to face, online, and/or via telephone; and 4) were provided in the Netherlands. Interventions were excluded if they 1) were not primarily aimed at lifestyle change; 2) were provided in a clinical setting; 3) were group-specific interventions (e.g., for veterans or athletes); or 4) focused on general health and lifestyle improvements without explicit mention of alcohol use.

Based on these selection criteria, the following Dutch alcohol interventions were included: Alcohol Information Line (*Alcohol Infolijn*), CBT for Substance Use (*CGT bij middelengebruik*), Enjoy Healthy (*Gezond Genieten*), Fresh Onwards, (*Fris Verder*), Maxx, Mirro, Moti-55, NoThanks (*IkPas*), Recovery-Guided Personal Program (*Herstel Ondersteunend Persoonlijk Programma*), Vitality Days

For peer review only - http://bmjopen.bmj.com/site/about/guidelines.xhtml

(Vitaliteitsdagen). Snowball sampling among professionals helped us identify one other intervention: DrinkLess (*MinderDrinken*)

Thirdly, we approached professionals in the two working groups working with these interventions or we approached professionals at organizations working with the interventions and asked them to connect us with professionals who were involved in providing the interventions. A convenience sampling technique was used to recruit the professionals. Professionals who were interested received information about the study via email and some also received information by telephone. They all received an information letter and informed consent form. Subsequently, they were invited for an interview.

In total, we contacted 25 professionals, of whom 20 participated. The remaining five did not respond to our emails. Participants' characteristics are shown in Table 1. Characteristics of included interventions are shown in Table 2.

Table 1. Participants' characteristics

Baseline characteristic	Participants
	n = 20
Gender	
Woman	15
Man	5
Age (years)	
25-39	6
40-54	4
55-69	7
≥ 70	3
Experience with the intervention (in years)	
<1	2*
1	3
2	4
3	2
4	4
5	3
6	1
7	-
8	-
9	1
Role**	
Health care worker, prevention worker, coach, psychiatric mental health nurse or psychologist	13
Peer support worker	1
Volunteer	3

For peer review only - http://bmjopen.bmj.com/site/about/guidelines.xhtml

Founder of intervention	3
Advisor, coordinator, manager	3
Intervention	
Alcohol Information Line	3
CBT for Substance Use	2
DrinkLess	1
Enjoy Healthy	1
Fresh Onward	4
Maxx	1
Mirro	1
Moti-55	2
NoThanks	2
Recovery Guided Personal Program	1
Vitality Days	2

* One professional was only involved in the development of the intervention.
** Some professionals had multiple roles in the same interventions—e.g. working as a coach and coordinator.

Data Collection

Based on the participants preferences, online (80%) and telephone (20%) interviews were conducted between June and December 2022, each lasting an average of 59.3 minutes (ranging from 40.4 to 85.4 minutes). Demographic variables including age, gender, and some questions about the intervention were asked prior to the interviews. All interviews were preceded by obtaining oral informed consent. We used an interview guide that measured the perceived outcomes of the interventions, the working elements and mechanisms related to the outcome(s), and the influence of the setting in which the interventions were provided. At the end of the interview guide, we presented the previous found CEMO configurations of the IPT of the review of Boumans et al. (22); we invited participants to confirm, refute, or refine the CEMOs and asked if they could comment on the CEMOs. (See interview guide, Supplementary Material 2.) Interviews were conducted until data saturation occurred.

Patient and public involvement statement

None.

For peer review only - http://bmjopen.bmj.com/site/about/guidelines.xhtml

Table 2. Intervention characteristics

Intervention	Contact in intervention with			Setting		Sessions	Target group intervention	
	Professional	Peers	Relatives	Online, telephone	Face to face		Client's alcohol use **	Older adults (55+ or 65+)
Alcohol Information line	X			X		≥1	I	
CBT for Substance Use	X	X	X*		X	>1	IV	X
DrinkLess	X*			X		N/a	III	
Enjoy Healthy	X	X			X	1	I	X
Fresh Onwards	X	X	X*		X	>1	IV	X
Maxx				X		N/a	III	
Mirro				X		N/a	III	
Moti-55	X		X*		X	>1	II	X
NoThanks	X*			X		N/a	III	
Recovery Guided Personal Program	X		X*		X	>1	V	
Vitality Days	X	X*	X*		X	1	I	X

* = The involvement of this person in the intervention is optional and according on the client's preference.

** I = People with all types of alcohol use patterns; II = people with early stage problematic alcohol use; III = people with all types of alcohol use patterns, except for (heavy) problematic alcohol use or alcohol addiction; IV = people with problematic alcohol use and alcohol addiction; V = people who are in recovery from addiction.

For peer review only

Data analysis

Professional transcriptionists transcribed the interviews verbatim. Based on the IPT, FvdB drafted a code tree including CEMO configurations. Researchers FvdB and RK independently and, in parallel, coded six of the 20 interviews based on the RE approach, which means that links between contexts, elements, mechanisms, outcomes were sought within the data. After the first transcript was coded by FvdB and RK, FvdB cross-checked both their configurations and codes. FvdB and RK iteratively discussed differences in configurations or coding, and, where relevant, refinements were made. These steps were repeated for all of the six initial interviews, at which time coding was fully aligned. Thereafter, FvdB coded the remaining interviews independently. When in doubt about how best to code sections of the data, FvdB consulted with RK and ADR. Additionally, to improve the quality of the coding further, RK, RC, RB, IvdG, SS, and ADR reviewed the formed codes and CEMOs iteratively and provided feedback. Codes were then adjusted if necessary and some new codes were added. Data were analyzed using Atlas.TI.

RESULTS

The results are categorized according to the context in which the interventions are delivered: 1) involvement or lack of involvement of a practitioner who provides the intervention directly to the participant; 2) provision of the intervention in person or not; and 3) individual treatment, group treatment or treatment with relatives' involvement. We found data across six combinations of contexts to which the IPT conformed. Below, we describe the working intervention elements (E). Table 3 provides an overview of the key working elements, mechanisms, and outcomes, mentioned by more than one participant. Table 4 provides an overview of the summarized findings of the program theory. For an even more comprehensive table including all CEMOs, even those mentioned by just one respondent, see Supplementary Table 1.

A. Practitioner—in-person—individual

Professionals confirmed that (1) *paying attention to drinking behavior* (E) is important in interventions to reduce alcohol use (O) because it could stimulate clients to start thinking about their alcohol use (M) and create awareness of their use (M).

Alcohol is, of course something that has a risk, can cause health damage. People think about that. 'Do I want to do that to myself? I'm getting older, I'm getting more forgetful.' So they start to think a lot more about what they are doing to themselves because of alcohol use. If you actually tell them that as

For peer review only - http://bmjopen.bmj.com/site/about/guidelines.xhtml

> *a professional, then people really think twice about it: 'Wait a minute, I still want to enjoy my life and I want to age healthily'.* [R9, prevention worker]

They also emphasized the importance of (2) *paying attention to lifestyle* (E) and (3) using specific *communication approaches* (E), such as motivational interviewing. The professionals confirmed that (4) *the relationship between client and practitioner* (E) is important because it could trigger open communication (M), and collaboration between client and practitioner that improves the relationship between practitioner and client (M). Finally, they mentioned that a (5) *favorable setting* (E) in which the intervention is provided is important—i.e., a location that is nearby, because it contributes to low threshold participation (M).

B. Practitioner—not in-person—individual

Professionals confirmed that personal contact combined with feedback reduced alcohol use (O). In addition, they mentioned several other (1) *communication approaches* (E), such as motivational interviewing, personalized contact, and anonymous participation. These approaches triggered different mechanisms—i.e., tailoring (M), and low-threshold and flexible participation (M).

> *And certainly with older people because they often already have certain habits for years or perhaps have a bad marriage or a very stressful job. So those life-stage issues, you can sort those out in personal treatment.* [R3, psychologist and advisor]

> *It also makes it low threshold for people to call. We often have people on the phone who admit for the first time at all that something is going on. […] For example, people often call in their car and I think that is also a nice anonymous place where no one, no family member, a colleague, can see who they are calling. […] People can choose when and where they have this conversation, so they can choose a time and location that they prefer.* [R4, health care worker]

The professionals added that (2) *the relationship between client and practitioner* (E) is important—e.g., when the professional is empathic, supportive and listens to the client—because this could trigger engagement (M), followed by open communication where the client accepts advice or information from the practitioner (M). Finally, they mentioned that (3) *providing additional help* (E) can be important because it could provide extra attention (M) and tailoring (M).

C. Practitioner—in-person—relatives

For peer review only - http://bmjopen.bmj.com/site/about/guidelines.xhtml

Professionals confirmed that a partner is more understanding and supportive of the client (M) when (1) *the partner is taught to deal with the drinking behavior of the client* (E), and that (2) *support by relatives* (E) can lead to alcohol reduction (O) among clients.

> *It is important for them to understand how addiction works, that it's not solved at once and that it is actually a disease. […] . So, the relative regains a bit more confidence and yes, this often creates space in which they can grow together and take steps. On the one hand, this strengthens the client and, on the other hand, it gives a relative more confidence that it can be done.* [R16, peer support worker]

D. Practitioner—in-person—group component

Professionals mentioned that (1) *paying attention to drinking behavior* (E) is important because it contributes to a reduction in alcohol use (O). They confirmed that (2) *paying attention to lifestyle* (E) was also a working element in this context because it motivates people to participate in the intervention and to change (M), which ultimately contributes to reduced alcohol use (O). In addition, they mentioned that (3) *communication approaches* (E), including motivational interviewing, and personalized contact and conversations were important. Professionals also mentioned that (4) *the relationship between client and peers* (E) plays an important role in interventions because it could provide recognition, connection, support, hope, safety, and new insights (M) for the client.

> *There are often a lot of negativities in the family atmosphere and I think in a group with peers it is accepted that people have a problem and they start looking to get it under control. So, there is engagement, an open and accepting attitude that makes people feel safe, and I think that is very important in a group, that people feel very safe to talk about it without feeling judged.* [R13, MH psychologist]

Again, (5) *the relationship between the client and practitioner* (E)—for example when the professional has an open attitude and does not judge the client—was important in interventions according to the professionals. Providing (6) *other activities* (E) such as eating after a joint session was helpful because these contribute to making a connection (M) and a pleasant atmosphere (M).

Finally, (7) a *favorable setting* was important because it contributed to low threshold participation.

For peer review only - http://bmjopen.bmj.com/site/about/guidelines.xhtml

It is often a very high threshold for people to go to an addiction center. So, I think that this kind of treatment could also be given in a community center, or a nursing home, or at the GP in a small group, or something. [R14, psychiatric mental health nurse]

E. No practitioner—not in-person—individual

Professionals mentioned that (1) *paying attention to drinking behavior* (E) contributes to reductions in alcohol use (O). Also (2) *using tools* (E), such as an online self-help tool, was considered beneficial in this setting because it enables low threshold participation (M) for participants and allowed them to be actively involved (M). Again, (3) *the relationship between client and peers* (E) was important because it provided clients with fellowship, recognition and support (M).

I think that for this target group it is very welcome that, additionally in an online setting, they can do it alone or with a friend, or with a group where you meet once per week or every two weeks. [RP6, founder of intervention and manager]

Also, (4) *a favorable setting* (E) was important because it could make participation low threshold (M). Finally, (5) *additional help* (E)—i.e., providing personalized content and help additional to self-help— was also seen as effective.

F. No practitioner—not in-person—group component

Professionals mentioned that (1) *paying attention to drinking behavior* (E), (2) *self-help groups* (E), and (3) *the relationship between the client and peers* (E) were all important in interventions in this context. No mechanisms were found that were mentioned by more than one professional.

Table 3. The program theory

Context (C)	Element of intervention (E)	Mechanism (M)	Outcome (O)	Target group**	IPT ***
A. Practitioner – in-person – individual	1. *Paying attention to drinking behavior*				
	1.1. *Paying attention to drinking behavior	Think and act differently about alcohol use	Less or no alcohol use	X	✓
	1.2. Tracking alcohol use and reflecting on use	a. Creating insight into use	Less or no alcohol use	II, III, IV, X	+
		b. Thinking about alcohol use	Awareness		
	1.3. Paying attention to abstinence, coping planning, and practicing with abstinence	Raising awareness of alcohol use		I, IV, X	+
	1.4. Pointing out the risks and consequences of drinking behavior	Thinking about indirect consequences of use	Awareness, less or no alcohol use	I, II, IV	+
	2. *Paying attention to lifestyle*				
	2.1. Paying attention to meaning of life and teaching lifestyle changes		Less or no alcohol use	II	+
	3. *Communication approaches*				
	3.1. Motivational interviewing			II, X	+
	3.2. Personalized content and conversation			I, II	+
	4. *The relationship between the client and*				

For peer review only - http://bmjopen.bmj.com/site/about/guidelines.xhtml

	practitioner				
	4.1. Safety, trust, connection, and not being judged in contact with professional	Having open communication	Less alcohol use	II, X	+
	4.2. *Empathic behavior of practitioner	Collaborating in the identification of help and needs, and improving the relationship between client and practitioner	Less or no alcohol use	I, X	✓
	4.3. Critical, controlling, and confronting behavior of practitioner			II, IV, X	+
	5. *Favorable setting*				
	5.1. Location is nearby home and/or familiar	Low-threshold participation		I, IV	+
B. Practitioner – not in person – individual	1. *Communication approaches*				
	1.1. Contact always possible	Accessible help in difficult situations		I, III	+
	1.2. Conversation over the phone	Accessible and flexible participation		I, X	+
	1.3. Anonymous	a Distance in contact, making client more likely to accept advice<br>b. Low threshold<br>c. Honest instead of socially desirable contact	Think differently about alcohol use	I	+
	1.4. Motivational interviewing		Awareness	I, X	+
	1.5. Personalized content and conversation	a. Tailoring		I, III	+
		b. Non-commitment disappears	Less or no alcohol use	I, III	+
		c. Emotional support		I, III, X	+
	1.6. *Personal contact and feedback		Less or no alcohol use	X	✓
	1.7. *Online communication and feedback		Less or no alcohol use		-
	2. *The relationship between the client and practitioner*				
	2.1. Practitioner is empathic, supportive, and listens	Engaging, open communication, and client accepts advice or information from practitioner		I	+
	3. *Providing additional help*				
	3.1. Personal treatment with practitioner additional to self-help	Extra attention, tailoring		III, X	+
C . Practitioner—in-person—relatives	1 *Teaching the partner to deal with drinking behavior*				
	1.1. *Teaching the partner to deal with drinking behavior	More understanding and support from the relative for the client		I, IV, X	✓
	2. *Support of relatives*				
	2.1. Relative provides support		Less or no alcohol use	X	+
D. Practitioner—in-person—group component	1. *Paying attention to drinking behavior*				
	1.1. Pointing out the risks and consequences of drinking behavior			IV, V	+
	1.2. Paying attention to abstinence and practicing with abstinence		Less or no alcohol use	IV	+
	2. *Paying attention to lifestyle*				
	2.1. *Motivating to change lifestyle				~
	2.2. Attention and tips regarding meaning of life, lifestyle, and problems when aging	Motivation for participation and change	Less or no alcohol use, know how to improve life quality during abstinence	IV, X	+
	2.3. *Motivation to change lifestyle delivered in a workplace setting				~
	2.4. In a workplace setting and paying attention to prevention and lifestyle			X	+
	3. *Communication approaches*				
	3.1. Motivational interviewing		Less or no alcohol use, awareness	IV, X	+
	3.2. Personalized content and conversation			IV, X	+
	4. *The relationship between the client and peers*				
	4.1. Contact with peers	Recognition, connection, and support		IV, V, X	+
	4.2. Closed group and maximum group size	a. Create an emotionally safe space<br>b. More attention per client		I, IV	+
	4.3. Engagement, understanding, and support towards peers	Fellowship, connection, and safety	Less or no alcohol use	IV	+
	4.4. Addressing peers	Gain new insights	Less or no alcohol use	II, III, IV, V	+
	4.5. Sharing experiences and tips with peers	a. Being hopeful in own process		IV, V, X	

For peer review only - http://bmjopen.bmj.com/site/about/guidelines.xhtml

		b. Feeling recognized, relativizing own situation			+
	5. *The relationship between the client and practitioner*				
	5.1. Open attitude, not being judged, and accessible contact with practitioner			V	+
	6. *Other activities*				
	6.1. Having lunch after every session	Making connection and pleasant atmosphere		IV, V	+
	7. *Favorable setting*				
	7.1. Pleasant and relaxed atmosphere			I, IV	+
	7.2. Location is nearby home and/or familiar	Low threshold participation		I, IV	+
E. No practitioner—not in-person—individual	1. *Paying attention to drinking behavior*				
	1.1. Tracking alcohol use	Insight into use and tracking progress	Less or no alcohol use	III, X	+
	1.2. Abruptly quitting alcohol use	Positive learning experience		II, III	+
	1.3. Pointing out the risks and consequences of drinking behavior			III	+
	2. *Using tools*				
	2.1. *Web- and telephone-based interventions				~
	2.2. Online self-help tool	Accessible, low threshold, and controlled participation	Awareness, less or no alcohol use, going to treatment	III, X	+
	2.3. Via telephone			X	+
	2.4. Regular newsletter	Being actively engaged		III	+
	3. *The relationship between the client and peers*				
	3.1. Joining with others, contact with peers	Fellowship, recognition, and support		III	+
	4. *Favorable setting*				
	4.1. Participation independent of location	Accessible and low threshold		III	+
	5. *Additional help*				
	5.1. Personalized content and help additional to self-help			III	+
F. No practitioner – not in-person – group component	1. *Paying attention to drinking behavior*				
	1.1. *Intervention to abstinent people				~
	1.2. Focus on abstinence and paying attention to withdrawal			X	+
	2. *Self-help groups*				
	2.1. AA and self-help group			X	+
	3. *The relationship between the client and peers*				
	3.1.Contact with peers			IV, X	+
	3.2. Sharing experiences and tips with peers			IV, X	+

* Elements of initial program theory.
** Target group of the intervention. I = People with all types of alcohol use patterns; II = people with early-stage problematic alcohol use; III = people with all types of alcohol use patterns, except for (heavy) problematic alcohol use or alcohol addiction; IV = people with problematic alcohol use and alcohol addiction; V = people who are in recovery from addiction. X = No target group mentioned because these elements were derived from CEMO configuration that we presented as statements during the interviews.
***=✓ = Initial theory confirmed, ~ = initial theory refined, + = new theory added − = initial theory not confirmed.

Table 4. Summary of the program theory

Context (C)	Element of intervention (E)	Mechanism (M)*	Outcome (O)
A. Practitioner – in person – individual	Paying attention to drinking behavior	Changing perceptions of own use (1.1., 1.2.a., 1.2.b., 1.3.) and perceptions of consequences (1.4.)	Less or no alcohol use, awareness
	Paying attention to lifestyle		
	Communication approaches		
	The relationship between the client and practitioner	Improving communication (4.1.) and collaboration with practitioner (4.2.)	
	Favorable setting	Low threshold participation (5.1.)	
B. Practitioner – not in person – individual	Communication approaches	Low threshold participation (1.3.b.), tailoring (1.1., 1.2., 1.5.a.), improving communication with practitioner (1.3.a., 1.3.c.), engagement (1.5.b), and emotional support (1.5.c)	Less or no alcohol use, awareness, changing perspective on own alcohol use
	The relationship between the client and practitioner	Engagement and improving communication with practitioner (2.1.)	
	Providing additional help	Tailoring (3.1.)	
C . Practitioner—in-person—relatives	Teaching the partner to deal with drinking behavior	More understanding and support from the relative for the client (1.1.)	Less or no alcohol use
D. Practitioner—in-	Paying attention to drinking behavior		Less or no alcohol

For peer review only - http://bmjopen.bmj.com/site/about/guidelines.xhtml

person—group component	Paying attention to lifestyle<br>Communication approaches<br>The relationship between the client and peers<br><br>The relationship between the client and practitioner<br>Other activities<br>Favorable setting	Motivation (2.2.)<br><br>Social and emotional support (4.1., 4.3., 4.5.b.), pleasant atmosphere (4.2.a.), tailoring (4.2.b.), and changing perceptions of own use (4.4., 4.5.a., 4.5.b.)<br><br>Social contact and pleasant atmosphere (6.1.)<br>Low threshold participation (7.2.)	use, awareness
E. No practitioner— not in-person— individual	Paying attention to drinking behavior<br>Using tools<br>The relationship between the client and peers<br>Favorable setting<br>Additional help	Changes perceptions on own use (1.1., 1.2.)<br>Low threshold (2.2.) and engagement (2.4.)<br>Social and emotional support (3.1.)<br><br>Low-threshold participation (4.1.)	Less or no alcohol use
F. No practitioner – not in-person – group component	Paying attention to drinking behavior<br>Self-help groups<br>The relationship between the client and peers		

*= The mechanisms that are presented are the summarized mechanisms from Table 3, along with the numbering of the original mechanisms as indicated in Table 3.

For peer review only

DISCUSSION

Key Findings

Overall, five elements that were mentioned by more than one respondent were found in at least three contexts and can be labelled as general working elements: 1) *pointing out the risks and consequences of drinking behavior*; 2) *paying attention to abstinence*; 3) *promoting contact with peers*; 4) *providing personalized content;* and 5) *providing support*. We also found context-specific working elements, namely *providing personalized conversations* and *motivational interviewing,* specifically in interventions with practitioner involvement; *ensuring safety, trust, and a sense of connection* and *a location nearby home or a location that people are familiar with* in interventions in person; and *sharing experiences and tips* in interventions in group settings. Moreover, two mechanisms that were mentioned by more than one respondent were found in at least three contexts: *awareness* and *accessible and low threshold participation*. Our findings are generally in line with the IPT. However, we found many additional working elements, mechanisms, and outcomes that were posited by several professionals, resulting in a refined program theory.

Interpretation of Findings

The first addition to the IPT is that older adults need social contact and support. *Promoting contact with peers* can help reduce loneliness or social isolation, and *providing support* by a practitioner, peers or relatives can help deal with negative emotions and problematic alcohol use. Compared to the general adult population, older adults are more often facing retirement, illness, loss of relatives, loneliness, or hopelessness, which may facilitate negative health consequences of alcohol use (27, 28). It

For peer review only - http://bmjopen.bmj.com/site/about/guidelines.xhtml

is important to facilitate the process of helping older adults to develop contacts—for example in leisure settings *in a location that is nearby home and/or familiar*— to make participation low threshold.

The second addition to our IPT is that not only personalized feedback, but also *providing personalized content and conversations* is helpful for older adults, which is in line with findings in previous research showing that a personal approach with personalized feedback (23-25) and personalized reports (23, 24) is effective for older adults. Understanding the client's motives for alcohol use can help providers to be more specific and effective in providing personalized and tailored interventions. A tailored approach with consideration of the client's needs, with aging-related challenges taken into account, appears to be very effective (29).

The third addition to our IPT is that both *the relationship between the client and the practitioner* and *the relationship between the client and peers* are important in interventions. Previous research has shown that therapeutic alliance in the relationship between the client and the practitioner is associated with a reduced likelihood of dropout (30). Furthermore, older adults with substance use problems prefer interventions that are led by warm, honest, caring, and relatively non-confrontational providers (31). These findings are in line with our working element *ensuring safety, trust, and a sense of connection* and the importance of *the relationship between client and provider*.

In contrast, previous research has shown mixed findings on the relationship between the client and peers in substance use disorder populations: some research found a significant association between group cohesion and intervention outcomes, and group processes and outcomes (32-37), while other research found no significant association between group cohesion and outcomes (38, 39). Even though there seems to be no consensus in the literature on the association between the relationship between client and peers and intervention outcomes for the general adult population, we argue that there could be a strong association for older adults. Age-specific treatments may work better than mixed-age treatments with older adults (40). A group composition that matches the preferences of older adults could be a pre-requisite to *ensure safety, trust, and a sense of connection*, contributing to positive outcomes.

Finally, we found two important mechanisms that contribute to positive intervention outcomes. Firstly, *awareness* is an important mechanism in preventing or reducing problematic alcohol use. A previous study by Veerbeek and colleagues (2017) reported that older adults have limited awareness of

For peer review only - http://bmjopen.bmj.com/site/about/guidelines.xhtml

the negative health effects of alcohol, and that the majority of older adults in their study did not consider alcohol to be addictive or unhealthy. In addition, only a small proportion of older adults know the current standard for lower risk alcohol consumption (41), and people with problematic alcohol use often do not recognize their own drinking pattern as problematic (42, 43). This lack of knowledge emphasizes the importance among older adults of the working element *pointing out the risks and consequences of drinking behavior*. Emphasizing these risks can be experienced as threatening and therefore it is important to enhance older adults' self-efficacy in the intervention first (44). Furthermore, knowledge is a fundamental first step, but information provision alone is not sufficient for behavior change (45). Secondly, *accessible and low threshold participation* form an important mechanism in interventions for older adults. A major barrier for older adults seeking help is that they do not want to be labelled an 'alcoholic' or 'addict' (41), which can make participation in interventions that are provided in addiction care centers high threshold. Providing interventions in a location that people do not directly associate with alcoholism—e.g. a community center—can make participation low threshold. Another barrier for older adults can be difficulties with transportation (31). Travel allowances and accessible interventions can help older adults overcome these barriers.

Limitations and Strengths

This study has some limitations. In the Netherlands, people with lower socio-economic status and ethnic minorities are often not reached by lifestyle intervention efforts and show low participation rates (46, 47). It is possible that the interventions included in this study are less likely to reach these groups, which could mean that the professionals have limited experiences with these subgroups and that the overview of working elements and mechanisms is not fully adequate for these subgroups. It is therefore important to provide interventions with *personalized content and conversations* for specific subgroups, because this can provide a tailored approach. For example, older adults with a language deficiency could benefit from more specific elements in communication with the professional. Also, cultural differences may ask for different approaches. A study showed that religious older Moroccan-Dutch and Turkish-Dutch women can have other motivators (e.g. religious or cultural) to abstain from alcohol use than older native Dutch women (48). Further research should focus on what works for specific subgroups of older adults and to what extent these older adults participate in interventions to reduce problematic alcohol use. The second limitation concerns biases when the CEMO statements were presented at the end of the interview. It is possible that acquiescence bias occurred here, meaning that respondents perhaps agreed more easily to these statements because they responded in ways they thought the

For peer review only - http://bmjopen.bmj.com/site/about/guidelines.xhtml

researcher wanted them to respond, or because they wanted to finish the interview. Respondents were invited to explain their opinions on these statements, but they often responded only by saying that they agreed with these statements without explanation. Habituation bias could also have occurred when the participants provided the same answers in response to similarly-worded CEMO statements. These two biases may influence our confirmed IPT. Finally, some CEMO configurations were only confirmed when the statements were presented. Some working elements—e.g. empathic behavior of the practitioner—might be so obvious that they were not initially mentioned. We think that there could be more such elements that are missing in our overview because of these factors.

Strengths of this study are that we were able to include a variety of professionals across all eleven interventions, and the high response rate. Another strength is the realist evaluation approach used, as this provides explanations from the perspective of professionals of what works to reduce problematic alcohol use among older adults. This approach provides in-depth insights into interventions because it can explain why interventions are successful or not.

Practical and Research Implications

Based on our findings, we have a number of recommendations for practice and future research. Professionals and developers of interventions might focus on three aspects. Firstly, practitioners should pay attention to the elements related to the role of the practitioner in in-person interventions, more specifically in existing brief interventions which are already often used by practitioners. Secondly, developers of interventions need to pay particular attention to accessible or low threshold participation—for example by providing interventions in a *favorable setting*. Thirdly, practitioners and developers together should focus more on the element *promoting contact with peers*. For example, practitioners working in group interventions can invite clients to *share experiences and tips* with peers, and developers of online self-help tools can refer people to online forums where people can *share experiences and tips* with peers.

Further research should focus on the perspectives of clients who participate in interventions and their relatives to refine the program theory. Also, further research should focus on interventions in the context with a practitioner, not in-person, and individual and in the context with no practitioner, not in-person, and in groups, because elements in these contexts are limited in this study.

CONCLUSION

For peer review only - http://bmjopen.bmj.com/site/about/guidelines.xhtml

In addition to the previous formulated IPT, our findings emphasize the need for social contact and support, personalized content, and strong relationships (between client and practitioner, and client and peers) in interventions for older adults. Whereas the working element 'social contact' was not found as a working element in previous literature, probably due to the limited number of studies performed, our study found this to be an additional important working element, and providers of alcohol interventions should be aware of and make use of this element when treating older adults. Future research should study the perspective of older adults themselves and their relatives on working elements and mechanisms in interventions.

Contributors

FvdB, ADR, RC, RB and DvdM developed the research questions and study methodology. FvdB and ADR developed an interview guide an RC, RB and DvdM provided inputs to the interview guide. Data were collected by FvdB. Data were coded by FvdB and co-coded by RK, and checked by ADR. ADR, RC, RB, DvdM, IvdG, and SS then provided feedback on the code-document. FvdB produced the draft manuscript under daily supervision of ADR. ADR, RC, RB, DvdM, IvdG, and SS have reviewes the manuscript critically for important content.

Funding

This research was funded by The Netherlands Organisation for Health Research and Development (ZonMw number: 555002034).

Competing Interests

The authors declare no conflict of interest.

Patient and public involvement

Patients and/or the public were not involved in the design, conduct, reporting, or dissemination plans of this research.

Ethics Approval

The study was approved by the Ethics Review Board at Tilburg University (RP508). Participants gave informed consent to participate in the study before taking part.

Data Availability Statement

No additional data are available.

For peer review only - http://bmjopen.bmj.com/site/about/guidelines.xhtml

REFERENCES

1. Breslow RA, Castle IJP, Chen CM, Graubard BI. Trends in Alcohol Consumption Among Older Americans: National Health Interview Surveys, 1997 to 2014. Alcoholism: Clinical and Experimental Research. 2017;41(5):976-86.
2. Han BH, Moore AA, Sherman S, Keyes KM, Palamar JJ. Demographic trends of binge alcohol use and alcohol use disorders among older adults in the United States, 2005–2014. Drug and Alcohol Dependence. 2017;170:198-207.
3. Kim JW, Lee DY, Lee BC, Jung MH, Kim H, Choi YS, et al. Alcohol and Cognition in the Elderly: A Review. Psychiatry Investigation. 2012;9(1):8.
4. Roche AM, Kostadinov V. Baby boomers and booze: we should be worried about how older Australians are drinking. Medical Journal of Australia. 2019;210(1):38-9.
5. Wolf I-K, Du Y, Knopf H. Changes in prevalence of psychotropic drug use and alcohol consumption among the elderly in Germany: results of two National Health Interview and Examination Surveys 1997-99 and 2008-11. BMC Psychiatry. 2017;17(1).
6. Mauro PM, Askari MS, Han BH. Gender differences in any alcohol screening and discussions with providers among older adults in the United States, 2015 to 2019. Alcoholism: Clinical and Experimental Research. 2021;45(9):1812-20.
7. Grant BF, Chou SP, Saha TD, Pickering RP, Kerridge BT, Ruan WJ, et al. Prevalence of 12-Month Alcohol Use, High-Risk Drinking, and <i>DSM-IV</i> Alcohol Use Disorder in the United States, 2001-2002 to 2012-2013. JAMA Psychiatry. 2017;74(9):911.
8. Knott CS, Scholes S, Shelton NJ. Could more than three million older people in England be at risk of alcohol-related harm? A cross-sectional analysis of proposed age-specific drinking limits. Age Ageing. 2013;42(5):598.
9. Meier P, Seitz HK. Age, alcohol metabolism and liver disease. Current Opinion in Clinical Nutrition and Metabolic Care. 2008;11(1):21.
10. Cederbaum AI. Alcohol Metabolism. Clinics in Liver Disease. 2012;16(4):667-85.
11. Moore AA, Whiteman EJ, Ward KT. Risks of combined alcohol/medication use in older adults. The American Journal of Geriatric Pharmacotherapy. 2007;5(1):64-74.
12. Kuerbis A, Sacco P, Blazer DG, Moore AA. Substance abuse among older adults. Clinics in geriatric medicine. 2014;30(3):629-54.
13. Schonfeld L, Dupree LW. Treatment Approaches for Older Problem Drinkers. International Journal of the Addictions. 1995;30(13-14):1819.
14. Dupree LW, Broskowski H, Schonfeld L. The Gerontology Alcohol Project: A Behavioral Treatment Program for Elderly Alcohol Abusers. The Gerontologist. 1984;24(5):510.
15. Rice C, Longabaugh R, Beattie M, Noel N. Age group differences in response to treatment for problematic alcohol use. Addiction. 1993;88(10):1369-75.
16. Schonfeld L, Hazlett RW, Hedgecock DK, Duchene DM, Burns LV, Gum AM. Screening, brief intervention, and referral to treatment for older adults with substance misuse. American journal of public health. 2015;105(1):205-11.
17. Seddon JL, Wadd S, Wells E, Elliott L, Madoc-Jones I, Breslin J. Drink wise, age well; reducing alcohol related harm among people over 50: a study protocol. BMC Public Health. 2019;19(1).
18. Schultz SK, Arndt S, Liesveld J. Locations of facilities with special programs for older substance abuse clients in the US. International Journal of Geriatric Psychiatry. 2003;18(9):839-43.
19. Armstrong-Moore R, Haighton C, Davinson N, Ling J. Interventions to reduce the negative effects of alcohol consumption in older adults: a systematic review. BMC Public Health. 2018;18:13.

For peer review only

For peer review only - http://bmjopen.bmj.com/site/about/guidelines.xhtml

20. Megherbi-Moulay O, Igier V, Julian B, Franchitto N, Sordes F. Alcohol Use in Older Adults: A Systematic Review of Biopsychosocial Factors, Screening Tools, and Treatment Options. International Journal of Mental Health and Addiction. 2022.

21. Pawson R, Tilley N, Tilley N. Realistic Evaluation: SAGE Publications; 1997.

22. Boumans J, van de Mheen D, Crutzen R, Dupont H, Bovens R, Rozema A. Understanding How and Why Alcohol Interventions Prevent and Reduce Problematic Alcohol Consumption among Older Adults: A Systematic Review. Int J Environ Res Public Health. 2022;19(6):3188.

23. Ettner SL, Xu H, Duru OK, Ang A, Tseng C-H, Tallen L, et al. The Effect of an Educational Intervention on Alcohol Consumption, At-Risk Drinking, and Health Care Utilization in Older Adults: The Project SHARE Study. Journal of Studies on Alcohol and Drugs. 2014;75(3):447-57.

24. Fink A, Elliott MN, Tsai M, Beck JC. An Evaluation of an Intervention to Assist Primary Care Physicians in Screening and Educating Older Patients Who Use Alcohol. Journal of the American Geriatrics Society. 2005;53(11):1937-43.

25. Kuerbis AN, Yuan SE, Borok J, Lefevre PM, Kim GS, Lum D, et al. Testing the Initial Efficacy of a Mailed Screening and Brief Feedback Intervention to Reduce At-Risk Drinking in Middle-Aged and Older Adults: The Comorbidity Alcohol Risk Evaluation Study. Journal of the American Geriatrics Society. 2015;63(2):321-6.

26. Tong A, Sainsbury P, Craig J. Consolidated criteria for reporting qualitative research (COREQ): a 32-item checklist for interviews and focus groups. International Journal for Quality in Health Care. 2007;19(6):349.

27. Kuerbis A, Sacco P. The impact of retirement on the drinking patterns of older adults: a review. Addictive Behaviors. 2012;37(5):587.

28. Barry KL, Blow FC. Drinking over the lifespan: Focus on older adults. Alcohol Research: Current Reviews. 2016;38(1):115.

29. Atiq Rafay R. Common themes and issues in geriatric psychotherapy. Psychiatry (Edgmont (Pa : Township)). 2006;3(6):53-6.

30. Meier PS, Donmall MC, McElduff P, Barrowclough C, Heller RF. The role of the early therapeutic alliance in predicting drug treatment dropout. Drug and Alcohol Dependence. 2006;83(1):57-64.

31. Holland JM, Rozalski V, Beckman L, Rakhkovskaya LM, Klingspon KL, Donohue B, et al. Treatment Preferences of Older Adults with Substance Use Problems. Clinical Gerontologist. 2016;39(1):15-24.

32. Valeri L. Group therapy for women with substance use disorders: In-session affiliation predicts women's substance use treatment outcomes. Journal of Substance Abuse Treatment. 2018;94:60.

33. Kelly PJ, Deane FP, Baker AL. Group Cohesion and Between Session Homework Activities Predict Self-Reported Cognitive–Behavioral Skill Use Amongst Participants of SMART Recovery Groups. Journal of Substance Abuse Treatment. 2015;51:53-8.

34. Cavicchioli M. Group relationships during a dialectical behavior therapy skills training program for the treatment of alcohol and concurrent substance use disorders: Evidence and theoretical considerations. Group Dynamics: Theory, Research, and Practice. 2021;25(2):152.

35. Sotskova A. Understanding the role of group cohesion and group alliance in a secular peer support group for recovery from substance misuse. Journal of Groups in Addiction and Recovery. 2016;11(2):137.

36. Rice SL, Tonigan JS. Impressions of Alcoholics Anonymous (AA) Group Cohesion: A Case for a Nonspecific Factor Predicting Later AA Attendance. Alcoholism Treatment Quarterly. 2012;30(1):40-51.

37. Pooler DK, Qualls N, Rogers R, Johnston D. An Exploration of Cohesion and Recovery Outcomes in Addiction Treatment Groups. Social Work with Groups. 2014;37(4):314-30.

38. Cruz M, Osilla KC, Paddock SM. Group Cohesion and Climate in Cognitive Behavioral Therapy for Individuals with a First-Time DUI. Alcoholism Treatment Quarterly. 2020;38(1):68-86.

For peer review only - http://bmjopen.bmj.com/site/about/guidelines.xhtml

39. Gillaspy JA, Wright AR, Campbell C, Stokes S, Adinoff B. Group Alliance and Cohesion as Predictors of Drug and Alcohol Abuse Treatment Outcomes. Psychotherapy Research. 2002;12(2):213-29.
40. Kuerbis A, Sacco P. A Review of Existing Treatments for Substance abuse among the Elderly and Recommendations for Future Directions. Substance Abuse: Research and Treatment. 2013;7:SART.S7865.
41. Veerbeek M, Heijkants C, Willemse B. Alcoholgebruik onder 55-plussers. Utrecht: Trimbos Instituut. 2017.
42. Smith JJ, Spanakis P, Gribble R, Stevelink SAM, Rona RJ, Fear NT, et al. Prevalence of at-risk drinking recognition: A systematic review and meta-analysis. Drug and Alcohol Dependence. 2022;235:109449.
43. Morris J, Albery IP, Moss A, Heather N. Promoting problem recognition amongst harmful drinkers: A conceptual model for problem framing factors. The Handbook of Alcohol Use. 2021:221.
44. Peters G-JY, Ruiter RAC, Kok G. Threatening communication: a critical re-analysis and a revised meta-analytic test of fear appeal theory. Health Psychology Review. 2013;7(sup1):S8-S31.
45. Arlinghaus KR, Johnston CA. Advocating for Behavior Change With Education. American Journal of Lifestyle Medicine. 2018;12(2):113-6.
46. El Fakiri F, Hoes AW, Uitewaal PJM, Frenken RAA, Bruijnzeels MA. Process evaluation of an intensified preventive intervention to reduce cardiovascular risk in general practices in deprived neighbourhoods. European Journal of Cardiovascular Nursing. 2008;7(4):296.
47. Magnée T, Burdorf A, Brug J, Kremers SPM, Oenema A, Van Assema P, et al. Equity-Specific Effects of 26 Dutch Obesity-Related Lifestyle Interventions. American Journal of Preventive Medicine. 2013;44(6):e61-e70.
48. Sialino LD, Wijnhoven HAH, Van Oostrom SH, Picavet HSJ, Verschuren WMM, Visser M, et al. Perspectives of older women in the Netherlands: identifying motivators and barriers for healthy lifestyles and determinants of healthy aging. BMC Public Health. 2023;23(1).

For peer review only

For peer review only - http://bmjopen.bmj.com/site/about/guidelines.xhtml

Supplementary Table 1. Comprehensive program theory

Context (C)	Element of intervention (E)	Mechanism (M)	Outcome (O)
**A. Practitioner – in-person – individual**	(No element, context only)	No misery others (II)<br>Practitioner functions as a big stick (II)<br>Dare to discuss things face to face better than online (IV)	
	1. Paying attention to drinking behavior		
	1.1 Paying attention to drinking behavior		
	1.1.1. Paying attention to drinking behavior	Thinking and awareness	Less or no alcohol use (X)
	1.1.2. Conversation with practitioner about alcohol use (X)		
	1.2 Tracking alcohol use and reflecting on use		
	1.2.1. Reflecting on alcohol use (II, X)	Insight to use (II)<br>Confrontation and reflection (II)	
	1.2.2. Tracking alcohol use (II)	Insight to use (II, III, IV)<br>Confrontation (II)	
	1.2.3. Paying attention to the cause of alcohol use (X)	-<br>Thinking about use/lifestyle (II)	Less or no alcohol use (X)<br>Awareness and insight alcohol use (II)
	1.3. Paying attention to abstinence, coping planning, and practicing with abstinence		
	1.3.1. Coping planning abstinence (I, IV, X)		
	1.3.2. Practicing with abstinence	-	Awareness alcohol use (IV)
	1.3.3. Raising awareness of responsible use/abstinence (I)	Awareness alcohol use (I)	
	1.4. Pointing out the risks and consequences of drinking behavior		
	1.4.1. Information about risks and consequences (I, II, IV, X)	Thinking about and recognize indirect consequences in own vulnerability (I, II)	Awareness of effects of alcohol use, less or no use (I, II)
		Shocked by consequences<br>Insight to use (IV)	Awareness of health effects of alcohol use (I)
		Increase risks perception for consequences Motivation to reduce	Less alcohol use (IV)
	2.Paying attention to lifestyle		
	2.1. Paying attention to meaning of life and teaching lifestyle changes		
	2.1.1. Attention and tips of meaning of life	Meaning in life	Less or no alcohol use (II)
	2.1.2. Teaching lifestyle changes (II)		
	2.1.3. Attention to different aspects of life (II)		
	3. Communication approaches		
	3.1. Motivational interviewing		
	3.1.1 Motivational interviewing (X, II)	Connect with client (X)<br>-	Awareness (X)
	3.2. Personalized content and conversation		
	3.2.1. Personalized content and conversation (I, II)		

4. The relationship between the client and practitioner			
4.1. Safety, trust, connection, and not being judged in contact with practitioner			
4.1.1. Client feels comfortable with practitioner (II)			
4.1.2. Low-threshold contact with practitioner (X)	-		Less or no alcohol use
4.1.3. Contact with practitioner feels safe	Opening conversation to discuss drinking behavior (X)		
4.1.4. Client feels accepted	Opening conversation to discuss drinking behavior (X)		
4.1.5. Client feels connection with practitioner	Opening conversation to discuss drinking behavior (X)		
4.1.6. Practitioner has an open attitude and does not judge	Client feels comfortable<br>-		Opening up at follow-up conversation (II)<br>Moderate drinking/awareness (II)
4.1.7. Good therapeutic relationship (X)			
4.1.8. Practitioner is friendly	Client feels comfortable		Openness at follow-up conversation (II)
4.2. Empathic behavior of practitioner			
4.2.1. Practitioner is empathic (I, X)	Client feels understood about alcohol-problems (X)	Client and practitioner collaborate in the identification of additional help, and the relationship between the client and the therapist improves	Less or no alcohol use (X)
	Client and practitioner collaborate in the identification of additional help, and the relationship between the client and the therapist improves (X)		
	Good relationship of trust (X)		
	Client becomes more honest and willing to discuss things (X)		
4.3. Critical, controlling, and confronting behavior of practitioner			
4.3.1. Practitioner has active role/guidance (II)	Engagement (II)		
4.3.2 Practitioner makes client reflect critically on client's actions (IV)	-		Less or no alcohol use (X)
4.3.3 Practitioner is critical/strict (II)			
4.3.4 Practitioner sets boundaries (IV)			
4.3.5 Practitioner confronts (II)			
5. Favorable setting			
5.1. Location is nearby home and/or familiar			
5.1.1. Location is nearby home and/or familiar	Low-threshold participation (I, IV)<br>Less effort to go the location		 Increase chances of participation (IV)
5.2. * Pleasant atmosphere			
5.2.1. Meeting with friendly and non-committal atmosphere	Clients can decide for themselves what they will and will not discuss (I)		
*6. * Other*			
6.1. Additional contact			

	6.1.1. Introductory interview	Alignment between practitioner and client		Client wants follow-up interview (II)
	6.1.2. Multiple sessions	More attention for (different) themes and needs (II)		
	6.1.3. Conversation with general practice-based nurse specialist/GP (II)			
	6.2. Other characteristics/input practitioner			
	6.2.1. Practitioner has much knowledge	Thinking about alcohol use		Motivation less alcohol use(X)
	6.2.2. Practitioner shares own experience	Equal contact	Connection between practitioner and client	Client is open for what practitioner has to offer (IV)
**B. Practitioner – not in-person – individual**	(No element, context only)	Practitioner functions as a big stick (X)		
	1. Communication approaches			
	1.1. Contact always possible			
	1.1.1.Always the possibility of contact (I)	Accessible help in difficult situations		Less or no alcohol use, think differently about alcohol use, being open about alcohol use (I, III)
	1.2. Conversation over the phone			
	1.2.1. Telephone conversation (I)	Accessible and flexible (distance, time and anonymity) (I, X)<br>Responding to emotions (I)		
	1.3. Anonymous			
	1.3.1. Anonymous and telephone conversation (I)	Distance between practitioner and client	Client is more likely to accept advice (I)<br>Client speaks and opens up easier (I)	
		Low-threshold (I)<br>Less attention, shame (I)		
		-		Think differently about alcohol (I)
		-		Being open about alcohol use (I)
	1.3.2. No relationship between client and practitioner	Honest instead of socially desirable contact		Think differently about alcohol use (I)<br>Being open about alcohol use (I)
	1.4. Motivational interviewing			
	1.4.1. Motivational interviewing	Engagement		Awareness alcohol use (I, X)
		Open communication and client accept advice from practitioner (I)		
	1.5. Personalized content and conversation			
	1.5.1. Personalized content and conversation (I)	Tailoring to needs and level (I)		Increase knowledge and motivation (I)
		Non-commitment disappears		Less or no alcohol use (III)
		-		Knowledge about where to go when seeking help, learning to cope with alcohol use, increasing motivation for less or no alcohol use (I)
		Pick up emotions (I)	Establish a relationship	Communicate message better to client (I)<br>Make the client think critically (I)
		Possibility of attention other problems (III)		Insight into the cause of the problem (III)

		Feeling of support		Less or no alcohol use (III, X)
	1.6. Personal contact and feedback			
	1.6.1. Personal contact and feedback (X)	Non-commitment disappears (X)		Less or no alcohol use (X)
	1.7. * Other			
	1.7.1. Five-phase model	Open communication and clients accept advice from practitioner (I)		Awareness alcohol use (I)
	1.7.2. Structure, clear expectations and agreements for client (III)	A big stick		Awareness alcohol use (III)
	1.7.3. Regular contact	A big stick	Motivation	Awareness alcohol use, less or no alcohol use (III)
	2. The relationship between the client and practitioner			
	2.1. Practitioner is empathic, supportive, and listens			
	2.1.1 Gentle approach with compliments (I)			
	2.1.2 Practitioner does not judge about alcohol behavior, supports and listen (I)			
	2.1.3 Practitioner support and listens (I)			
	2.1..4 Practitioner is empathic	Engagement	Clients accept information from practitioner<br>Open communication	Client will seek help from addiction services (I)
	2.2. * Practitioner takes away the shame with positive approach	Reduce craving for alcohol (I)		
	3. Providing additional help			
	3.1. Personal treatment with practitioner additional to self-help			
	3.1.1 Personal treatment with practitioner additional to selfhelp (III, X)	Working independent (III)<br>Keep more attention<br>Provide extra tailoring (III)<br>Possibility of involving relatives (III)<br>Recognition (III)		More likely to persist (X)
	*4. *Paying attention to drinking behavior*			
	4.1. Information about risks and possible help			
	4.1.1. Information about health risks	-		Increase knowledge about alcohol (I)
	4.1.2. Information about alcohol interventions (I)			
**C. Practitioner—in-person—relatives**	*1. Teaching the partner to deal with drinking behavior*			
	1.1 Teaching the partner to deal with drinking behavior			
	1.1.1. Teaching the partner about/dealing with drinking behavior(I, IV, X)	Partner gets more trust in client<br>More understanding and support from the relative for the client (X)		Growing together and taking steps (IV)
	2. Support of relatives			
	2.1 Relative provides support			
	2.1.1.Relatives are deployed to provide support (X)	-		Less or no alcohol use
	*3. * Other*			

	3.1. Relative controls			
	3.1.1. Relative gives or controls information (II)			
	3.2. Partner shares experience and insights with other partners			
	3.2.1 Partner shares experience and insights with other partners	Partner is relaxed and is less controlling		Easier for client to achieve goals (IV)
**D. Practitioner – in-person – group component**	(No element, context only)	Togetherness, connection, recognition (I)	Sharing 'stick behind the door' experience	Know how to improve quality of life during abstinence, abstinence (IV)
			-	Less or no alcohol use (IV)
		Recognition		Achieving goals (IV)
		Location gets people outside where addiction takes place (IV)		
		Safety (V)		
		Free to share things by others talking (I)		
	1. Paying attention to drinking behavior			
	1.1. Pointing out the risks and consequences of drinking behavior			
	1.1.1. Information about risk and consequences (IV, V)	Insight in addiction (IV)		
	1.2. Paying attention to abstinence and practicing with abstinence			
	1.2.1. Attention and tools for (sustained) abstinence	-		Less or no alcohol use (IV)
		Less alcohol use	Mood improves	People become more active and anxiety symptoms will reduce (IV)
	1.2.2. Practicing with new or tense situations	Learning to tolerate discomfort in life (IV)		
		Gain experience (IV)		
	1.3. *Discuss statements on alcohol use in groups			
	1.3.1. Discuss statements in groups	Thinking about alcohol use (I)		
		Openness about alcohol use (I)		
	2. Paying attention to lifestyle			
	2.1. ** Motivating to change lifestyle			
	2.2. Attention and tips regarding meaning of life, lifestyle, and problems when aging			
	2.2.1 Attention for meaning of life and life stage themes (IV, X)	Motivation to go to the next session (IV)		
		Better coping with cravings for alcohol (IV)		
		Motivation for change		Abstinence, know how to improve quality of life during abstinence (IV)
	2.1.2. Attention for lifestyle or motivate lifestyle change (X)	-		Less alcohol use (X)
	2.3. **Motivating to change lifestyle delivered in a workplace setting			

2.4. In a workplace setting and paying attention to prevention and lifestyle			
2.4.1. At work (X)	Increase awareness (X)		
2.4.2. At work with attention for prevention (X)			
2.4.3. At work with attention for lifestyle (X)			
2.4.4. * At work, openness about alcohol use	Negative consequences (X)		
	Support environment (X)		
	-		Less alcohol use (X)
2.4.5. * At work, people join national campaign (X)			
3. Communication approaches			
3.1. Motivational interviewing			
3.1.1. Motivational interviewing	-		Less or no alcohol use, awareness (X, IV)
3.2. Personalized content and conversation			
3.2.1. Personalized content and conversation (IV, X)			
3.2.2. * Customized CBT treatment (IV)			
3.3. * Other approaches			
3.3.1. Change way of thinking from negative to positive (IV)	Rethinking and self-pity reduces	Getting stronger to prevent relapse	Less or no alcohol use (IV)
3.3.2. Overcoming shame			Less or no alcohol use (III)
3.3.3. Mindfulness and running therapy (V)	Feeling signals in body and recognize (V)		
3.3.4. CGT focusing on substance use (IV)	-		Less or no alcohol use
3.3.5. Structure in days	Having rhythm and purpose to go somewhere (V)		
3.3.6. Variance of working methods (IV)			
4. The relationship between the client and peers			
4.1.Contact with peers			
4.1.1. Contact with peers (IV, IV, IV, X)	Being seen and feel supported (IV)		
	Connection (IV)		
4.1.2. Group composition like-minded peers (IV, IV)	Recognition and connection (IV,V, X)		
4.2. Closed group and maxim group size			
4.2.1.Maximized group size	Creating safety		Preventing drop out (IV)
	More attention per client (I, IV)		
4.2.2.Closed group	Improves atmosphere (IV)		
	Ongoing process (IV)		
	Safety	Daring to open up (IV)	
4.3. Engagement, understanding, and support towards peers			
4.3.1. Engagement and understanding each other (IV)	Connection (IV)		
	Safety	Talking about problems (IV)	Less or no alcohol use (IV)
	Togetherness, being able to ask for help from each other (IV)		

For peer review only

4.3.2. Support each other (V)			
4.3.3. People feeling heard in group	Less alcohol use	Mood improves	People become more active and anxiety symptoms will reduce (IV)
4.4. Addressing peers			
4.4.1. Addressing each other, reflect critically on your own actions (II, V)	Learning and getting insights		Less or no alcohol use (III, IV)
4.5. Sharing experiences and tips with peers			
4.5.1. Sharing experiences and tips (V, X)	Hopeful for own process (IV, V)<br>Openness about alcohol use (IV)<br>Recognition and acknowledgement (IV)<br>Putting own situation in perspective (IV)<br>Feeling the impact of addiction (IV)		
4.6. * Making agreements with peers			
4.6.1. Making agreements among themselves in group	A big stick (IV)		
5. The relationship between the client and practitioner			
5.1. Open attitude, not being judged and accessible contact with practitioner			
5.1.1. Practitioner is unbiased, open and not moralizing (V)			
5.1.2. Accessible contact practitioner	Experience support		Less relapse in alcohol use (V)
5.2. * Other			
5.2.1. Practitioner has the same age of clients (IV)	Recognition and click (IV)		
5.2.2. Presence two practitioners (IV)			
5.2.3. Interactive contact	Holding attention (I)		
5.2.3. Guest speakers focusing on different lifestyle themes (IV)	-		Abstinence, know how to improve quality of life during abstinence (IV)
6. Other activities			
6.1. Having lunch after every session			
6.1.1. After every session have a lunch together (IV, V)	Connection (IV)		Less or no alcohol use (IV)
	Atmosphere in the group (IV)		
	Unforced atmosphere		Discuss topics in a low-threshold way that are more difficult during a session (IV)
			Safe setting (IV)
	Relaxation and getting to know each other better (IV)		
	Insight of simplicity nutritious meal (IV)		
6.2. * Activities			
6.2.1. Social activities (V)	Daytime activities	Reduce loneliness (IV)	
6.2.2. Outdoor intervention in group walking with app	Stimulate to move or exercise		Better coping with cravings for alcohol (IV)

	7. Favorable setting			
	7.1. Pleasant and relaxed atmosphere			
	7.1.1. Meeting with friendly and non-committal atmosphere	Clients can decide for themselves what they will and will not discuss (I)		
	7.1.2. Besides serious atmosphere, also relaxed atmosphere with humor (IV)			
	7.2. Location is nearby home and/or familiar			
	7.2.1. Location is nearby home and/or familiar	Low-threshold participation (I, IV)		
		Less effort to get to location	Increase chance of participation (IV)	
	7.3. * Location is not admission clinic			
	7.3.1. Location is not admission clinic	People are not remembered by their admission (V)		
**E. No practitioner – not in-person - individual**	*1. Paying attention to drinking behavior*			
	1.1. Tracking alcohol use			
	1.1.1.Monitoring alcohol use	Keep track of progress		Less alcohol use (III)
	1.1.2. Tracking alcohol use in app/telephone	Insight into alcohol use(X)		Less or no alcohol use (X)
	1.1.3. * Information and tools relapse and dealing with difficult moments	Prevent relapse	Handle difficult moments better and consciously (III)	
	1.2. Abruptly quitting alcohol use			
	1.2.1. Abruptly quit alcohol use for a temporary period (III)	Learning experience that can be perceived as positive (II)	High self-efficacy and attitude change towards alcohol use (III)	High probability that people will use less or no alcohol use later (again) (III)
		Awareness behavioral change (III)		
	1.3. Pointing out the risks and consequences of drinking behavior			
	1.3.1. Paying attention to risks of alcohol use at age	Get people thinking (III)		
	1.4. * Receiving feedback			
	1.4.1. Receiving feedback	Motivation		Less alcohol use (III)
	1.4.2. Positive feedback	Motivation		Less alcohol use (III)
	2. Using tools			
	2.1. ** Web based interventions			
	2.2. Online self-help tool			
	2.2.1.App	No waiting list so quick participation (III)<br>Getting started with themes in a low-threshold way (X)		
	2.2.2. App that is simple and clear	Accessible (III)		
	2.2.3. Online self-help tool /program /module	Own direction implementation (III)<br>Low-threshold(III)		Less or no alcohol use (X)
		-		Awareness/ Less or no alcohol use/ Step towards treatment (IV)
	2.2.4. Short online module with playful approach (III)	Awareness		Small changes alcohol use (III)

	2.3. Via telephone		
	2.3.1. Via telephone	Saving time/cost (X)	
	2.4. Regular newsletter		
	2.4.1. Regular newsletter (III)	Being actively engaged Support (III)<br>Recognition support (III)<br>-	 <br>Being motived to use less or no alcohol use for longer periods of time (III)
	2.5. * Other		
	2.5.1. Webinar with peer chatbox	Personal contact what serves as social support (III)	
	3. The relationship between the client and peers		
	3.1. Joining with others, contact with peers		
	3.1.1. Additional contact with peers (III)		
	3.1.2. Joining with others (III)	Feeling of togetherness (III)<br>Feeling of recognition (III)<br>Social support (III)	Increase success of intervention (III)
	4. Favorable setting		
	4.1. Participation independent of location		
	4.1.1. Participation independent of location /participation possible from home (III)	Accessible and low-threshold (III)	
	5. Additional help		
	5.1. Personalized content and help additional to self-help		
	5.1.1. Additional personal coaching at self-help interventions (III)		
	5.1.2. Additional physical tools for client (III)	Feeling of physical participation at home (III)	
	5.1.3. Extra options for support (III)	Feeling of personal, voluntary and non-committal participation	Less or no alcohol use for one month (III)
	*6. * Other*		
	6.1. Attention other themes		
	6.1.1. Attention to activity spending	Get people thinking (III)	
	6.1.2. Attention for difficult aspects in life	Get people thinking (III)	
	6.1.3. Commitment with yourself by signing up (III)	Motivation	Increase success of intervention (III)
	6.1.4. Non commitment participation	Low-threshold (III)<br><br>Extra motivation (III)	Makes less or no alcohol use pleasant, step up to intensive help (III)
	6.1.5. Attention to positive experiences	Increase self-efficacy (III)	
	6.1.6. Setting goals	-	Less alcohol use (III)
**F. No practitioner – not in-person – group component**	(No element, context only)	Motivation and not being alone	Less or no alcohol use (X)
	1. Paying attention to drinking behavior		
	1.1. **Intervention to abstinent people		
	1.2. Focus on abstinence and paying attention to withdrawal		

1.2.1. Focus on abstinence, with attention to withdrawal (X)		
1.2.2. Focus on abstinence (X)		
2. Self-help groups		
2.1. AA and self-help group		
2.1.1. Aa/self-help group (X)	-	Less or no alcohol use (II)
3. The relationship between the client and peers		
3.1. Contact with peers		
3.1.1. Peer contact (IV, X)	Recognition (X)	
3.2. Sharing experiences and tips with peers		
3.2.1. Sharing experiences and tips with peers (III)	Hope and connection (X)<br>Persuade to participate (X)<br>Stimulate (X)	
3.3. * Other		
3.3.1. Composition of people with same level/serious issues (III)		
3.3.2. Making agreements and setting goals in group	-	Less or no alcohol use (X)
3.3.3. Helping each other (X)		
3.3.4. Online forum	Social support	Temporary stop of alcohol use (III)
*4. * Paying attention to other aspects*		
4.1. Broader focus than abstinence		
4.1.1. Broader focus than only abstinence	Thinking about what is important	Less or no alcohol use (X)

*= Program theories that were not included in Table 3. ** Initial program theories that were refined or not confirmed.
I = People with all types of alcohol use patterns; II = people with early stage problematic alcohol use; III = people with all types of alcohol use patterns, except for (heavy) problematic alcohol use or alcohol addiction; IV = people with problematic alcohol use and alcohol addiction; V = people who are in recovery from addiction. X = No target group mentioned because these elements were derived from CEMO configuration that we presented as statements during the interviews.

For peer review only

Supplementary Material 1

The COREQ Checklist

**Domain 1: Research team and reflexivity**		
Personal characteristics		
Interviewer/facilitator	Fieke van den Bulck	n/a
Credentials	MSc	Not reported in manuscript
Occupation	PhD	Not reported in manuscript
Gender	Woman	Not reported in manuscript
Experience and training	Conducted interviews as a part of her Ph.D.	Not reported in manuscript
Relationship with participants		
Relationship established	yes	Not reported in manuscript
Participant knowledge of the interviewer	Created relationship during preparation for the research study .	Not reported in manuscript
Interviewer characteristics		Not reported
**Domain 2: Study design**		
Theoretical framework		
Methodological orientation	Interviews with a realist evaluation approach	p. 5, 8
Participant selection		
Sampling	Convenience sampling	p. 6
Method of approach	Email or telephone	p. 4
Sample size	20 interviews with health care worker, prevention worker, coach, psychiatric mental health nurse, psychologist, peer support worker, volunteer, founder of intervention, advisor, coordinator, manager	p. 6, 7
Non-participation	5 did not participate	p. 6
Setting		
Setting of data collection	The interviews professionals were conducted via Microsoft Teams, Skype or telephone, between June and December 2022	p. 7
Presence of non-participants	No	Not reported
Description of sample		p. 6, 7
Data collection		
Interview guide		p. 7
Repeat interviews	No	Not reported in manuscript
Audio/visual recordings	Audio for Skype and telephone, visual recordings for Microsoft Teams interviews	Not reported in manuscript
Field notes	Yes	Not reported

For peer review only

Duration	The interviews ranged from 40.4 to 85.4 minutes	p. 7
Data saturation	Interviews were conducted until data saturation occured	p. 7
Transcripts returned	N/a	Not reported
**Domain 3: analysis and findings**		
Data analysis		
Number of data coders	2	p. 9
Description of the coding tree		p. 9
Derivation of themes	Themes were derived from data	p. 9
Software	Atlas.TI	p. 9
Participant checking	No	Not reported
Reporting		
Quotations presented	Yes, and each quotation is identified	p. 9-12
Data and findings consistent	Yes	
Clarity of major themes	Yes	p. 9-12, 14-17
Clarity of minor themes	Yes	p. 12-14

For peer review only

Supplementary Material 2

Interviewguide

**Background information intervention**
• What is the aim of [name intervention]?
• Is there a specific strategy, method or approach used in the interventions? Can you explain?<br>• Which target group/people can participate in [name intervention]? Why?<br>• Which target groups/people can nog participate in [name intervention]? Why?<br><br>[DEPENDING ON PRACTITIONER INVOLVEMENT OR NOT]<br>• What is the role of the professional?<br>• What does the contact between participant and professional look like?<br>• What expertise, or qualifications, must a professional have to provide [name intervention]? And why?
I am now going to ask you questions about your perspective on the working elements of [name intervention]and how it is provided. I would like to ask you to answer from your <u>own experience</u> as a professional.
**Working elements**
• What impact do you think [name intervention] has on the <u>actual alcohol consumption</u> of participants aged 55+?<br>• What impact do you think [name intervention] has on how people aged 55+<u> think about alcohol consumption?</u>
• In your opinion, what makes [name of intervention] cause [listed outcomes]?<br>• What do you think are working elements of the intervention?<br>For each element, ask the following things:<br>o What works well for whom? Can you explain this?<br>o To what extent do you think this works well? Can you explain this?<br>o How does this work? Can you explain?<br>o Why do you think this works well? Can you explain?
**Context**
• What is the impact of how [the support/name of intervention] is provided? By provided you can think of it being offered [individually/in a group] and [online/offline]?<br>o For whom do you think this mode works well? Can you explain this?<br>o To what extent do you think this works well? Can you explain?<br>o Why does this work well according to you? Can you explain?<br>o Why do you think this works well? Can you explain?<br><br>• What is the impact of the location where [the support/name of intervention] is provided?<br>o For whom do you think this location works well? Can you explain?<br>o To what extent do you think this works well? Can you explain?<br>o Why does it works well? Can you explain?<br>o Why do you think this works well? Can you explain?
**Relatives**
• To what extent do participants aged 55+ receive support from relatives during [name intervention]?<br>• o What does this support look like?<br>• What is the impact of support from relatives? Can you explain?<br>o For whom do you think support works well? Can you explain?<br>o To what extent do you think this support works well? Can you explain?<br>o Why do you think it works well? Can you explain?<br>o Why do you think this works well? Can you explain?
• What support would (further) be desirable during participation in [name intervention]? How do you think this could be achieved?
• How do you think relatives experience providing support?<br>• To what extent do you think relatives need help or support?

**CEMO statements**
I am going to present a few statements. I would like to invite you to respond to these statements.
Practitioner – in-person – individual (A)<br>• An intervention with face to face guidance from a professional can make people (55+) reduce or stop drinking.<br>o Definitely when there is a focus on drinking bheaviour, making people think about their own alcohol use.<br>o Definitely when the professional is very empathetic, making participant and professional work together in finding appropriate help and improving their relationship.
Practitioner – not in-person – individual (B)<br>• An intervention with no face-to-face guidance from a professional can make people (55+) reduce or stop drinking.<br>o Definitely when there is personal contact with feedback.<br>o Definitely when there is online communication with feedback.
Practitioner – in-person – relatives (C)<br>• An intervention with face to face guidance from a professional with relatives' involvement can make people (55+) reduce or stop drinking.<br>o Defenitely when the partner is taught to deal with the behavior of the drinking partner, this can lead to more understanding and support from the non-drinking partner for the drinking partner.
Practitioner – in-person – group component (D)<br>• An intervention with face-to-face guidance from a professional in a group setting can make people (55+) reduce or stop drinking.<br>o Definitely when people are motivated to make lifestyle changes.<br>o Definitely when this is delivered in a workplace setting.
No practitioner – not in-person – individual (E)<br>• An intervention without guidance from a professional can make people (55+) reduce or stop drinking. .<br>o Definitely in web based interventions<br>o Definitely with telephone based interventions.
No practitioner – not in-person – group component (F)<br>• An intervention without guidance from a professional in a group setting can make people (55+) reduce or stop drinking.<br>o Definitely when the intervention focuses on abstinention, which increases awareness of their alcohol consumption
Closing interview *(5 minutes)*
• We are including the following interventions in the study: Moti-55, Ikpas, the alcohol info line, Fris Verder, Healthy Enjoyment, Vitality Days, Maxx, the LessDrinking app and Mirro. To your knowledge, are there any other interventions for older adults (55+)?
• Is there anything else you wild like to add to the interview?
• We would like to publicly share the data and information from these interviews and questionnaire you have completed with other researchers. This will allow this information to be reused for new research in the future.<br>• Before we share this data, we will anonymise all information. This means that personal data or data that can be traced back to you as a person such as name, age or other info that reveals your identity will not be shared with them. Such information will be blacked out before the data is made public.<br>• Do you give permission to store the anonymised research data for an unlimited period and to make it publicly available to other researchers?<br>You are not required to give permission for this. Without permission, we will not make the data publicly available and it will not be used for follow-up research. Even if you do not give permission for this, your data can still be used for current research.
**Recorder off**
• I would like to thank you for the interview and the time you were willing to spend.

- You will be digitally sent a voucher worth 20 euros.
- Should there be any questions at a later moment, please contact me.
- Also, if you do not have any questions but feel the need to talk after, please contact me.

For peer review only
